# Supplementary material for: Management and outcomes of gastrointestinal congenital anomalies in low, middle and high income countries: protocol for a multicentre, international, prospective cohort study
Source: BMJ Open. 2019 Sep 3;9(8):e030452. doi: 10.1136/bmjopen-2019-030452 (PMC6731898; doi:10.1136/bmjopen-2019-030452)
Supplement: Supplementary data [file bmjopen-2019-030452supp001.pdf]

Management and Outcomes of Gastrointestinal Congenital Anomalies in Low-, Middle- and High-Income Countries: Protocol for a Multi-Centre, International, Prospective Cohort Study

SUPPLEMENTARY FILE 1: Condition Specific Data Points

Oesophageal Atresia (OA) +/- Tracheo-Oesophageal Fistula (TOF)

Question	Answers
Type of OA +/- TOF (Gross classification)	A, B, C, D, E<br>A: without a fistula, B: proximal TOF with distal OA, C: distal TOF with proximal OA, D: proximal and distal TOF, E: H-type TOF without OA.
Long or short gap?	Long, Short, Unknown<br>Long gap OA: A gap of 4 vertebral bodies or more. Anatomically cases either have no TOF or a gap of over 4 vertebral bodies following division of the distal fistula making primary repair unfeasible. Short OA: A gap of less than 4 vertebral bodies. Primary anastomosis typically feasible.
Pneumonia at presentation?	Yes: diagnosed clinically, Yes: diagnosed radiologically, Yes: other means of diagnosis, No: patient born in the study centre, No: patient born outside the study centre but no evidence of pneumonia on arrival<br>Pneumonia is defined as lung inflammation typically caused by bacterial or viral infection, in which the air sacs fill with pus and may become solid.
Primary intervention:	TOF ligation, Oesophageal anastomosis, Oesophagostomy, Gastrostomy, Ligation of the distal oesophagus, Gastro-oesophageal disconnection, Foker technique, Fundoplication, Other (please specify), Palliative care<br>Select all that apply.
If the patient had a primary oesophageal anastomosis, was a post-operative oesophagogram undertaken?	Yes, No. (At any stage)
If yes, routine or clinically indicated?	Routine, Clinically indicated
If yes, when?	Number of days after primary surgery
If yes, what was the result?	Leak, No leak
For patients diagnosed with a leak radiologically, was it associated with clinical symptoms?	Yes, No
Time to first oral feed post-operatively	In days. Please include the day of surgery and the first day of oral feeds in the calculation. Enter 0 if oral feeds were not commenced within 30-days of primary intervention. Do not include other types of enteral feeding such as nasogastric or gastrostomy feeding.
Time to full oral feeds	In days (enter 0 if the patient died before reaching full oral feeds or 30 if the patient had not reached full oral feeds at 30-days post primary intervention). Do not include other types of enteral feeding such as nasogastric or gastrostomy feeding.
For patient's not receiving a primary oesophageal anastomosis, at what age is definitive surgery planned?	In months (enter unknown if not planned or enter not applicable if primary anastomosis was undertaken).
For patient's not receiving a primary oesophageal anastomosis, what is the future planned procedure?	Gap assessment, Primary oesophageal anastomosis if possible, Gastric pull-up, Jejunal interposition, Colonic interposition, Not applicable: primary anastomosis undertaken, Other, Unknown. Select all that apply. If other, please specify.
If the patient had surgery, what was the approach?	Thoracotomy muscle cutting, Thoracotomy muscle splitting, Thoracoscopy, Laparotomy, Laparoscopy, Limited local incision, Other.<br>During primary surgery. If other, please specify.
If thoracoscopic or laparoscopic, was the surgery converted to open?	Yes, No
Did the patient have a condition specific complication within 30-days of primary intervention?	Pneumonia, Mediastinitis, Pneumothorax, Chylothorax, Haemothorax, Anastomotic leak, Anastomotic stricture, Recurrent TOF, Other, None<br>Select all that apply. If other, please specify.
Did the patient have tracheomalacia?	Yes: diagnosed clinically, Yes: diagnosed on bronchoscopy, Yes: diagnosed on CT, Yes: diagnosed on bronchogram, Yes: other method of diagnosis, No
If yes, was an intervention undertaken? If other, please specify	Yes: aortopexy, Yes: tracheostomy, Yes: tracheal stent, Yes: supportive management (oxygen +/- ventilation) only, Yes: other treatment, No

Congenital Diaphragmatic Hernia (CDH)

Question	Answers
Type of CDH.	Left posteriolateral (Bochdalek), Right posteriolateral (Bochdalek), Bilateral posteriolateral (Bochdalek), Central, Anterior (Morgagni), Other. If other, please specify.
Type of Bochdalek CDH (CDH Study Group Classification)	A, B, C, D, Other (specify), Unknown.<br>Defect A: smallest defect, usually "intramuscular" defect with >90% of the hemi-diaphragm present; this defect involves < 10% of the circumference of the chest wall. Defect B: 50-75% hemi-diaphragm present; this defect involves < 50% of

Wright NJ. *BMJ Open* 2019; 9:e030452. doi: 10.1136/bmjopen-2019-030452

If bilateral, what was the type of Bochdalek hernia on the left?<br>If bilateral, what was the type of Bochdalek hernia on the right?	the chest wall. Defect C: < 50% hemi-diaphragm present; this defect involves >50% of the chest wall. Defect D: largest defect (previously known as "agenesis"); complete or near complete absence of the diaphragm with < 10% hemi-diaphragm present; this defect involves >90% of the chest wall. Surgically, it is an absent posterior rim beyond the spine, absent posterior-lateral rim, and an anterior/anterior-medial rim which is miniscule. As it is truly unusual to have zero tissue at all, this is the CDHSG member consensus. "D" defects should all require a patch (or muscle flap) for repair.<br>A, B, C, D, Other, Unknown<br>If other, please specify.<br>A, B, C, D, Other, Unknown<br>If other, please specify.
If antenatally diagnosed, what was the lung-to-head ratio (LHR)?	Enter zero if not undertaken/ not known.
Was foetal tracheal occlusion (FETO) undertaken?<br>If yes, at what gestational age was it inserted?<br>If yes, at was gestational age was it removed?	Yes, No<br>_________, unknown.<br>_________, at birth, unknown.
Liver position?	Chest, Abdomen, Unknown
Did the patient have pulmonary hypertension (at any stage)?<br>If yes, treatment given? If other, please specify.	Yes: diagnosed clinically, Yes: diagnosis confirmed on echocardiography, Yes: other method of confirming diagnosis, No, Unknown<br>Persistent pulmonary hypertension of the newborn (PPHN) is defined as the failure of the normal circulatory transition that occurs after birth. It is a syndrome characterised by marked pulmonary hypertension that causes hypoxemia secondary to right-to-left extrapulmonary shunting of deoxygenated blood. It should be suspected whenever the level of hypoxemia is out of proportion to the level of pulmonary disease. Echocardiography plays a major role in screening and assisting in making the diagnosis of PPHN.<br>Nitric oxide, Prostacyclin, Alprostadil, Milrinone, Other, None: not required, None: required but not available.
Did the patient receive extracorporeal membrane oxygenation (ECMO)?<br>If yes, for how long?	Yes, No<br>In days. Include the day the patient went onto ECMO and the day they were taken off in the calculation.
Primary intervention<br>If patch repair, material used?<br>Other procedures undertaken at the same time?<br>Surgical approach:<br>If laparoscopic or thoracoscopic, was the surgery converted to open?<br>Condition specific complication within 30-days of primary surgery?	Primary repair (absorbable sutures), Primary repair (non-absorbable sutures), Patch repair, Palliation, Discharged with planned elective repair, Other<br>Permacol, PTFE, Alloderm, Dacron, Mesh plug, Muscle flap, Surgisis, Other. If other, please specify.<br>Chest drain insertion, Abdominal wall patch, Fundoplication, Correction of malrotation, Appendicectomy, Other (specify), None<br>Select all that apply. If other, please specify.<br>Laparotomy, Laparoscopy, Thoracotomy, Thoracoscopy, Other (please specify)<br>Yes/No.<br>Air leak (not just redundant space in the pleural cavity which is common), Chylothorax, Recurrence, Adhesional obstruction, Other, None. Select all that apply. If other, please specify.

Intestinal Atresia

Question	Answers
Type of intestinal atresia	Duodenal, Jejuno-ileal, Colonic
Classification of duodenal or colonic atresia<br>Classification of jejuno-ileal atresia	1,2,3,4<br>1) intraluminal web with continuity of the muscular layer, 2) atretic segment without a mesenteric defect, 3) atretic segment with mesenteric defect, 4) multiple atresias = string of sausages appearance.<br>1,2,3a,3b,4<br>1) intraluminal web with continuity of the muscular layer, 2) atretic segment without a mesenteric defect, 3a) atretic segment with mesenteric defect, 3b) apple-peel (bowel wrapped around a single artery), 4) multiple atresias = string of sausages appearance.
Primary intervention for duodenal atresia:<br>Surgical approach<br>Conversion to open procedure?<br>Type of anastomosis<br>Primary intervention for jejuno-ileal and colonic atresia:<br>If bowel was excised, what was the total length of bowel excised?<br>Surgical approach:<br>Conversion to open procedure?<br>Was the distal bowel flushed to check for patency?	Duodenoduodenostomy, Duodenojenunostomy, Web excision only, Palliation, Other. If other, please specify.<br>Laparotomy, Laparoscopy, Endoscopy, Other<br>Yes/ No<br>Kimura's diamond shape, Side-to-side, End-to-end<br>Primary anastomosis, Bowel resection, Division of web only, Loop stoma, Divided stoma, Bishop-Koop stoma, Santulli stoma, Palliation, Other. Select all that apply.<br>In centimetres (cm). Enter 0 if unknown<br>Laparotomy, Laparoscopy, Endoscopy, Other<br>Yes, No<br>Yes, No

Wright NJ. *BMJ Open* 2019; 9:e030452. doi: 10.1136/bmjopen-2019-030452

If the patient underwent surgery, did they have a condition specific complication within 30-days of primary intervention?	Anastomotic leak, Anastomotic stenosis, Short-gut, Missed additional atresia, Adhesive bowel obstruction, Stoma prolapse, Stoma retraction, Parastomal hernia, Parastomal skin breakdown, Other. If other, please specify. Select all that apply. For the purposes of this study short gut is defined as more than 50% of the small intestine excised (when short bowel syndrome can occur).

Gastroschisis

Question	Answers
Type of gastroschisis	Simple, Complex: associated with atresia, Complex: associated with necrosis, Complex: associated with perforation, Complex: associated with closing gastroschisis. Select all that apply.
Primary intervention:<br><br>Method of defect closure:<br><br>On what day following admission was abdominal wall closure achieved?<br><br>Did the neonate have any of these complications within 30-days of primary intervention?<br><br>If the patient has ACS, was the abdomen re-opened?	Primary closure in the operating room (OR), Primary closure at the cotside (Bianchi technique), Staged closure using a preformed silo, Staged closure using an Alexis Wound Retractor and Protector, Staged closure using a surgical silo (including improvised silo), Other method, No intervention undertaken. If other, please specify.<br>Fascia and skin closed with sutures, Just skin closed with sutures: fascia left open, Umbilical cord sutured over the defect: fascia left open, Sutureless closure with skin edges opposed and dressing applied, Dressing applied: defect left open to close by secondary intention, Other, Patient died before the defect was closed. If other, please specify.<br>In days. Please include the first day of admission and the day of closure in the calculation. For example, for a neonate admitted with gastroschisis on 2nd October who had the defect closed on 4th October, please insert 3 days.<br>Ischemic bowel, Abdominal compartment syndrome (ACS), Necrotising enterocolitis, None of these<br>Select all that apply. ACS is defined as respiratory insufficiency secondary to compromised tidal volumes, decreased urine output caused by falling renal perfusion or any other organ dysfunction caused by increased intra-abdominal pressure.<br>Yes/ No

Exomphalos

Question	Answers
Type of Exomphalos?	Major, Minor<br>Major: >50% of the liver in the exomphalos sac and abdominal wall defect >5cm. Minor: Infants with defects less than 5cm.
Hypoglycaemic on arrival?	Yes, No, Blood glucose not measured<br>Hypoglycaemia is defined as a blood glucose level below 4 mmol/L (72mg/dL).
Primary intervention<br>If the patient had a staged closure, what was the time from primary intervention to closure?<br><br>If conservative management, was a topical treatment applied to the exomphalos sac?<br>If conservative management was undertaken, what is the plan for future management?	Primary operative closure, Staged closure, Conservative management<br>In days. Please include the day of the primary intervention and the day of closure in the calculation. Enter 30 if still not closed at 30-days after primary intervention.<br>Yes: silver sulfadiazine, Yes: betadine, Yes: honey, Yes: merbromide tannage, Yes: other, no. If other, please specify.<br>No further surgery planned, Delayed closure at this hospital, Delayed closure at another hospital, Other. If other, please specify.
Did the patient have a ruptured sac?	Yes, No

Anorectal Malformation (ARM)

Question	Answers
Type of anorectal malformation (Krickenbeck classification)	Low ARM: Perineal (cutaneous) fistula, High ARM: Rectourethral fistula (bulbar), High ARM: Rectourethral fistula (prostatic), High ARM: Rectovesical fistula, High ARM: Vestibular fistula, High ARM: Cloaca, High ARM: No fistula, High ARM: Type unknown at present, Rare variant: Pouch colon, Rare variant: Rectal atresia/ stenosis, Rare variant: Rectovaginal fistula, Rare variant: H fistula, Other
Did the neonate have pre-operative bowel perforation?	Yes, No
What was the primary intervention undertaken?<br><br>If primary anorectal reconstruction was undertaken, was a Peña stimulator or equivalent used to identify the position of the muscle complex intra-operatively?<br>Did the patient have any of the following complications within 30-days of surgery?<br>- Electrolyte disturbance<br>- High output stoma (over 20mls/kg/day)	Fistula dilation: no surgery, Loop sigmoid colostomy, Divided sigmoid colostomy, Loop transverse colostomy, Divided transverse colostomy, Other stoma, Anoplasty, Posterior sagittal anorectoplasty (PSARP), Abdominosacroperineal pull-through, Abdominoperineal pull-through, Laparoscopic-assisted pull-through, Palliative care, Other. If other, please specify. Select all that apply.<br>Yes, no: equipment was not available, no: the equipment was available but not used. Peña stimulator: Muscle locating stimulator commonly used to identify the anal sphincter muscles whilst undertaking a PSARP for patients with ARM.<br>For each of the below answer: Yes, No, Not applicable

- Stoma prolapse/ retraction/ herniation<br>- Peri-stoma skin breakdown (or perianal if primary reconstructive surgery undertaken without a covering stoma)<br>- Anal stenosis in those undergoing primary anorectal reconstruction without covering stoma.	
What is the plan for future management?	No further operative management, Anoplasty/ pull-through planned at your hospital, Anoplasty/ pull-through planned at another hospital, Stoma closure planned at your hospital, Stoma closure planned at another hospital, Other<br>Please tick all that apply. If other, please specify.

Hirschsprung's Disease

Question	Answers
Time to first passage of meconium after birth	Less than 24 hours, 24-48 hours, Over 48 hours, Unknown
Features at presentation:	Abdominal distension, Bilious vomiting, Non-bilious vomiting, Poor feeding, Suspected enterocolitis, Perforation, Other. Select all that apply.
Source of diagnosis of Hirschsprung's disease<br><br>If on biopsy, what was the method of histology staining.	Genetic, Mucosal biopsy, Full thickness biopsy, Anorectal manometry, Barium enema, Not confirmed: suspected only, Other.<br>Hemotoxilin and Eosin (H&E), Acetylcholinesterase, Calretinin, Other.<br>Select all that apply. If other, please specify.
Length of aganglionosis:	Rectal, Sigmoid, Descending colon, Transverse colon, Ascending colon, Small bowel, Unknown at present
Primary intervention	Conservative: no treatment, Conservative: digital stimulation and laxatives, Conservative: regular rectal washouts/ enemas, Failed conservative management followed by a stoma during the same hospital admission, Primary stoma (with or without pre-operative washouts or enemas prior to a planned stoma placement), Primary pull-through (Swenson), Primary pull-through (Duhamel), Primary pull-through (Soave), Primary pull-through (Other), Transanal posterior anorectal myectomy, Palliative care, Other.
If primary pull-through was undertaken, did the patient have a covering stoma?	Yes, No
Was it laparoscopic assisted?	Yes, No
Did the patient have any condition specific complications within 30-days of primary intervention?	Hirschsprung's associated enterocolitis (HAEC), Electrolyte disturbance, High stoma output (over 20mls/kg/day), Stoma prolapse/ retraction/ herniation, Peri-stoma skin breakdown (or perianal if primary pull-through was undertaken without a covering stoma), Anal stenosis, Post-operative obstruction, Anastomotic leak (if primary pull-through was undertaken without a covering stoma), Other<br>HAEC is defined as inflammation of the small and or large bowel in patient's born with Hirschsprung's disease. If the patient was managed conservatively, please tick if they developed enterocolitis within 30-days of presentation. Select all that apply.
What is the plan for future management?	No further surgery planned, Anorectal pull-through at your hospital, Anorectal pull-through at a different hospital, Stoma closure, Other, Unknown

Wright NJ. *BMJ Open* 2019; 9:e030452. doi: 10.1136/bmjopen-2019-030452
