# Supplementary material for: Management and outcomes of gastrointestinal congenital anomalies in low, middle and high income countries: protocol for a multicentre, international, prospective cohort study
Source: BMJ Open. 2019 Sep 3;9(8):e030452. doi: 10.1136/bmjopen-2019-030452 (PMC6731898; doi:10.1136/bmjopen-2019-030452)
Supplement: Supplementary data [file bmjopen-2019-030452supp002.pdf]

Management and Outcomes of Gastrointestinal Congenital Anomalies in Low-, Middle- and High-Income Countries: Protocol for a Multi-Centre, International, Prospective Cohort Study

SUPPLEMENTARY FILE 2

Collaborator Survey: Resources and Facilities for Neonatal and Paediatric Surgery

Question	Answers
Title	Professor, Dr, Mr, Mrs, Miss, Ms, Other
Surname	
First Name	
Professional Position	Professor, Consultant or attending, Registrar or resident, Intern/ house officer/ senior house officer, Medical Student, Nurse, Other
Are you the study lead at your centre?	Yes, No<br>Answers provided by the study lead will be used as the 'Gold Standard' to which the other survey will be compared (this will remain anonymous).
Speciality	General Surgery (adult and paediatric), Paediatric Surgery, Anaesthetics, Paediatrics, Neonatology, Nursing, Not specialised yet, Other
Full name of institution:	
Address of institution:	
Country	Afghanistan, Albania, Algeria, Andorra, Angola, Antigua and Barbuda, Argentina, Armenia, Aruba (Kingdom of the Netherlands), Australia, Austria, Azerbaijan, Bahamas, Bahrain, Bangladesh, Barbados, Belarus, Belgium, Belize, Benin, Bhutan, Bolivia, Bosnia and Herzegovina, Botswana, Brazil, Brunei Darussalam, Bulgaria, Burkina Faso, Burundi, Cambodia, Cameroon, Canada, Cape Verde, Central African Republic, Chad, Chile, China, Colombia, Comoros, Congo, Costa Rica, Cote d'Ivoire, Croatia, Cuba, Curacao (Kingdom of the Netherlands), Cyprus, Czech Republic, Democratic Peoples Republic of Korea, Democratic Republic of the Congo, Denmark, Djibouti, Dominica, Dominican Republic, Ecuador, Egypt, El Salvador, Equatorial Guinea, Eritrea, Estonia, Eswatini (Swaziland), Ethiopia, Fiji, Finland, France, Gabon, Gambia, Georgia, Germany, Ghana, Greece, Grenada, Guatemala, Guinea, Guinea-Bissau, Guyana, Haiti, Holy See (Vatican City State), Honduras, Hong Kong (China), Hungary, Iceland, India, Indonesia, Iran (Islamic Republic of), Iraq, Ireland, Israel, Italy, Jamaica, Japan, Jordan, Kazakhstan, Kenya, Kiribati, Korea (Republic of), , Kuwait, Kyrgyzstan, Lao People's Democratic Republic, Latvia, Lebanon, Lesotho, Liberia, Libya, Liechtenstein, Lithuania, Luxembourg, Macau (China), Macedonia (the former Yugoslav Republic of), Madagascar, Malawi, Malaysia, Maldives, Mali, Malta, Marshall Islands, Mauritania, Mauritius, Mexico, Micronesia (Federated States of), Moldova (Republic of), Monaco, Mongolia, Montenegro, Morocco, Mozambique, Myanmar, Namibia, Nauru, Nepal, Netherlands, New Zealand, Nicaragua, Niger, Nigeria, Niue, Norway, Oman, Pakistan, Palau, Palestinian Territories, Panama, Papua New Guinea, Paraguay, Peru, Philippines, Poland, Portugal, Qatar, Republic of Kosovo, Reunion Island, Romania, Russian Federation, Rwanda, Saint Kitts and Nevis, Saint Lucia, Saint Vincent and the Grenadines, Samoa, San Marino, Sao Tome and Principe, Saudi Arabia, Senegal, Serbia, Seychelles, Sierra Leone, Singapore, Sint Maarten (Kingdom of the Netherlands), Slovakia, Slovenia, Solomon Islands, Somalia, Somaliland, South Africa, South Sudan, Spain, Sri Lanka, Sudan, Suriname, Swaziland (See Eswatini), Sweden, Switzerland, Syrian Arab Republic, Taiwan, Tajikistan, Tanzania (United Republic of), Thailand, Timor-Leste, Togo, Tonga, Trinidad and Tobago, Tunisia, Turkey, Turkmenistan, Tuvalu, Uganda, Ukraine, United Arab Emirates, United Kingdom, Uruguay, USA, Uzbekistan, Vanuatu, Venezuela, Viet Nam, Yemen, Zambia, Zimbabwe
Type of institution (WHO classification)	- **Specialised children's hospital** (Provides highly specialised care dedicated to children)<br>- **Referral hospital** (WHO defined tertiary healthcare. Includes academic, university, teaching, national, central and specialised mission hospitals. Can provide specialised surgical services)<br>- **District hospital** (WHO defined secondary healthcare. Includes provincial, general, general mission or regional hospitals. Has general anaesthesia and can provide general surgical care)<br>- **Health centre** (WHO defined primary healthcare. No general anaesthesia, can do minor local procedures, wound management, triage and referral).

Wright NJ. *BMJ Open* 2019; 9:e030452. doi: 10.1136/bmjopen-2019-030452

Institutional classification	Government, Non-government
Institutional financial classification	Not for profit, For profit
Population served by your institution (in millions, including children and adults)	
**Personnel**	
Number of Consultant Paediatric Surgeons undertaking neonatal surgery at your institution:	____________________ (Excluding trainees)
Number of Consultant General Surgeons (covering adults and children) undertaking neonatal surgery independently at your institution:	____________________ (Excluding trainees)
Number of medical officers or other non-surgeon healthcare professionals undertaking neonatal surgery independently at your institution	____________________ (Without a consultant surgeon present at the time of surgery)
**Infrastructure**<br>Please state whether the following facilities are available at your institution when required:	
Running Water	Always, Sometimes, Never
Electricity	Always, Sometimes, Never
Electricity generator back-up	Always, Sometimes, Never
Laboratory for biochemistry	Always, Sometimes, Never
Laboratory for haematology	Always, Sometimes, Never
Blood bank	Always, Sometimes, Never
Sterile gloves and gown	Always, Sometimes, Never
Autoclave for sterilising surgical equipment	Always, Sometimes, Never
Neonatal ventilation outside the operating room	Always, Sometimes, Never
Paediatric ventilation outside the operating room	Always, Sometimes, Never
Neonatal intensive care unit for surgical neonates pre and post operatively (including if a stoma is present)	Always, Sometimes, Never
Paediatric intensive care unit for surgical paediatric patients pre and post operatively if required	Always, Sometimes, Never
Parenteral nutrition for neonates	Always, Sometimes, Never
Parenteral nutrition for adults and older children	Always, Sometimes, Never
Extracorporeal membrane oxygenation (ECMO)	Always, Sometimes, Never
Peña stimulator or equivalent device to identify the muscle complex during anorectal reconstruction	Always, Sometimes, Never
Suction rectal biopsy gun to investigate for Hirschsprung's disease	Always, Sometimes, Never
**Procedures**<br>Please state whether the following procedures are available at your institution when clinically appropriate/ required:	
Neonatal laparotomy	Always, Sometimes, Never
Neonatal laparoscopy	Always, Sometimes, Never
Neonatal thoracotomy	Always, Sometimes, Never
Neonatal thoracoscopy	Always, Sometimes, Never
Neonatal central line insertion	Always, Sometimes, Never
Paediatric central line insertion	Always, Sometimes, Never
Umbilical vein catheterisation	Always, Sometimes, Never
Bedside primary reduction and closure of gastroschisis (Bianchi technique)	Always, Sometimes, Never
Preformed silo application, reduction and closure of gastroschisis	Always, Sometimes, Never
Surgical silo application, reduction and closure of gastroschisis	Always, Sometimes, Never
Primary closure of gastroschisis in the operating room	Always, Sometimes, Never
Sigmoid colostomy	Always, Sometimes, Never
Posterior Sagittal Anorectoplasty (PSARP) for anorectal malformation	Always, Sometimes, Never
Foetal tracheal occlusion (FETO) for CDH	Always, Sometimes, Never
Pull-through for Hirschsprung's disease	Always, Sometimes, Never
**Anaesthesia and resuscitation**<br>Please state whether the following facilities are available at your institution when required:	
Neonatal bag, valve and mask	Always, Sometimes, Never
Paediatric bag, valve and mask	Always, Sometimes, Never
Bottled oxygen	Always, Sometimes, Never
Piped oxygen	Always, Sometimes, Never
Oxygen saturation monitor	Always, Sometimes, Never
Apnoea monitor	Always, Sometimes, Never
Multi-parameter intra-operative monitoring	Always, Sometimes, Never
Anaesthetic machine for neonates	Always, Sometimes, Never
Anaesthetic machine for children	Always, Sometimes, Never
Ketamine anaesthesia for neonates	Always, Sometimes, Never
Ketamine anaesthesia for children	Always, Sometimes, Never
Spinal/ caudal anaesthesia for neonates	Always, Sometimes, Never
Spinal/ caudal anaesthesia for children	Always, Sometimes, Never

Wright NJ. *BMJ Open* 2019; 9:e030452. doi: 10.1136/bmjopen-2019-030452

Anaesthetic doctor competent to perform neonatal anaesthesia	Always, Sometimes, Never
Anaesthetic doctor competent to perform paediatric anaesthesia	Always, Sometimes, Never
Anaesthetic nurse competent to perform neonatal anaesthesia	Always, Sometimes, Never
Anaesthetic nurse competent to perform paediatric anaesthesia	Always, Sometimes, Never
Does your country have at least one specialised children's hospital that can provide neonatal and paediatric surgery?	Yes, No
Any other comments?	

Wright NJ. *BMJ Open* 2019; 9:e030452. doi: 10.1136/bmjopen-2019-030452
