# Supplementary material for: Management and outcomes of gastrointestinal congenital anomalies in low, middle and high income countries: protocol for a multicentre, international, prospective cohort study
Source: BMJ Open. 2019 Sep 3;9(8):e030452. doi: 10.1136/bmjopen-2019-030452 (PMC6731898; doi:10.1136/bmjopen-2019-030452)
Supplement: Supplementary data [file bmjopen-2019-030452supp003.pdf]

Management and Outcomes of Gastrointestinal Congenital Anomalies in Low-, Middle- and High-Income Countries: Protocol for a Multi-Centre, International, Prospective Cohort Study

SUPPLEMENTARY FILE 3: Study approval details of participating centres

Number	Country	Institution Name	Ethical/Audit Review Board Name	Approval Type	Approval Reference Number
**Low-Income Countries (LICs)**					
1	Afghanistan	Ataturk National Children's Hospital	Islamic Republic of Afghanistan Ministry of Public Health, Afghanistan National Public Health Institute, Institutional Review Board	Local (Ethical)	444738
2	Afghanistan	French Medical Institute for Mothers and Children (FMIC)	Institutional Review Board	Local (Ethical)	-
3	Afghanistan	Ghalib University Hospital	Ministry of Higher Education, Ghalib Cadre Hospital	Local (Ethical)	-
4	Afghanistan	Ghor Provincial Hospital	EPHS Directorate, GD of Curative Medicine, Ministry of Public Health	Local (Ethical)	-
5	Afghanistan	Indra Ghandi Institute of Child Health (IGICH)	Institutional Review Board	Local (Ethical)	-
6	Afghanistan	Irena Salimi Children Hospital (ISCH)	Institutional Review Board	Local (Ethical)	-
7	Burundi	Bujumbura, Kamenge Military Hospital	Head of Department Surgery	Local (Departmental)	-
8	Burundi	District Hospital Kibimba, Gitega	Medical Director of Kibimba Hospital	Local (Hospital)	4000342958
9	Burundi	Gitega Hospital Mutoyi	Hospital Director	Local (Hospital)	-
10	Burundi	Kibuye Hope Hospital, Hope Africa University, Gitega	Head of Surgery and Ethics Team	Local (Ethical and Hospital)	-
11	Burundi	King Khaled Teaching Hospital	Medical Director General	Local (Hospital)	2019|DECHUK.359|11.5
12	Burundi	Teaching Hospital University of Kamenge	Medical Director General	Local (Hospital)	2019|DECHUK.271|11.5
13	Burundi	Van Norman Clinic Bjumbura	Medical Director of Kibimba Hospital	Local (Hospital)	-
14	Democratic Republic of Congo	HEAL Africa Hospital, Goma	Institutional Review Board at HEAL Africa	Local (Ethical)	-
15	Ethiopia	Addis Ababa University	Deputy Head of Department of Surgery	Local (Departmental)	-
16	Gambia	Edward Francis Small Teaching Hospital, Banjul	Chair of Gambia Government/MRCG Joint Ethics Committee	Local (Ethical)	-
17	Gambia	Kanifing General Hospital, Kanifing	Gambia Government / MRCG Joint Ethics Committee	Regional (Ethical)	-
18	Nepal	Annapurna Children and Women's Hospital	Medical Director	Local (Hospital)	-
19	Niger	National Hospital Lamorde, Niamey	Hospital Director General	Local (Hospital)	000306
20	Rwanda	Centre Hospitalier Universitaire de Butare	College of Medicine and Health Science Institutional Review Board	Local (Ethical)	301/CMHS IRB/2018
21	Rwanda	Rwanda Military Hospital	College of Medicine and Health Science Institutional Review Board	Local (Ethical)	301/CMHS IRB/2018
22	Rwanda	University Teaching Hospital of Kigali	College of Medicine and Health Science Institutional Review Board	Local (Ethical)	301/CMHS IRB/2018
23	Somaliland	Hargeisa Group Hospital	Director of Hargeisa Group Hospital	Local (Hospital)	HGH/800/0.1/18
24	Tanzania	Kilimanjaro Christian Medical Centre	Executive Director	Local (Hospital)	-
25	Zimbabwe	Mpilo Hospital	Clinical Director of Mpilo Central Hospital	Local (Hospital)	-
**TOTAL LICs:** 25					

Wright NJ. *BMJ Open* 2019; 9:e030452. doi: 10.1136/bmjopen-2019-030452

**Middle-Income Countries (MICs)**					
26	Algeria	Hôpital Mère-Enfant EHS EL EULMA	Institutional Ethical Committee of Ferhat ABBAS University SETIF	Local (Ethical)	-
27	Algeria	Hospital Chu Tizi-ouzou	Ethical Committee of the University Mouloud Maameri de Tizi-ouzou	Local (Ethical)	-
28	Algeria	Le Nefissa Hamoud University Hospital	Institutional Ethics Comittee	Local (Ethical)	-
29	Angola	Pediatric Hospital David Bernardino	Hospital Director	Local (Hospital)	55/09/DIRPED/HPDB/018
30	Argentina	Children’s Hospital of Santa Fe	Associate Director of Teaching and Research	Local (Hospital)	-
31	Argentina	Clinica Pueyrredon	Medical Director	Local (Hospital)	-
32	Argentina	Dr Humberto Notti Pediatric Hospital of Mendoza	Executive Director	Local (Hospital)	3542-D-2018-04238-E-0-6
33	Argentina	Fundación Dr JR Villavicencio	Clinical Investigation Ethical Committee	Local (Ethical)	-
34	Argentina	Fundacion Hospitalaria	Chief of Department of Paediatric Surgery	Local (Departmental)	-
35	Argentina	Hospital de Niños Victor J. Vilela, Rosario, Santa Fe	President of the Ethics Committee in Research	Local (Ethical)	-
36	Argentina	Hospital Escuela Eva Perón, Rosario	Medical Director	Local (Hospital)	30630225
37	Argentina	Hospital Italiano de Buenos Aires, Buenos Aires	Ethical Committee	Local (Ethical)	139/DGDOIN/14 Anexo 1
38	Argentina	Hospital Provincial de Rosario	Research Ethics Committee (CEI)	Local (Ethical)	-
39	Argentina	Hospital Público Materno Infantil	President of the Advisory Commission on Teaching and Investigation	Local (Hospital)	-
40	Argentina	Hospital Rawson, San Juan	Ethics committee in research	Local (Ethical)	33/18
41	Argentina	Maternidad Martin, Rosario, Santa Fe	President of the Ethics Committee in Research	Local (Ethical)	-
42	Argentina	Maternity Clinic Colón	Teaching and Research committee	Local (Hospital)	-
43	Argentina	Materno Provincial	Health Minister, Training, Teaching and Investigation Committee	Local (Hospital)	-
44	Argentina	Maternoneonatal, Córdoba	Surgical Director	Local (Departmental)	-
45	Argentina	Pediatric Hospital of Santiago del Estero	Deputy Medical Director	Local (Hospital)	-
46	Argentina	Zona Norte Children Hospital	Medical Director	Local (Hospital)	-
47	Bangladesh	Bangabandhu Sheik Mujib Medical University (BSMMU), Dhaka	Chairman, Department of Paediatric Surgery	Local (Departmental)	-
48	Bangladesh	Dhaka Medical College Hospital	Ethical Committee	Local (Ethical)	MEU-DMC/ECC/2018/249
49	Bangladesh	Dhaka Shishu Hospital	Hospital Authority	Local (Hospital)	-
50	Belarus	Republican Scientific Practical Centre of Pediatric Surgery, Minsk	Ethical Council	Local (Ethical)	1-18/394
51	Bolivia	Children's Hospital Manuel A.Villarroel Cochabamba	Medical Director	Local (Hospital)	-
52	Bolivia	Hospital Hernandez Vera, Santa Cruz	Medical Director	Local (Hospital)	-
53	Bolivia	Hospital San Juan de Dios, Tarija	Medical Director	Local (Hospital)	-
54	Bolivia	Mario Ortiz Children's Hospital Suarez, Santa Cruz	Medical Director	Local (Hospital)	-
55	Bolivia	Women’s Hospital Percy Boland Rodriguez Santa Cruz	Medical Director	Local (Hospital)	-
56	Bosnia and Herzegovnia	Clinic of Paediatric Surgery, Clinical Centre University of Sarajevo, Sarajevo	Ethical Committee	Local (Ethical)	03-02-47488 (Protocol: 0602-40400)
57	Brazil	Associação de Ensino Superior de Nova Iguaçu	National Ethical Committee	National (Ethical)	3.130.042

58	Brazil	Associação Hospitalar de Prot Infancia Dr. Raul Carneiro	National Ethical Committee	National (Ethical)	3.130.042
59	Brazil	Botucatu Medical School, State of Sao Paulo University	National Ethical Committee	National (Ethical)	3.130.042
60	Brazil	Centro de Ensino São Lucas Ltda/ RO	National Ethical Committee	National (Ethical)	3.130.042
61	Brazil	Clinical Hospital from Federal University of Uberlandia	National Ethical Committee	National (Ethical)	3.130.042
62	Brazil	Federal University of Minas Gerais	National Ethical Committee	National (Ethical)	3.130.042
63	Brazil	Federal University of Parana	National Ethical Committee	National (Ethical)	3.130.042
64	Brazil	Fundação Facudade Regional de Medicina S J Rio Preto	National Ethical Committee	National (Ethical)	3.130.042
65	Brazil	Fundação Hospitalar Blumenau: Hospital Santo Antoni	National Ethical Committee	National (Ethical)	3.130.042
66	Brazil	Hospital da Criança Santo Antônio – Santa Casa/RS	National Ethical Committee	National (Ethical)	3.130.042
67	Brazil	Hospital da Luz/ São Paulo – SP	National Ethical Committee	National (Ethical)	3.130.042
68	Brazil	Hospital de Base Dr. Ary Pinheiro, Porto Velho	National Ethical Committee	National (Ethical)	3.130.042
69	Brazil	Hospital do Rocio	National Ethical Committee	National (Ethical)	3.130.042
70	Brazil	Hospital Federal de Bonsucesso	National Ethical Committee	National (Ethical)	3.130.042
71	Brazil	Hospital Infantil Jona de Gusmão/ SES – SC	National Ethical Committee	National (Ethical)	3.130.042
72	Brazil	Hospital Jorge Valente/ Salvador - BA	National Ethical Committee	National (Ethical)	3.130.042
73	Brazil	Hospital Materno Infantil de Brasília – HMIB	National Ethical Committee	National (Ethical)	3.130.042
74	Brazil	Hospital Nossa Senhora da Conceição SA	National Ethical Committee	National (Ethical)	3.130.042
75	Brazil	Hospital Santo Antônio Blumenau Santa Catarina	National Ethical Committee	National (Ethical)	3.130.042
76	Brazil	Hospital Universitário de Santa Maria	National Ethical Committee	National (Ethical)	3.130.042
77	Brazil	Hospital Universitário do Oeste do Paraná	National Ethical Committee	National (Ethical)	3.130.042
78	Brazil	Hospitalar Municipal do M’boi Mirim/ São Paulo - SP	National Ethical Committee	National (Ethical)	3.130.042
79	Brazil	Instituto de Medicina Integral Professor Fernando Figueira	National Ethical Committee	National (Ethical)	3.130.042
80	Brazil	Instituto Fernandes Figueira, Rio de Janeiro	National Ethical Committee	National (Ethical)	3.130.042
81	Brazil	Irmandade da Santa Casa de Misericórdia de Santos	National Ethical Committee	National (Ethical)	3.130.042
82	Brazil	Irmandade Nossa Senhora das Merces de Montes Claros	National Ethical Committee	National (Ethical)	3.130.042
83	Brazil	Instituto Fernandes Figueira, Rio de Janeiro	National Ethical Committee	National (Ethical)	3.130.042
84	Brazil	SPDM – Associação Paulista para o Desenvolvimento da Medicina	National Ethical Committee	National (Ethical)	3.130.042
85	Brazil	Universidade Estadual de Montes Claros - UNIMONTES	National Ethical Committee	National (Ethical)	3.130.042
86	Brazil	Universidade Federal de São Paulo – UNIFESP/EPM	National Ethical Committee	National (Ethical)	3.130.042
87	Brazil	University Hospital, Porto Alegre	National Ethical Committee	National (Ethical)	3.130.042
88	Cameroon	Mbingo Hospital in North West Cameroon	Institutional Review Board	Local (Ethical)	-
89	China	Bayi Children’s Hospital	Research Ethics Criteria	Local (Ethical)	-
90	China	Children's Hospital of Shanghai	In Chinese script	Local (Ethical)	-

Wright NJ. *BMJ Open* 2019; 9:e030452. doi: 10.1136/bmjopen-2019-030452

91	China	Children's Hospital of Soochow University	President of the Seventh Medical Center of PLA General Hospital	Local (Ethical)	-
92	China	Children's Hospital, Zhejiang University School of Medicine, Binjiang District, Hangzhou	Hospital President and Ethics Committee	Local (Ethical)	-
93	China	First Affiliated Hospital of Xinjiang Medical University	Research Ethics Criteria	Local (Ethical)	-
94	China	Hunan Children's Hospital	In Chinese script	Local (Ethical)	-
95	China	Jinhua Municipal Central Hospital	Research Ethics Committee	Local (Ethical)	-
96	China	Kunming Children's Hospital	Medical Ethics Committee	Local (Ethical)	-
97	China	Nanjing Children's Hospital	Research Ethics Criteria	Local (Ethical)	-
98	China	Ningbo Women Children's Hospital	Research Ethics Criteria	Local (Ethical)	-
99	China	Shengjing Hospital	In Chinese script	Local (Ethical)	-
100	China	Wenzhou Medical University Affiliate 2nd Hospital and Yuying Children Hospital	Research Ethics Criteria	Local (Ethical)	-
101	China	Wuhan Children's Hospital	In Chinese script	Local (Ethical)	-
102	Colombia	Bogota Hospital Militar Central	Director General	Local (Hospital)	2018046
103	Colombia	Bogota Hospital San Jose Centro Society for Surgery	President of Society	Local (Departmental)	-
104	Colombia	Fundacion Valle de Lili Cali Colombia	Research and Biomedical Ethics Committee	Local (Ethical)	234-2018
105	Colombia	Materno Infantil Saludcoop, Villavicencio	Medical Director	Local (Hospital)	-
106	Dominican Republic	Hospital Infantil Dr. Robert Reid Cabral, Santa Domingo	Investigational Committee	Local (Hospital)	
107	Ecuador	Hospital Especialidades Carlos Andrade Martin, Quito	Technical Director	Local (Hospital)	IESS-HJCA-DT-2018-5098-M
108	Ecuador	Hospital de Especialidades José Carrasco Arteaga, Cuenca	Hospital Director	Local (Hospital)	-
109	Ecuador	Hospital de Los Valles	Medical Director	Local (Hospital)	-
110	Ecuador	Hospital General del Norte de Guayaquil	Deputy Director of Teaching	Local (Hospital)	IESS-HG-NGC-DO-2018-0008-O
111	Ecuador	Omni Hospital, Gayaquil	Director of Teaching	Local (Hospital)	-
112	Egypt	Al Zahra Hospital	In Arabic script	Local (Ethical)	-
113	Egypt	Alexandria University Children's Hospital	Ethics Committee	Local (ethical)	0304041
114	Egypt	Assiut University Hospital	In Arabic script	Local (Ethical)	-
115	Egypt	Mansoura University Children's Hospital	Head of Department of Pediatric Surgery Council	Local (Departmental)	0032610/2020/81A
116	Egypt	Nasser Institution for Research and Treatment, Cairo	Hospital Director Letter	Local (Ethical)	-
117	Egypt	Tanta University Hospital	In Arabic script	Local (Ethical)	-
118	Gabon	Bongolo Hospital, Lebamba	Institutional Review Board at Bongolo Hospital	Local (Ethical)	-
119	Ghana	Korle-Bu Teaching Hospital	Scientific and Technical Committee & KBTH Institutional Review Board	Local (Ethical)	KBTH/MIS/G3/19
120	Ghana	Tamale Teaching Hospital	Head of Research and Development	Local (Ethical)	TTH/R&D/SR/18/8
121	India	Bazaricherra, Karimganj District	Chairman Research Committee	Local (Ethical)	-
122	India	Christian Medical college Ludhiana, Punjab	Institutional Research Committee	Local (Ethical)	-
123	India	Kasturba Medical College (Kasturba Hospital) Manipal	Institutional Ethics Committee	Local (Ethical)	ECR/146/Inst/KA/2013/RR-16
124	India	Makunda Christian Leprosy and General Hospital, Bazaricherra, Karimganj District	Chairman of Research Committee	Local (Ethical)	-

Wright NJ. *BMJ Open* 2019; 9:e030452. doi: 10.1136/bmjopen-2019-030452

125	Indonesia	Dr Soetomo Hospital, Surabaya	President of Indonesian Association of Pediatric Surgeons and Head of Institutional Review Board	Local (Association and Ethical)	83/PERBANI/XI/2018 IRB/22/04/ETIK/2019
126	Indonesia	Rumah Sakit Anak dan Bunda Harapan Kita Jakarta	Indonesian Association of Pediatric Surgeons president approval	Local and Regional	-
127	Iran	Mofid Children’s Hospital (MCH)	Research Council of Research Institute for Children Health	Local (Ethical)	0477, 162
128	Iraq	Al-Mustansiriyah University Central	Ministry of Higher Education and Scientific Research	Local (Ethical)	239
129	Iraq	Child's Central Teaching Hospital, Baghdad/Mustansiriyah Medical College	Ethical Committee	Local (Ethical)	-
130	Iraq	University of Kufa	Medical Ethics Committee for University	Local (Ethical)	MEC-035
131	Jordan	Al-Basheer Hospital	Al-Basheer Hospital and Chairman of Pediatric Surgery	Local (Departmental, Hospital)	15999
132	Jordan	Jordon University Hospital of Medicine	Institutional Review Board	Local (Ethical)	10/2018/2448
133	Jordan	JUST King Abdallah University Hospital	Institutional Review Board	Local (Ethical)	4/118/2018
134	Kenya	Aga Khan University Hospital	Research Ethics Committee, Aga Khan University	Local (Ethical)	2018/REC-138 (v1)
135	Kenya	Joromongi Oginga Odinga Teaching & Referral Hospital, Kisumu	JOOTRH Institutional Ethics and Research Committee	Local (Ethical)	ERC.IB/VOL.1/475
136	Kenya	University of Nairobi and Kenyatta National Hospital	Kenyatta National Hospital – University of Nairobi Ethics and Research Committee	Local (Ethical)	KNH-ERC/A/6
137	Libya	Al Fardous Clinic	Ethical Approval from Head of Medical Services	Local (Ethical)	2/018 P.
138	Libya	Alaml Specialised Hospital, Misurata	Ethical Approval from Head Director of Alaml Specialized Hospital	Local (Ethical)	-
139	Libya	Asalam, Misurata	In local script	Local (Ethical)	-
140	Libya	Assafwa international hospital, Misurata	Ethical Approval from Head of Medical Service	Local (Ethical)	-
141	Libya	Dar Alhekma Hospital	Head Director of Dar Alhekma Specialized Hospital	Local (Ethical)	-
142	Libya	Ghout El-Shaal Specialized Hospital	In local script	Local (Ethical)	-
143	Libya	Misarata Cancer Centre	Director of Medical Affairs, Chief of Medical Ethics	Local (Ethical)	708/42.
144	Libya	Misurata Medical Centre	Medical Director	Local (Ethical)	-
145	Libya	Tripoli Children’s Hospital	Ethical Committee	Local (Ethical)	NCDC:E17:2018.
146	Libya	Tripoli Medical Centre	Hospital Director	Local (Hospital)	-
147	Macedonia	University Clinic for Paediatric Surgery	Ethical Committee	Local (Ethical)	-
148	Malaysia	Hospital Canselor Tuanku Muhriz, UKM Medical Centre	Research Ethics Committee	Local (Ethical)	UKM PPI/111/8/JEP-2018-464
149	Malaysia	Hospital Kuala Lumpar	Medical Research and Ethics Committee of Ministry of Health Malaysia	Regional (Ethical)	KKM/NIHSEC/ P18-1816 (14)
150	Malaysia	Hospital Pulau Pinang	Medical Research and Ethics Committee of Ministry of Health Malaysia	Regional (Ethical)	KKM/NIHSEC/ P18-1816 (14)
151	Malaysia	Hospital Raja Perempuan Zainab II, Kota Bharu, Kelantan	Medical Research and Ethics Committee of Ministry of Health Malaysia	Regional (Ethical)	KKM/NIHSEC/ P18-1816 (14)
152	Malaysia	Hospital Sultanah Aminah, Johor Bharu, Johor	Medical Research and Ethics Committee of Ministry of Health Malaysia	Regional (Ethical)	KKM/NIHSEC/ P18-1816 (14)
153	Malaysia	Hospital Sultanah Nur Zahirah, Kuala Terengganu, Terengganu	Medical Research and Ethics Committee of Ministry of Health Malaysia	Regional (Ethical)	KKM/NIHSEC/ P18-1816 (14)
154	Malaysia	Hospital Tengku Ampuan Afzan, Kuantan	Medical Research and Ethics Committee of Ministry of Health Malaysia	Regional (Ethical)	KKM/NIHSEC/ P18-1816 (14)
155	Malaysia	Hospital Wanita dan Kanak-Kanak, Likas, Sabah	Medical Research and Ethics Committee of Ministry of Health Malaysia	Regional (Ethical)	KKM/NIHSEC/ P18-1816 (14)

Wright NJ. *BMJ Open* 2019; 9:e030452. doi: 10.1136/bmjopen-2019-030452

156	Malaysia	Penang General Hospital	Ethics & Medical research Committee	Local (Ethical)	KKM/NIHSEC/P18-1816(11)
157	Mexico	Hospital General Tijuana, Baja California	Research and Ethics Committee	Local (Ethical)	-
158	Mexico	Hospital Civil de Guadalajara	Research Committee and Ethics Committee	Local (Ethical)	0247/18 HCJIM/2018 and HCG/CEI-0913/18
159	Mexico	Hospital de Especialidades del Nino y Mujer de Queretaro	Research Committee	Local (Hospital)	135/10-10-2018/CIR.PED / HENM
160	Mexico	Hospital del Niño Poblano	Research Committee	Local (Hospital)	HNP 2018-16
161	Mexico	Hospital Infantil de Mexico Federico Gomez	Committee of Research, Ethics and Biosecurity	Local (Ethical)	HIM 2018-043
162	Mexico	Hospital Regional de Alta Especialidad del Niño “Dr. Rodolfo Nieto Padrón”	Research and Ethics Committee	Local (Ethical)	DI/051/18
163	Mexico	Moctezuma Children’s Hospital	Research and Ethics Committee	Local (Ethical)	09-CEI-006-20180328
164	Mexico	Pediatric Hospital, Western Medical Center, Mexican Institute of Social Security	SIRELCIS Administration	Local (Hospital)	F-CNNIC-2018-209
165	Morocco	Centre Hospitalier Universaitaire IBN Sina de Rabat, Rabat	Chief of Paediatric Emergency Services	Local (Departmental)	-
166	Nigeria	Amino Kano Teaching Hospital	Research Ethics Committee	Local (Ethical)	LAKTH/MAC/SUB/12A/ P-3/VI/2402
167	Nigeria	Bowen University Teaching Hospital	Ethics Committee	Local (Ethical)	NHREC/12/04/2012
168	Nigeria	Federal Medical Centre Abeokuta	Health Research Ethics Committee	Local (Ethical)	FMCA/470/19
169	Nigeria	Federal Medical Centre Umuahia	Health Research Ethics Committee	Local (Ethical)	FMC/QEH/G.596/Vol.10/ 371
170	Nigeria	Federal Medical Centre Yola	Research and Ethics Committee	Local (Ethical)	FMCY/SUB/S.128/042
171	Nigeria	Federal Teaching Hospital Gombe	Health Research Ethics Committee	Local (Ethical)	NHREC/25/10/2013
172	Nigeria	Lagos State University Teaching Hospital	Health Research Ethics Committee of LASUTH (LREC)	Local (Ethical)	NHREC04/04/2008
173	Nigeria	Lagos University Teaching Hospital	Lagos University Teaching Hospital Health Research Ethics Committee (LUTHHREC)	Local (Ethical)	NHREC 19/12/2008a
174	Nigeria	National Hospital Abuja	Institute Review Board Committee	Local (Ethical)	NHA/EC/054/2018
175	Nigeria	Nnamdi Azikiwe University Teaching hospital	Ethics Committee	Local (Ethical)	NAUTH/CS/668/VOL.2 /024 and NAUTH/CS/66/VOL.11/ 171/2018/107
176	Nigeria	Olabisi Onabanjo University Teaching Hospital	Health Research Ethics Committee	Local (Ethical)	NHREC/28/11/2017
177	Nigeria	University College Hospital, Ibadan	Ethics Committee and Head of Department of Surgery	Local (Ethical)	UI/EC/18/0432
178	Nigeria	University of Abuja Teaching Hospital	Health Research Ethics Committee	Local (Ethical)	UATH/HREC/PR/2019/001
179	Nigeria	University of Nigeria Teaching Hospital	Health Research Ethics Committee	Local (Ethical)	UNTH/CSA/329/OL5
180	Pakistan	Aga Khan University	Ethics Review Committee	Local (Ethical)	2019-0454-2658
181	Pakistan	Children's Hospital, PIMS, and Shaheed Zulfiqar Ali Bhutto Medical University, Islamabad	Ethical Review Board	Local (Ethical)	1-1 /2015 /ERB /SZABMU /274
182	Pakistan	Karachi Indus hospital	Interactive Research and Development Institute Review Board	Local (Ethical)	IRD_IRB_2018_07_012
183	Pakistan	Lahore King Edward Medical University	Institute Review Board	Local (Ethical)	109/RC/KEMU
184	Pakistan	Liaquat National Hospital	Ethics Review Committee	Local (Ethical)	0434-2018 LNH-ERC
185	Pakistan	The Children’s Hospital and the Institute of Child Health	Institute Review Board	Local (Ethical)	45868
186	Palestine, Gaza	Al-Aqsa Hospital, Gaza Strip	Helsinki Committee for Ethical Approval	National (ethical)	PHRC/HC/41//18
187	Palestine, Gaza	Al-Shifa Hospital, Gaza Strip	Helsinki Committee for Ethical Approval	National (ethical)	PHRC/HC/41//18
188	Palestine, Gaza	European Gaza Hospital, Gaza Strip	Helsinki Committee for Ethical Approval	National (ethical)	PHRC/HC/41//18
189	Palestine, West Bank	Ahli Hospital, Hebron	In Arabic script	National/Local (ethical)	-

190	Palestine, West Bank	Jenin Government Hospital, Jenin	In Arabic script	National/Local (ethical)	-
191	Palestine, West Bank	Mount of Olives/Makassed, Jerusalem	In Arabic script	National/Local (ethical)	-
192	Palestine, West Bank	Palestinian Medical Complex (PMC), Ramallah	In Arabic script	National/Local (ethical)	-
193	Palestine, West Bank	Rafidia Surgery Hospital, Nablus	In Arabic script	National/Local (ethical)	-
194	Palestine, West Bank	Red Crescent Society, Hebron	In Arabic script	National/Local (ethical)	-
195	Papua New Guinea	Mount Hagen General Hospital	Director Clinical Excellence	Local (Hospital)	-
196	Papua New Guinea	Port Moresby General Hospital	Director Medical Services and Ethical Committee	Local (Ethical)	-
197	Peru	Cayetano Heredia Hospital	Institutional Committee of Ethics and Investigation	Local (Ethical)	-
198	Peru	Guillermo Almenara National Hospital, Lima	Ethical Committee	Local (Ethical)	174-2018
199	Peru	Hospital Dos de Mayo	Ethics committee in biomedical research	Local (Ethical)	085-2018-CEIB-HNDM
200	Peru	Hospital Nacional Alberto Sabogal Sologuren	Institutional committee on ethics in research	Local (Ethical)	549-OAIyD-HNASS-ESSALUD-2018<br>79-UCP-DC-GQ-HNASS-ESSALUD-2018
201	Peru	Hospital Nacional Docente Madre Niño San Bartolomé	Committee of Institutional Ethics and Research	Local (Ethical)	12200-18
202	Peru	Hospital Nacional Edgardo Rebagliati Martins	Ethics Committee	Local (Ethical)	943-OCIYD-GHNERM-GRPR-ESSALUD-2018
203	Peru	Hospital Regional Del Cusco	Research committee and training unit	Local (Hospital)	8868-18.
204	Peru	Instituto Nacional de Salud Del Niño	Institutional Committee of Ethics and Investigation	Local (Ethical)	PI-64/18
205	Peru	Instituto Nacional de Salud de Nino de San Borja	Institutional Committee of Ethics and Investigation	Local (Ethical)	PI-2018-246
206	Peru	Instituto Nacional Materno Perinatal	Health Minister	Local (Hospital)	699-2018-DEN/INMP
207	Peru	Pediatric Emergencies Hospital	Head of the Teaching and Research Support Office	Local (Hospital)	065-2018-OADI-HEP/MINSA
208	Philippines	Philippines Children's Medical Center	Institutional Review Board – Ethics Committee	Local (Ethical)	PCMC IRB-EC 2018-040
209	Romania	Children Clinical Hospital of Brasov, Brasov/University of Brasov	Ethical Committee	Local (Ethical)	-
210	Romania	Marie Curie Hospital in Bucharest	Ethical Committee	Local (Ethical)	-
211	Serbia	Clinical Centre for Paediatric Surgery and Orthopaedics, Clinical Center Niš	Ethics Committee of the Faculty of Medicine University of Niš President	Local (Ethical)	12-3182-2/11
212	Serbia	University Children's Hospital	Ethics Committee	Local (Ethical)	14/390
213	South Africa	Frere Hospital	Clinical Governance Committee and Frere and Cecilia Makiwane Hospitals Research Ethics Committee	Local (Ethical)	FCMHREC/013/2019
214	South Africa	Greys Hospital, Pietermaritzburg	Subcommittee of Biomedical Research Ethics Committee	Local (Ethical)	BCA221/13
215	South Africa	Grootte Schurr Hospital	Human Research Ethics Committee and Medical Services Manager	Local (Ethical)	572/2018 and RCC158
216	South Africa	Red Cross War Memorial	Human Research Ethics Committee and Medical Services Manager	Local (Ethical)	572/2018 and RCC158
217	South Africa	Tygerberg Children's Hospital	Human Research Ethics Committee and Medical Services Manager	Local (Ethical)	N18/09/101
218	Sri Lanka	Lady Ridgeway Hospital for Children	Ethical Committee	Local (Ethical)	-
219	Sudan	Khartoum University Hospital	Head of Department of Paediatric Surgery	Local (Departmental)	-
220	Syria	Aleppo University Hospital	Dean of Faculty of Medicine	Local (Dean)	-
221	Syria	AlShahbaa Hospital, Aleppo city	Head of Alshahbaa Private Hospital	Local (Hospital)	-

Wright NJ. *BMJ Open* 2019; 9:e030452. doi: 10.1136/bmjopen-2019-030452

222	Syria	Tishreen University Hospital	In Arabic	Local (Ethical)	-
223	Thailand	Bangkok Ramathibodi Hospital	Institutional Review Board	Local (Ethical)	07-61-17
224	Thailand	Chiangmai Jiraporn Khorana	Ethics Committee	Local (Ethical)	SUR-2561-05619
225	Thailand	Hatyai Hospital	Research Ethics Committee	Local (Ethical)	80/2561
226	Thailand	Khon Kaen University	Ethics Committee for Human Research	Local (Ethical)	HE611526
227	Thailand	National Institute of Child Health (QSNICH)	Research Ethics Review Committee	Local (Ethical)	61-065
228	Thailand	Siriraj Hospital	Institutional Review Board	Local (Ethical)	687/2561(EC2)
229	Thailand	Sisaket Hospital	Research Ethics Committee	Local (Ethical)	071/2561.
230	Tunisia	CHU Hedi Chaker Hospital	President of the Ethical Committee	Local (Ethical)	-
231	Tunisia	Fattouma Bourguiba Hospital	President of the Ethical Committee	Local (Ethical)	-
232	Turkey	Adana State Training Hospital	Head Physician	Local (Hospital)	60247264-799-41
233	Turkey	Amerikan Hospital, Istanbul	Ethics Committee Chairman	Local (Ethical)	2018.235.irbi.028.
234	Turkey	Bezmialem University School of Medicine	Hospital President	Local (Hospital)	54022451-050.05.04
235	Turkey	Biruni University Hospital	University Ethics Committee	Local (Ethical)	-
236	Turkey	Gazi University School of Medicine	University Dean	Local (Dean)	24074710-604.01.01-47
237	Turkey	Karadeniz technical University	Head Physician	Local (Hospital)	E.10031
238	Turkey	Koç University	Ethics Committee Chairman	Local (Ethical)	2018.235.irbi.028
239	Turkey	Muğla Education and Research Hospital	Chief of Staff	Local (Hospital)	2018.235.IRB1.028
240	Uzbekistan	Republican Perinatal Center	In local script	Local (Ethical)	000174
241	Uzbekistan	Republican Specialized Scientific Practical Medical Center of Pediatrics, Tashkent	In local script	Local (Ethical)	-
242	Zambia	University Teaching Hospital, Lusaka	ERES Converge I Institutional Review Board	Local (Ethical)	2018-Sept-001
**TOTAL MICs:** 217					
**High-Income Countries (HICs)**					
243	Australia	Children’s Hospital at Westmead, Sydney	Research Governance Officer and Human Research Ethics Committee	Local (Ethical)	LNR/18/SCHN/347
244	Australia	Gold Coast University Hospital	Human Research Ethics Committee	Local (Ethical)	HREC/18/QGC/47042
245	Australia	Monash Children’s Hospital, Victoria	Human Research Ethics Committees	Local (Ethical)	HREC/43418/MonH-2018-67965(v1)
246	Australia	The Royal Children’s Hospital, Melbourne	Murdoch Children’s Research Institute and Research Ethics and Governance Officer	Local (Ethical)	QA/50138/RCHM-2018
247	Austria	Medical University of Graz	Ethics Committee	Local (Ethical)	31-157 ex. 18/19
248	Austria	Medical University of Vienna, Spitalgasse	Ethics Committee	Local (Ethical)	2006/2018
249	Belgium	Queen Fabiola University Children's Hospital	Ethics Committee	Local (Ethical)	B406201837832
250	Brunei Darussalam	RIPAS Hospital, Bandar Seri Begawan	Medical and Health Research and Ethics Committee	Local (Ethical)	MHREC/MOH/2018/7
251	Canada	Ste-Justine Children’s Hospital	Scientific Research Committee and Research Ethics Board for Ethical Review	Local (Ethical)	2019-2158
252	Chile	San Juan de Dios Hospital	Ethics Committee for Scientific Investigation	Local (Ethical)	47/2018
253	Czech Republic	Fakultni Nemocnice BRNO	Ethics Committee	Local (Ethical)	13-130219/EK
254	Czech Republic	University Hospital Hradec Kralove	Ethics Committee	Local (Ethical)	201812 s17p
255	Czech Republic	University Hospital Motol	Ethics Committee	Local (Ethical)	-
256	England	Alder Hey Children’s Hospital	Governance and Quality Assurance Team	Local (Hospital)	5714
257	England	Birmingham Children’s Hospital	Clinical Audit Registration and Management System	Local (Hospital)	CARMS-30164
258	England	Bristol Royal Hospital for Children	Audit Governor	Local (Hospital)	-
259	England	Evelina Children's Hospital, Guy's & St.Thomas' NHS Trust	Audit Team	Local (Hospital)	8956

Wright NJ. *BMJ Open* 2019; 9:e030452. doi: 10.1136/bmjopen-2019-030452

260	England	John Radcliffe Hospital, Oxford	Clinical Effectiveness Committee	Local (Hospital)	5150
261	England	King’s College Hospital	Governance Manager	Local (Hospital)	CH011
262	England	Leeds General Infirmary	Clinical Director and Deputy Caldicott Guardian	Local (Hospital)	Globalpaedsurg
263	England	Leicester and Kettering Hospitals	Clinical Audit Facilitator	Local (Hospital)	9482e
264	England	Nottingham Hospital	Audit Department	Local (Hospital)	18-350C
265	England	Southampton General Hospital	Audit Department	Local (Hospital)	SEV/0049
266	England	St Georges Hospital	Audit Department	Local (Hospital)	AUD1000163
267	France	CHU Amiens	Ethical Review Board	National (Ethical)	18.105
268	France	CHU Amiens-Picardie, Amiens	Ethical Review Board	National (Ethical)	18.105
269	France	CHU Angers	Ethical Review Board	National (Ethical)	18.105
270	France	CHU APHM-Marseille	Ethical Review Board	National (Ethical)	18.105
271	France	CHU APHP-Bicêtre	Ethical Review Board	National (Ethical)	18.105
272	France	CHU APHP-Necker	Ethical Review Board	National (Ethical)	18.105
273	France	CHU APHP-Robert Debré	Ethical Review Board	National (Ethical)	18.105
274	France	CHU Bordeaux	Ethical Review Board	National (Ethical)	18.105
275	France	CHU Limoges	Ethical Review Board	National (Ethical)	18.105
276	France	CHU Poitiers	Ethical Review Board	National (Ethical)	18.105
277	France	CHU Rennes	Ethical Review Board	National (Ethical)	18.105
278	France	CHU Rouen	Ethical Review Board	National (Ethical)	18.105
279	France	CHU StEtienne	Ethical Review Board	National (Ethical)	18.105
280	France	CHU Toulouse	Ethical Review Board	National (Ethical)	18.105
281	Germany	Frankfurt University Hospital	Ethics Committee	Local (Ethical)	346/18
282	Germany	Hospital St. Barbara Elisabeth Halle	Ethics Committee	Local (Ethical)	-
283	Germany	Krankenhaus Barmherzige Brüder Regensburg	Ethics Committee	Local (Ethical)	19-1263-101
284	Germany	Kinderchirurgie Vivantes Neukölln, Berlin	Coordinator of Clinical Research and Academic Teaching	Local (Hospital)	-
285	Germany	Medical Faculty Otto-von-Guericke University Magdeburg	Executive Director of Ethics Committee	Local (Ethical)	151/18
286	Germany	Universitat zu Lubeck	Ethics Committee	Local (Ethical)	18-249
287	Italy	San Matteo Hospital	Secretary to the Head of Science Technology	Local (Hospital)	20180097159
288	Hungary	Albert Szent-Györgyi Clinical Centre, Szeged	Human Investigation Review Board	Local (Ethical)	4413
289	Lithuania	Children‘s Hospital, Affiliate of Vilnius University Hospital SK	Chairman	Local (Hospital)	18VSR-1735 158200-18/9-1061-562
290	Lithuania	Lithuanian University of Health Sciences	Chairman of Kaunas RBTEK	Local (Hospital)	-
291	New Zealand	Christchurch Hospital	Quality Co-ordinator Childs Health and Audit Group	Local (Hospital)	BE-2-82
292	New Zealand	Starship Children's Hospital, NZ	Southern health and Disability Ethics Committee	Local (Ethical)	18/STH/143
293	New Zealand	Waikato Hospital	Director of Quality and Patient Safety	Local (Hospital)	RD018104
294	New Zealand	Wellington Hospital	Operations Manager Child Health Service	Local (Hospital)	-
295	Poland	Department of Children’s Developmental Defects Surgery and Traumatology	Bioethical Commission	Local (Ethical)	KNW/0022/KB/204/I/18
296	Poland	Medical University of Gdańsk	Bioethical Committee for Research	Local (Ethical)	NKBBN/456/2018
297	Poland	Medical University of Silesia, Katowice	Bioethical Commission	Local (Ethical)	KNW/0022/KB/172/18
298	Poland	Warsaw Children’s Memorial Health Institute	Bioethical Commission and Ethical Committee Assembly	Local (Ethical)	20/KBE/2018
299	Poland	Wladyslaw Buskowski Children’s Hospital	Bioethical Commission	Local (Ethical)	44/2018
300	Poland	Wroclaw University	Bioethical Commission	Local (Ethical)	133/XV R/2017

Wright NJ. *BMJ Open* 2019; 9:e030452. doi: 10.1136/bmjopen-2019-030452

301	Qatar	Sidra Medecine, Doha	Institutional Review Board	Local (Ethical)	1808029580
302	Saudi Arabia	King Fahd Armed Forces Hospital	Director of the Research Center, Research and Ethics Committee	Local (Ethical)	REC 261
303	Saudi Arabia	King Saud University College of Medicine	Institutional Review Board	Local (Ethical)	E-18-3427
304	Scotland	Glasgow Children’s Hospital, NHS Greater Glasgow and Clyde	Caldicott Guardian	Local (Hospital)	-
305	Scotland	Royal Hospital for Sick Children	Associate Medical Director	Local (Hospital)	-
306	Singapore	KK Women’s and Children’s Hospital, Little India	Head of Department	Local (Departmental)	-
307	South Korea	Cheonnam National University Hospital, Dong-gu, Gwangju	Chonnam National University Hospital Biomedical Research Ethics Review Committee Chairperson	Local (Ethical)	-
308	South Korea	Samsung Medical Centre	Institutional Review Board	Local (Ethical)	2018-09-110
309	South Korea	Seoul National University Children’s Hospital	Institutional Review Board	Local (Ethical)	H-1810-050-977
310	South Korea	Seoul St. Marys Hospital	Institutional Review Board	Local (Ethical)	KC18OCGI0669
311	Spain	Barcelona Sant Joan de Déu Hospital	Secretary of CEIM Sant Joan de Déu	Local (Hospital)	-
312	Spain	Complejo Hospitalario Universitario de a Coruña (CHUAC)	Head of Paediatric Surgery Unit	Local (Departmental)	-
313	Spain	Hospital Clinico Universitario de Valladolid	Director Manager and CEIM of Valladoid Health Area	Local (Hospital)	-
314	Spain	Maternal Hospital Infantil de Badajoz	Secretary of the Ethical Investigation Committee of Badajoz Clinic	Local (Ethical)	-
315	Spain	Zaragoza Hospital Universitario Miguel	Favourable Report Project of Biomedical Research a Secretary of the CEIC Aragón (CEICA)	Local (Hospital)	-
316	Sweden	Lund Skåne University Hospital Pediatric Care Hospital	Consultation group for quality registers	Local (Hospital)	-
317	Sweden	Queen Silvia Children’s Hospital	Area Manager of QSCH and Head of operations or unit manager	Local (Hospital)	-
318	Sweden	Sachs Children’s Hospital	Patient Area Director	Local (Hospital)	-
319	Sweden	Uppsala University Children’s Hospital	Section Chief, Paediatric Surgery Clinician	Local (Departmental)	-
320	Switzerland	Inselspital/University Hospital, Bern	Kantonale Ethics Committee	Local (Ethical)	-
321	UAE	Danat Al Emarat Hospital	Head of Surgery	Local (Departmental)	-
322	Uruguay	University De La Republica Facultad de Medicina	Service Chief for the Neonatal Department	Local (Departmental)	-
323	Uruguay	Centro Hospitalario Pereira Rossell	Audit Team	Local (Hospital)	-
324	USA	Ann and Robert H. Lurie Children’s Hospital of Chicago	Institutional Review Board	Local (Ethical)	IRB 2019-2334
325	USA	Beaumont’s Children’s Hospital in Royal Oak	Institutional Review Board	Local (Ethical)	IRB 2018-402
326	USA	Children’s Hospital of Wisconsin	Institutional Review Board	Local (Ethical)	1352795-2
327	USA	Dartmouth-Hitchcock Medical Center	Committee for the Protection of Human Subjects	Local (Ethical)	STUDY00031666
328	USA	Le Bonheur Children’s Hospital Memphis	UTHSC Institutional Review Board	Local (Ethical)	18-06176-XP
329	USA	Nationwide Children’s Hospital	Institutional Review Board Office	Local (Ethical)	IRB18-01005
330	USA	New York Presbyterian Morgan Stanley Children’s Hospital	Institutional Review Board	Local (Ethical)	IRB-AAAS0645
331	USA	Oregon Health and Science University	Institutional Review Board	Local (Ethical)	STUDY00018665
332	USA	Phoenix Children’s Hospital	Institutional Review Board	Local (Ethical)	PCH IRB #18-100
333	USA	University of Miami	Institutional Review Board	Local (Ethical)	20181054
334	USA	University of Michigan	Co-chairs of Institutional Review Board MED	Local (Ethical)	HUM 00151299
335	USA	University of Texas Medical Branch	Institutional Review Board Vice Chairman	Local (Ethical)	IRB18-0318

Wright NJ. *BMJ Open* 2019; 9:e030452. doi: 10.1136/bmjopen-2019-030452

336	USA	UT Southwestern Medical Center Children's Hospital	Institutional Review Board	Local (Ethical)	STU 072018-058
337	USA	Yale New Haven Hospital	Institutional Review Board	Local (Ethical)	2000024339
**TOTAL HICs:** 95					
**OVERALL TOTAL: 337**					

Wright NJ. *BMJ Open* 2019; 9:e030452. doi: 10.1136/bmjopen-2019-030452
