# Supplementary material for: Management and outcomes of gastrointestinal congenital anomalies in low, middle and high income countries: protocol for a multicentre, international, prospective cohort study
Source: BMJ Open. 2019 Sep 3;9(8):e030452. doi: 10.1136/bmjopen-2019-030452 (PMC6731898; doi:10.1136/bmjopen-2019-030452)
Supplement: Author's manuscript [file bmjopen-2019-030452.draft_revisions.pdf]

BMJ Open is committed to open peer review. As part of this commitment we make the peer review history of every article we publish publicly available.

When an article is published we post the peer reviewers' comments and the authors' responses online. We also post the versions of the paper that were used during peer review. These are the versions that the peer review comments apply to.

The versions of the paper that follow are the versions that were submitted during the peer review process. They are not the versions of record or the final published versions. They should not be cited or distributed as the published version of this manuscript.

BMJ Open is an open access journal and the full, final, typeset and author-corrected version of record of the manuscript is available on our site with no access controls, subscription charges or pay-per-view fees (http://bmjopen.bmj.com).

If you have any questions on BMJ Open's open peer review process please email info.bmjopen@bmj.com

BMJ Open

Management and Outcomes of Congenital Anomalies in Low-, Middle- and High-Income Countries: Protocol for a Multi-Centre, International, Prospective Cohort Study

Journal:	*BMJ Open*
Manuscript ID	bmjopen-2019-030452
Article Type:	Protocol
Date Submitted by the Author:	14-Mar-2019
Complete List of Authors:	Wright, Naomi; King's College London, King's Centre for Global Health and Health Partnerships, School of Population Health and Environmental Sciences
Keywords:	PAEDIATRIC SURGERY, Paediatric anaesthesia < ANAESTHETICS, Paediatric intensive & critical care < INTENSIVE & CRITICAL CARE, NEONATOLOGY, PAEDIATRICS, Neonatal intensive & critical care < INTENSIVE & CRITICAL CARE

SCHOLARONE™
Manuscripts

Management and Outcomes of Congenital Anomalies in Low-, Middle- and High-Income Countries: Protocol for a Multi-Centre, International, Prospective Cohort Study

Global PaedSurg Research Collaboration

Correspondence to: Naomi J. Wright, King's Centre for Global Health and Health Partnerships, School of Population Health and Environmental Sciences, King's College London, Denmark Hill, SE5 9RJ, UK. Email: naomiwright@doctors.org.uk

For peer review only

ABSTRACT

Introduction

Congenital anomalies are the 5th leading cause of death in children under 5-years of age globally, contributing an estimated half a million deaths per year. Very limited literature exists from low- and middle-income countries (LMICs) where most of these deaths occur. The Global PaedSurg Research Collaboration aims to undertake the first multi-centre, international, prospective cohort study of a selection of common congenital anomalies, comparing management and outcomes between low-, middle- and high-income countries (HICs) globally.

Methods and Analysis

The Global PaedSurg Research Collaboration consists of surgeons, paediatricians, anaesthetists and allied healthcare professionals involved in the surgical care of children globally. Collaborators will prospectively collect observational data on consecutive patients presenting for the first time, with one of seven common congenital anomalies (oesophageal atresia, congenital diaphragmatic hernia, intestinal atresia, gastroschisis, exomphalos, anorectal malformation and Hirschsprung's disease).

Data collection will be for a minimum of one month from October 2018 to April 2019 with a 30-day post-primary intervention follow-up period. Anonymous data will be collected on patient demographics, clinical status, interventions and outcomes using REDCap. Collaborators will complete a survey regarding the resources and facilities for neonatal and paediatric surgery at their centre.

The primary outcome is all-cause in-hospital mortality. Secondary outcomes include the occurrence of post-operative complications. Chi-squared analysis will be used to compare mortality between LMICs and HICs. Multilevel, multivariate logistic regression analysis will be undertaken to identify patient level and hospital level factors affecting outcomes with adjustment for confounding factors.

Ethics and Dissemination

At the host centre this study is classified as an audit not requiring ethical approval. Participating collaborators must gain local approval in accordance to their institutional ethical regulations. All collaborators will be encouraged to present the results locally, nationally and internationally. The results will be submitted for open access publication in a peer reviewed journal.

Registration Details

This study has been registered with ClinicalTrials.Gov, identifier: NCT03666767. The registration is available to view via: https://goo.gl/ffXNMH

Strengths and Limitations of this Study

- This will be the first large-series, geographically comprehensive, multi-centre, international, prospective cohort study to define the management and outcomes of a selection of common congenital anomalies in low-, middle- and high-income countries across the globe.
- The collaborative approach for this study allows a large series of high-quality data to be collected in a timely manner without overburdening high-volume, low-resource centres.
- The seven study conditions constitute a selection of the commonest life-threatening congenital anomalies requiring emergency surgical care in the neonatal period **(Box 1)**.
- We recognise that some children may not reach a facility capable of providing acute paediatric surgical care and hence the results obtained may be an underestimation of true morbidity and mortality, especially in LMICs.
- This study will only report on acute management and outcomes as follow up will be limited to 30-days post-primary intervention.

For peer review only

INTRODUCTION

In 2015, the Global Burden of Disease study concluded congenital anomalies to be the 5th leading cause of death in children under 5-years of age globally.[1] This equates to approximately half a million deaths from congenital anomalies each year, 97% of which occur in low- and middle-income countries (LMICs) where the incidence and prevalence is higher compared to high-income countries (HICs).[2,3] Indeed, this is likely to be an underestimation of the actual number of deaths due to under-diagnosis of neonates with congenital anomalies who die in the community and a lack of death certification in many LMICs.[4] There is limited research and a lack of congenital anomaly registries in LMICs and hence they have received very little global attention.[5]

The conditions forming the focus of this study **(Box 1)** constitute a selection of the most common life-threatening congenital anomalies, which involve the gastrointestinal tract. They each have an incidence of 1/2000 – 1/5000, they collectively form up to 40% of emergency neonatal surgery and associated mortality can be in excess of 50% in many LMICs.[6-9] Disparities in outcomes globally can be stark; for example the mortality from gastroschisis is 75-100% in many LMICs compared to 4% or less in HICs.[12-14] Reasons for poor outcomes include a lack of antenatal diagnosis, delayed presentation, limited neonatal transport and in-hospital resources, a dearth of trained support personnel and a lack of intensive care and parenteral nutrition for neonates.[9,15,16] In Uganda, it was calculated that only 3.5% of the need for neonatal surgery was met by the healthcare system.[8]

Box 1. Congenital Anomalies in the Global PaedSurg Study

- Oesophageal atresia (OA) +/- tracheo-oesophageal fistula (TOF)
- Congenital diaphragmatic hernia (CDH)
- Intestinal atresia (IA)
- Gastroschisis
- Exomphalos
- Anorectal malformation (ARM)
- Hirschsprung's disease

In 2010, the World Health Assembly passed a resolution recommending 'prevention whenever possible, to implement screening programmes and to provide care and ongoing support to children with birth defects and their families'.[2] Prevention is paramount, however this is not yet possible for many congenital anomalies and hence a focus on improving postnatal care and outcomes is vital. The Sustainable Development Goal 3.2 aims to end preventable deaths of newborns and children under the age of 5-years by 2030.[6, 10,11] With a third of infant deaths being attributed to congenital anomalies, clearly this will not be achievable without an accelerated effort towards the provision of surgical care for children. It is estimated that two-thirds of deaths and disability from congenital anomalies can be avoided with the provision of neonatal and paediatric surgical care.[6] Indeed, studies have demonstrated such provision can be highly cost-effective in terms of disability adjusted life years saved.[5] Yet neonatal and paediatric surgical care remain a low priority on the global health agenda.[5]

A shift is needed to focus on the provision of surgical care for children within National Health Plans and International Organisations and to elevate congenital anomalies on the global health agenda. This large-scale, geographically comprehensive, multi-centre prospective cohort study aims to define the current management and outcomes of a selection of common congenital anomalies globally and identify factors affecting outcomes that can be modified to improve care. This is vital to aid advocacy and global health prioritisation and inform future interventional studies aimed at improving outcomes.

AIM

To undertake the first large-scale, geographically comprehensive multi-centre, prospective cohort study comparing the management and outcomes of a selection of common congenital anomalies in low-, middle-, and high-income countries across the globe.

For peer review only - http://bmjopen.bmj.com/site/about/guidelines.xhtml

OBJECTIVES

1. To compare the mortality and post-intervention complications of a selection of common congenital anomalies in LMICs and HICs globally.

2. To identify patient level and hospital level factors affecting outcomes that be modified to improve care.

3. To establish a research collaboration consisting of children's surgical care providers across the world to help enhance research capacity and to create a platform for ongoing collaborative research and intervention studies aimed at improving outcomes.

4. To raise awareness and provide advocacy for neonatal and paediatric surgical care within global health prioritisation, planning, policy and funding.

For peer review only

METHODS AND ANALYSIS

Study Design

This is an international, multi-centre, prospective observational cohort study. The Global PaedSurg Research Collaboration consisting of children's surgical care providers (collaborators) across the world was established from November 2017 to facilitate data collection and continues to grow. Collaborators are free to choose one or more months between 1st October 2018 to the 30th April 2019 (inclusive) to recruit consecutive patients to the study, with a 30-day post-primary intervention follow up period.

Collaborators

International collaborators will have a variety of roles and responsibilities within the study. Local collaborators will establish mini-teams locally, gain study approval, utilise the protocol criteria to appropriately identify patients for study inclusion, collect prospective data and upload it to REDCap. Each hospital will have a local study lead who will hold overall responsibility for ensuring the data is accurate, complete and without duplications. Country-lead collaborators will help to recruit other collaborators from within their country and provide advice and support regarding gaining local study approval and data collection. They may also help with translation of the study literature to the local language if required. Continent and regional leads will help to recruit country leads, provide them with advice regarding the study and also encourage and co-ordinate presentations of the protocol at national and international meetings. Lead investigators will contribute to the study design through the provision of feedback from the pilot studies undertaken in multiple languages. An organising committee will help to co-ordinate all study activities and a steering committee will provide guidance throughout.

There are a number of benefits for collaborators participating the study. Publishing journal(s) will be asked to make all collaborators PubMed citable co-authors. This is based on an equal partnership model described by the Lancet and is used by a number of national and international collaboratives.[17-21] All collaborators will be listed as an author on resulting presentations. Collaborators will have the opportunity to present the study locally, nationally and internationally, initially the study protocol and later the results. This often provides collaborators, especially those who are junior or from LMICs, the opportunity to apply for funding to attend, present and network at such meetings. Participation in the study provides an easy route and insight into clinical research, which can be further established through participation in the 2-year Research Training Fellowship which is running alongside the main study free of charge for all interested collaborators.

Sample Selection

Collaborator and Hospital Inclusion Criteria:

For peer review only - http://bmjopen.bmj.com/site/about/guidelines.xhtml

All hospitals and healthcare professionals providing surgical care for neonates and children, presenting for the first time, with one or more of the study conditions can be included in the study. Collaborators should gain permission from the senior surgeon or physician who oversees the care of the children to be included in the study in order to participate. There can be up to three collaborators in a mini-team per month of data collection. One mini-team can collect data over one or more months or several mini-teams can collect data over a different month each. Each mini-team must contain at least one senior surgeon or physician to oversee the data collection process.

Patient Inclusion and Exclusion Criteria:

Any neonate, infant or child under the age of 16-years, presenting acutely for the first time, with one or more of the study conditions can be included in the study. Patients who have previously received surgery for their presenting condition or those representing with a complication of surgery are excluded. Patients presenting electively for surgery are excluded. Children who have received basic resuscitative care for their condition at a different healthcare facility and are then transferred to the study centre can be included. Children who only receive resuscitative treatment at the study centre and then are referred elsewhere for surgical care cannot be included since the outcome of the surgical care will not be known and also to avoid the risk of duplicate patients in the study. Patients who receive conservative treatment, palliative care, or no care must be included within the study to reflect true outcomes.

If a patient presents with more than one of the study conditions, the details of each condition that they present acutely with can be included, but not a previously managed condition. For example, a newborn presenting with oesophageal atresia and anorectal malformation would have both conditions included. A patient presenting for the first time with Hirschsprung's disease at several months of age who had a duodenal atresia repaired at birth would have the full details of the Hirschsprungs disease included, but the duodenal atresia would simply be noted as an associated anomaly.

Outcome Measures

The primary outcome is all-cause, in-hospital mortality.

For patient's hospitalised for over 30-days following primary intervention, a 30-day post-primary intervention mortality rate will be utilised. Those who do not receive a primary intervention, but remain alive and hospitalised at 30-days following primary admission, will have this time point used for recording their mortality status for the primary outcome. Primary outcome is defined in Table 1.

The secondary outcomes are the complications occurring within 30-days of primary intervention:

- Surgical site-infection
- Wound dehiscence
- Need for re-intervention
- Condition specific complications
- Condition specific outcome variables
- Length of hospital stay or time from admission to death in patients who do not survive
- 30-day post primary intervention mortality.

Secondary outcomes will not be collected on patients who do not receive a primary intervention within 30-days of hospital admission, with the exception of length of hospital stay or time from admission to death. 30-day follow-up will be undertaken within the capacity of the collaborating team; no additional funding will be provided.

Data Collection

Generic variables relating to the patient demographics, antenatal care, pre-hospital care, clinical condition, surgical intervention and outcomes will be collected for all patients in the study **(Table 1)**. Specific variables will be collected for each individual condition (**Supplementary File 1).**

For peer review only - http://bmjopen.bmj.com/site/about/guidelines.xhtml

Outcomes and variables have been chosen using published core outcome sets and commonly collected outcomes in systematic reviews and meta-analyses.[22-37] Collaborators will enter anonymous data via the secure internet-based Research Electronic Data Capture (REDCap) system. This will be stored on King's College London REDCap server. No individual collaborator, institution or country will be independently identifiable in the presented or published results.

A short survey will be completed by the local study lead and one other collaborating consultant or registrar on the resources and facilities available for neonatal and paediatric surgical care at their centre **(Supplementary File 2)**.

Table 1. Generic Data Points

Generic questions	Answers
During which month did the patient present to your hospital?	Please select the month that the patient presented to your hospital for the first time with this congenital anomaly. For example, if a baby was born with gastroschisis on the 29th September and presented to your hospital on the 1st October you should select October.
Has consent been provided to include this patient in the study?<br>If no, which condition did the patient present with?	Yes / No / Patient consent is not required for this study at my institution<br><br>Oesophageal atresia / Congenital diaphragmatic hernia / Intestinal atresia / Gastroschisis / Exomphalos / Omphalocele / Anorectal malformation / Hirschsprung's Disease.<br>Please select all the conditions that the patient presented with. Do not select a condition which the patient has already received surgical treatment for previously.
**Demographics**	
Gestational age at birth	Number of weeks from the first day of the women's last menstrual cycle until birth. Round up or down to the nearest week.
Age at presentation (in hours)	We understand this information may be difficult to obtain - please be as accurate as you can. Please round to the nearest hour. This number may be very large for patients who have a delayed presentation - please still enter it. For neonates born within your centre please enter 0. Enter unknown if unknown.
Gender	Male / Female/ Ambiguous/ Unknown
Weight at presentation	In kilograms (kg) on the day of presentation. Please provide a value to 1 decimal place.
Does the patient have another anomaly in addition to the study condition?	Yes, Cardiovascular, Yes, Respiratory, Yes: Gastrointestinal, Yes: Neurological, Yes: Genito-urinary, Yes: Musculoskeletal, Yes: Down syndrome, Yes: Beckwith-Wiedemann syndrome, Yes: Cystic fibrosis, Yes: Chromosomal, Yes: Other, No<br>Select all that apply. Include all anomalies diagnosed at any stage up until 30-days post primary intervention or 30-days following presentation for those who didn't receive an intervention. If you suspect an associated anomaly, but it has yet to be diagnosed, select 'other'.
Distance from the patient's home to your hospital	In kilometres (km). Please round to the nearest kilometre. Please enter 0 if born in your hospital.
**Antenatal Care and Delivery**	
Antenatal ultrasound undertaken?<br><br>If the condition was diagnosed antenatally, at what gestational age?	Yes: study condition diagnosed, Yes: problem identified but study condition not diagnosed, Yes: no problem identified, No<br>Please round up to the nearest week. If the patient has more than one study condition, please note the gestational age at which one or more of the conditions was first diagnosed.
Mode of transport to hospital?<br><br>Where did the patient present from? If other, please specify.	Ambulance, Other transport provided by the health service, Patient's own transport, Born within the hospital<br>Home / Community Clinic / General Practice / District Hospital / Other / Unknown<br>District hospital includes: secondary level healthcare, provincial hospital, general hospital, general mission hospital or regional hospital. It has general anaesthesia and can provide general surgical care.
Type of delivery:	Vaginal (spontaneous), Vaginal (induced), Caesarean section (elective), Caesarean section (urgent/non-elective), Unknown. Vaginal delivery includes those requiring forceps and ventouse.
**Clinical condition and patient care**	
Was the patient septic on arrival?<br><br>If yes, were appropriate antibiotics administered?	Yes, no<br>Sepsis is SIRS (Systemic Inflammatory Response Syndrome) with a suspected or confirmed bacterial, viral, or fungal cause. SIRS is a response to a stimulus, which results in two or more of the following: temperature > 38.5°C or < 36°C, tachycardia*, bradycardia* in children < 1 year old, tachypnoea*, leukopenia or leucocytosis*, hyperglycaemia*, altered mental status, hyperlactaemia*, increased central capillary refill time >2 seconds. *Variables are defined as values outside the normal range for age. Arrival is the time of birth for neonates born at your hospital.<br>Yes: within 1 hour of arrival, Yes: within the first day of arrival, No<br>Appropriate antibiotics are defined as either broad spectrum covering gram negative, gram positive and anaerobic bacteria OR antibiotics that are the standard empirical treatment for that condition according to local guidelines OR are based on sensitivities provided by a microbiology sample.
Was the patient hypovolaemic on arrival?	Yes/ No. Criteria for diagnosis include at least one of the following: prolonged central capillary refill time > 2 seconds, *tachycardia, mottled skin, *reduced urine output, cyanosis, impaired consciousness, *hypotension. *Variables are defined as values outside the normal range for age.

For peer review only - http://bmjopen.bmj.com/site/about/guidelines.xhtml

If yes, was an intravenous fluid bolus given?<br>If yes, how much intravenous fluid was given?	Yes: within 1 hour of arrival, Yes: on the first day of arrival, No<br>10 - 20mls/ kg, above 20mls/ kg<br>If less than 10mls/ kg was given please select 'no' for the question asking if intravenous fluid was given.
Was the patient hypothermic on arrival?<br><br>If yes, was the patient warmed on arrival to within a normal temperature range?	Yes/ No. Defined as < 36.5 degrees Celsius core temperature. Arrival is the time of birth for neonates born at your hospital.<br>Yes/ No. Only select yes if warming was commenced within 1 hour of arrival. Arrival is the time of birth for neonates born at your hospital.
Did the patient receive central venous access?<br><br><br><br>If yes, did the patient acquire central line sepsis?	Yes: umbilical catheter, Yes: peripherally inserted central catheter (PICC), Yes: percutaneously inserted central line with ultrasound guidance, Yes: surgically placed central line (open insertion), No.<br>Please select all that the patient received within 30-days of primary intervention or 30-days of presentation if no intervention was undertaken.<br>Yes: diagnosed clinically, Yes: confirmed on microbiology, No<br>Within 30-days of primary intervention or 30-days of presentation if no intervention was undertaken.
Time from arrival at your hospital to primary intervention in hours	(enter 0 if no intervention was undertaken)<br>**Primary intervention for each condition is defined as: Oesophageal atresia**; surgery, either temporising or definitive, to manage the oesophageal atresia and/ or tracheo-oesophageal fistula. **Congenital diaphragmatic hernia**; surgery to reduce the hernia and close the defect. **Intestinal atresia**; surgery, either temporising or definitive, to manage the obstruction including stoma formation and primary anastomosis. **Gastroschisis**; any procedure to either cover or reduce the bowel and/ or close the defect. This includes application of a silo (regardless of whether or not they go on to require surgery). It excludes initial covering of the bowel in a plastic covering (bag or cling film) prior to intervention. **Exomphalos**; surgery or application of topical treatment to the sac in patients managed conservatively (regardless of whether or not they go on to require surgery). **Hirschsprung's disease**; surgery, either temporising or definitive, or rectal/ distal bowel irrigation, laxatives or digital stimulation in patients managed conservatively. This does not include pre-operative washouts in patients planned to have surgery. **Anorectal malformation**; surgery, either temporising or definitive, or anal/ fistula dilatation in patients with a low anorectal malformation managed conservatively.
American Society of Anesthesiologists (ASA) Score at the time of primary intervention	1. Healthy person, 2. Mild systemic disease, 3. Severe systemic disease, 4. Severe systemic disease that is a constant threat to life, 5. A moribund patient who is not expected to survive without the operation, Not applicable - no intervention
What type of anaesthesia was used for the primary intervention?	General anaesthesia with endotracheal tube, General anaesthesia with laryngeal airway, Ketamine anaesthesia, Spinal/ caudal anaesthesia, Local anaesthesia only, No anaesthesia/ just analgesia, No anaesthesia/ no analgesia, Not applicable: no surgery or intervention undertaken.
Who undertook the anaesthetic for the primary intervention?	Anaesthetic doctor, Anaesthetic nurse, Medical officer, Surgeon, Other healthcare professional, No anaesthetic undertaken<br>If more than one of these personnel were present please select the most senior.
Who undertook the primary intervention?	Paediatric surgeon (or junior with paediatric surgeon assisting/ in the room), General surgeon (or junior with paediatric surgeon assisting/ in the room), Junior doctor, medical officer or other (without a paediatric or general surgeon assisting/ in the room), Trainee surgeon (without a paediatric or general surgeon assisting or in the room), Not applicable - no surgery or primary intervention undertaken.
Was a Surgical Safety Checklist used at the time of primary intervention?	Yes, No: but it was available, No: it was not available, Not applicable: a conservative primary intervention was undertaken, Not applicable: no surgery or primary intervention undertaken
Total duration of antibiotics following primary intervention	In days (including the day of surgery and the day antibiotics were stopped. Include intravenous and oral antibiotics).
Did the patient receive a blood transfusion?	Yes: not cross-matched, Yes: cross-matched, No: not required, No: it was required but not available. Within 30-days of primary intervention or 30-days of presentation if no intervention was undertaken.
Did the patient require ventilation?<br><br><br>If yes, for how long did the patient remain on ventilation?	Yes: and it was given, Yes: but it was not available, No<br>Within 30-days of primary intervention or 30-days of presentation if no intervention was undertaken. Please include all types of ventilation.<br>In days (include all days on ventilation within 30-days of primary intervention or 30-days of presentation if no intervention was undertaken).
Time to first enteral feed (post-primary intervention)	In days (include the day of primary intervention and the day of first enteral feed in the calculation). Enter 0 if enteral feeds were not commenced. Enter 999 if feeds were not stopped, for example in patients with Hirschsprung's Disease managed conservatively. Include all types of enteral feeding - oral, nasogastric, gastrostomy and other.
Time to full enteral feeds (post-primary intervention)	In days (enter 0 if the patient died before reaching full enteral feeds or 30 if the patient had not reached full enteral feeds at 30-days post primary intervention or 30-days following admission in patients who did not receive a primary intervention). Include all types of enteral feeding - oral, nasogastric, gastrostomy and other.
Did the patient require parenteral nutrition?<br><br>If yes, for how long did the patient receive parenteral nutrition?	Yes and it was given, Yes and it was sometimes available but less than required, Yes but it was not available, No<br>In days. Include all days that the patient received parenteral nutrition (any volume) up until 30-days post primary intervention or 30-days following presentation in patients who do not receive an intervention.
**Outcomes**	
Did the patient survive to discharge?	Yes/ No<br>Select yes if the patient was still alive in your hospital 30-days after primary intervention or 30-days after presentation in patients who do not receive a primary intervention.

For peer review only - http://bmjopen.bmj.com/site/about/guidelines.xhtml

If the patient was discharged prior, were they still alive at 30-days following primary intervention?<br><br>If no, cause of death?	Yes, No: not followed-up after discharge, Followed-up but not until 30-days post primary intervention<br>This can include all reliable communication with the patient/ patient's family including in person, via telephone and other.<br>Sepsis, Aspiration pneumonia, Respiratory failure, Cardiac failure, Malnutrition, Electrolyte disturbance, Haemorrhage, Lack of intravenous access, Hypoglycaemia, Recurrent tracheo-oesophageal fistula, Recurrent diaphragmatic hernia, Anastomotic leak, Ischaemic bowel, Ruptured exomphalos sac, Enterocolitis, Other. If other, please specify
Duration of hospital stay (days)	Please include the day of admission and the day of discharge in your calculation. For example, if a patient presented on 1st October and was discharged on the 5th October, their duration of hospital stay would be 5 days. If the patient died, please record the number of days from admission to death. Only include the duration of the primary admission, not subsequent admissions if the patient re-presented.
Did the patient have a surgical site infection?	Yes, No, Not applicable: no surgical wound<br>This is defined as one or more of the following within 30-days of surgery: 1) purulent drainage from the superficial or deep (fascia or muscle) incision, but not within the organ/ space component of the surgical site OR 2) at least two of: pain or tenderness; localised swelling; redness; heat; fever; AND the incision is opened deliberately to manage infection, spontaneously dehisces or the clinician diagnoses a SSI (negative culture swab excludes this criterion) OR 3) there is an abscess within the wound (clinically or radiologically detected).
Did the patient have a full thickness wound dehiscence?	Yes, No, Not applicable - no surgical wound.<br>This is defined as all layers of the wound opening within 30-days of surgery
Did the patient require a further unplanned intervention?	Yes - percutaneous intervention, Yes - surgical intervention, No, Not applicable - no primary intervention undertaken. Within 30-days of primary intervention. This does not include routine reduction and closure of the defect in neonates with gastroschisis receiving a preformed silo.
Was the patient followed up at 30-days post primary surgery or intervention to assess for complications?	Yes: reviewed in person, Yes: via telephone consultation, Yes: via other means, Yes: still an in-patient at 30-days, No: data is based on in-patient observations only, No: follow-up was done but prior to 30-days
If the patient had a complication, when was it diagnosed?	During the primary admission, As an emergency re-attender, At routine follow-up as an out-patient, Not applicable: no complications
What study condition does this patient have?	Oesophageal atresia, Congenital diaphragmatic hernia, Intestinal atresia, Gastroschisis, Exomphalos/ Omphalocele, Anorectal malformation, Hirschsprung's Disease<br>If the patient has presented for the first time with more than one of these conditions please select all that apply. If the patient presented on this occasion with one of these conditions, but previously had another condition managed then only select the condition they are presenting with on this occasion and enter that they have another anomaly in the demographics section above. For example, if the patient presents at 2-months with Hirschsprung's disease, but previously had a duodenal atresia repair please select Hirschsprung's disease here (not intestinal atresia) and tick in the section above that they have another gastrointestinal anomaly.

Data Quality

To ensure high quality of data, a detailed protocol for collaborators has been produced and published on the study website (www.globalpaedsurg.com) in 12 languages: English, French, Spanish, Portuguese, German, Italian, Chinese, Arabic, Korean, Lithuanian, Turkish and Russian. Clear and concise definitions have been provided for all data points on the protocol, on the data collection forms and within REDCap when entering the data. A study launch meeting was undertaken where the principal investigator presented the data collection process in detail, demonstrated use of REDCap and answered questions. This was recorded, circulated to all collaborators via email and placed on the website. A frequently asked questions document has been circulated via email and placed on the website. A two separate meetings were held by the principal investigator to detail the study, data collection process and answer questions amongst the country leads so they in turn can provide advice and support to local collaborators within their country. Again this was recorded, circulated and placed on the website.

A pilot study of the patient data collection form and institutional survey was undertaken by lead investigators to optimise the study design and to address any feasibility or other barriers to effective data collection and study completion across participating sites. The pilot study commenced on 1st August 2018 for 30 days in English, Spanish and French by 41 collaborator colleagues. The data collection forms were amended following feedback to clarify terminology, add important missing variables or descriptions and correct any translation errors. All translated data collection forms, REDCap and study documentation has been checked and verified by a native speaker for accuracy.

Data Validation

Ten percent of collaborating centres will be selected at random for data validation by an independent research collaborator. The aim will be to determine the numbers of patients eligible during the data

For peer review only - http://bmjopen.bmj.com/site/about/guidelines.xhtml

collection period to check if any were missed and collect a selection of data again to cross-check for accuracy. Validating questions have been built into the data collection tool. At least 90% of primary and secondary outcomes must be completed for each patient. All collaborators within validating centres will be asked to complete a brief survey regarding their experience with data collection to identify any potential areas for error and to aid with data interpretation.

Sample Size Calculation

A sample size calculation was undertaken using Stata/IC 15.0 based on Bonferroni correction for multiple testing, assuming 80% power and an overall type 1 error of 5%. The required sample size for each condition has been calculated for the primary outcome of mortality in LMICs compared to HICs and also low, middle and high-income countries separately **(Table 2).** Mortality estimations are based on pooled data from published studies on these conditions in low-, middle- and high-income countries respectively.

Based on the patient numbers included in the previously undertaken PaedSurg Africa study, which utilised a similar study design, the estimated sample sizes to detect a significant difference between LMICs and HICs in this study are achievable.[13]

Table 2. Estimated mortality and sample sizes for low, middle and high-income countries and the mean number of cases per month per institution globally

Condition	Mortality LIC (%, n)	Mortality MIC (%, n)	Mortality LMIC combined (%, n)	Mortality HIC (%, n)	Sample size for LIC	Sample size for MIC	Sample size for HIC	Sample size for LMIC vs HIC (per group)	Mean no. cases/ month/ institution (L,M&HIC combined)
OA +/- TOF	79.5% (62/78)	41.8% (623/1488)	43.7% (685/1566)	2.7% (6/221)	34	34	23	21	1.02
CDH	-	47.4% (130/274)	47.4% (130/274)	20.4% (201/982)	-	-	-	63	0.54
IA	42.9% (42/98)	40.0% (97/241)	41.0% (139/339)	2.9% (12/407)	6014	6014	25	24	0.63
Gastroschisis	83.1% (211/254)	42.6% (205/481)	56.6% (416/735)	3.7% (28/748)	29	29	24	15	0.85
Exomphalos	25.5% (41/161)	31.9% (132/414)	30.1% (173/575)	12.7% (40/316)	1040	1040	196	115	0.63
ARM	26.3% (26/99)	17.5% (243/1391)	18.1% (269/1490)	3% (14/462)	460	460	90	85	1.34
Hirschsprung's Disease	19.1% (33/173)	16.8% (55/328)	17.6% (88/501)	2.3% (43/1897)	5802	5802	85	79	2.21

Estimated Study Population

The mean number of cases presenting to an institution per month for each study condition was estimated from published studies across all income settings **(Table 2)**. On average most institutions caring for patients with these conditions receive 1-2 new cases per month; each participating institution would expect approximately 7-14 new cases in the study per month although this can vary. The aim is to include a minimum of 365 months of data; 183 months from LMICs and 183 months from HICs. This should ensure enough cases of exomphalos to determine a significant difference between LMICs and HICs; fewer months of data are required to determine significant differences between other study conditions. An up-to-date total of patient numbers within the study will be maintained on the study website.

Data Analysis

Patient and Institutional Data:

Data will be analysed using Stata and SAS 9.4 (Cary, NC; USA). Missing data for the covariates will be analysed to determine whether it is related to the outcome and either complete-case analyses or multiple imputation techniques will be used for analyses accordingly.

For peer review only - http://bmjopen.bmj.com/site/about/guidelines.xhtml

Significant differences in mortality between LMICs and HICs will be determined for each of the study conditions using Chi-squared analysis, or Fischer's exact test if either group contains less than 10 patients. World Bank classification of low-, middle- and high-income countries during the fiscal year 2018 will be used.[38]

Univariate logistic regression analyses will be conducted between covariates and the primary outcome of mortality. Based on the results, covariates with a p-value of <0.10 will be included in the multivariate model. The final multi-level multivariate logistic model will be determined using stepwise backward elimination to interventions and peri-operative factors affecting outcomes. Data will be adjusted for confounding factors and effect modifiers. Potential confounders include: gestation age at birth, weight, time from birth to presentation and ASA score at the time of primary intervention. Potential effect modifiers include: administration of peri-operative antibiotics, fluid resuscitation, thermal control and provision of other condition specific neonatal care such as parenteral nutrition in neonates with gastroschisis.

Multi-level multivariate logistic regression analysis will also be undertaken to identify institutional factors affecting mortality with adjustment for confounders. P< 0.05 will be deemed significant.

Data Validation:

A weighted kappa statistic will be utilised to determine the level of agreement between the patient data in the main study and validation data. A weighted kappa statistic will be also utilised to determine the level of agreement between institutional surveys independently completed by the local study lead and one other consultant or registrar at each participating centre. Results will be presented as a proportion of agreement for each variable being validated.

Patient and Public Involvement

CDH UK, a patient and family advisory group and charity, provided input into the design of the study protocol and data collection tool. Their input will be sought on the findings and dissemination of the results.

ETHICS AND DISSEMINATION

Research Ethics Approval

The study has been classified as an audit at the host institution and hence did not require ethical approval. The study fulfils the audit criteria as follows: 1) All data collected measures current practice. The study does not involve any changes to patient management; 2) Current practice and outcomes in low, middle and high-income countries will be compared to published standards in the literature. **Table 2** details the current mortality standards for each of the seven study conditions in high-income countries; 3) All the study data is routinely collected information which should be known to the study team without asking additional questions to the patients/parents; 4) All data to be entered into REDCap is entirely anonymous; 5) No individual patient, collaborator, institution or country will be independently identifiable in the study results; 6) All data will be stored securely and will be governed by King's College London data protection team. Research collaborators will be required to gain approval for the study at their institution according to local ethical regulations.

Study Dissemination

The study concept and design will be presented at international conferences in order to recruit collaborators. Following completion, the results will be presented at local, national and international conferences globally. Both the promotional presentations of the study protocol and the study results will be presented by study collaborators of all levels of training, disciplines and regions of the world. The results will be submitted for open access publication in a peer reviewed journal. Following publication, the full anonymous, de-identified dataset will be made publicly available via an online repository. Collaborators will have the opportunity to undertake sub-analyses of the data for their country (if all collaborators from that country agree), region or continent.

For peer review only - http://bmjopen.bmj.com/site/about/guidelines.xhtml

For peer review only

DISCUSSION

This study aims to define, for the first time, the management and outcomes of a selection of common life-threatening congenital anomalies across the globe. This will help to raise awareness of the unacceptable disparities in outcomes between low-, middle- and high-income countries and the need to focus on improving access to quality surgical care for neonates with congenital anomalies within national health plans and global health prioritisation. It is hoped that factors affecting mortality and morbidity will be identified that can be modified to improve care. Establishment of the Global PaedSurg Research Collaboration developed during this study will create a platform for ongoing collaborative work and interventional studies aimed at improving outcomes in the future.

For peer review only

For peer review only - http://bmjopen.bmj.com/site/about/guidelines.xhtml

ADDITIONAL INFORMATION

Twitter: @GlobalPaedSurg

Website: http://globalpaedsurg.com

Author Contributions: The principal investigator conceived the idea for the study, gained study funding, wrote the study protocol, designed the data collection tools, established the study team, co-ordinated the pilot study, revised the study design/ data collection tools following feedback and made critical revisions to the manuscript for publication. The steering committee contributed critical input and revisions to the funding application, study design, protocol and manuscript for publication. The writing committee drafted the protocol manuscript for publication and contributed as organising committee members. The organising committee assisted in the recruitment of and communication with collaborators to participate in the pilot study, helped to co-ordinate the pilot study and summarise the feedback, made critical revisions to the data collection tools in multiple languages and contributed to the study design. The lead investigators contributed to the study design and content of the data collection forms through feedback following participation in the pilot study. All contributed to the content of this manuscript.

Funding Statement: Naomi Wright, Principal Investigator, is funded by the Wellcome Trust through a Clinical PhD in Global Health undertaken at King's College London (Funder Reference: 203905/Z/16/Z). The Wellcome Trust had no input into the study protocol other than to recommend open-access publication in a peer-reviewed journal and to make the anonymised dataset publicly available. Nick Sevdalis' (NS) research is supported by the National Institute for Health Research (NIHR) Collaboration for Leadership in Applied Health Research and Care South London at King's College Hospital NHS Foundation Trust. NS is a member of King's Improvement Science, which is part of the NIHR CLAHRC South London and comprises a specialist team of improvement scientists and senior researchers based at King's College London. Its work is funded by King's Health Partners (Guy's and St Thomas' NHS Foundation Trust, King's College Hospital NHS Foundation Trust, King's College London and South London and Maudsley NHS Foundation Trust), Guy's and St Thomas' Charity, the Maudsley Charity and the Health Foundation. NS and Andrew Leather are also supported by the NIHR Global Health Research Unit on Health System Strengthening in Sub-Saharan Africa, King's College London (GHRU 16/136/54) and by the ASPIRES research programme in LMICs (Antibiotic use across Surgical Pathways - Investigating, Redesigning and Evaluating Systems), funded by the Economic and Social Research Council. The views expressed are those of the authors and not necessarily those of the NHS, the NIHR or the Department of Health and Social Care.

Competing Interest's Statement: Nick Sevdalis is the director of the London Safety and Training Solutions Ltd, which offers training in patient safety, implementation solutions and human factors to healthcare organisations. No other conflicts of interest are declared.

Patient Consent: Collaborators must follow their local ethical guidelines regarding patient consent.

Ethics Approval: This study has been classified as a clinical audit with written confirmation from King's College London Ethics Committee that it does not therefore require ethical approval. All participating centres must gain local study approval to participate according to their institutional ethical regulations.

Provenance and Peer Review: Not commissioned; externally peer reviewed.

Open Access: This is an Open Access article distributed in accordance with the terms of the Creative Commons Attribution (CC BY 4.0) license, which permits others to distribute, remix, adapt and build upon this work, for commercial use, provided the original work is properly cited.

Licence Statement: I, the Submitting Author has the right to grant and does grant on behalf of all authors of the Work (as defined in the below author licence), an exclusive licence and/or a non-exclusive licence for contributions from authors who are: i) UK Crown employees; ii) where BMJ has

For peer review only

agreed a CC-BY licence shall apply, and/or iii) in accordance with the terms applicable for US Federal Government officers or employees acting as part of their official duties; on a worldwide, perpetual, irrevocable, royalty-free basis to BMJ Publishing Group Ltd ("BMJ") its licensees and where the relevant Journal is co-owned by BMJ to the co-owners of the Journal, to publish the Work in BMJ Open and any other BMJ products and to exploit all rights, as set out in our licence.

The Submitting Author accepts and understands that any supply made under these terms is made by BMJ to the Submitting Author unless you are acting as an employee on behalf of your employer or a postgraduate student of an affiliated institution which is paying any applicable article publishing charge ("APC") for Open Access articles. Where the Submitting Author wishes to make the Work available on an Open Access basis (and intends to pay the relevant APC), the terms of reuse of such Open Access shall be governed by a Creative Commons licence – details of these licences and which Creative Commons licence will apply to this Work are set out in our licence referred to above.

Acknowledgements: Thank you to Bolaji Coker for the REDCap administration and management. Thank you to Beverley Power (CDH UK) for representing patients, parents and families through your feedback on the study design during the pilot study. Thank you to Xiya Ma and Dylan Goh for helping with the Chinese translation of study documentation.

COLLABORATORS

Principal Investigator: Naomi Wright (King's College London, UK)

Steering Committee: Niyi Ade-Ajayi (King's College Hospital, UK), Adesoji Ademuyiwa (Lagos University Teaching Hospital, Nigeria), Emmanuel Ameh (National Hospital, Abuja, Nigeria), Justine Davies (University of Birmingham, UK), Kokila Lakhoo (University of Oxford and Oxford University Hospitals, UK), Dan Poenaru (McGill University, Montreal, Canada), Nick Sevdalis (King's College London, UK), Emily Smith (Baylor University, Texas, USA), Andy Leather (King's College London, UK)

Writing Committee: Harmony Ubhi (King's College London, UK), Samuel Parker (Imperial College London, UK), Godfrey Sama Philipo (Muhimbili University of Health and Allied Sciences, Tanzania)

Organising Committee: Sadi Abukhalaf (Al Quds University, Palestine), Nana Adofo-Ansong (Mahikeng Provincial Hospital, South Africa), Melika Akhbari (King's College London, UK), Ahmad Alhamid (University of Aleppo, Syria), Osaid H. Alser (University of Oxford, UK), Emrah Aydin (Koc University, Turkey), Yousra-Imane Benaskeur (Universite de Montreal, Canada), Shrouk M. Elghazaly (Assiut University, Egypt), Safa abdal Elrais (University of Tripoli, Libya), Sophia Hashim (University College London, UK), Laura Herrera (Geisel School of Medicine, Dartmouth, USA), Gabriella Hyman (University of Witwatersrand, South Africa), Henang Kwasau (College of Medicine and Allied Health Sciences, University of Sierra Leone), Yang Liu (Children's Hospital, Zhejiang University School of Medicine, China), Bruno Martinez-Leo (Moctezuma Children's Hospital, Mexico), Kelly Naranjo (Columbia University Medical Centre, USA), Ibrahim Nour (Jordan University Hospital, Jordan), Cristiana Riboni (University of Pavia, Italy), Mahmoud Saleh (University of Gezira, Sudan), Hosni Khairy Salem (Cairo University, Egypt), Patricia Shinondo (University Teaching Hospital, Lusaka, Zambia), Marcus Sim (Stepping Hill Hospital, UK), Hannah Thompson (King's College Hospital, UK), Agota Vaitkiene (Vilnius University Hospital Santaros Kliniko, Lithuania), Dominique Vervoort (Harvard Medical School, USA), Isabelle Williams (Cambridge University, UK), Aayenah Yunus (King's College London, UK).

Lead Investigators: Muhammad Amjad Chaudhary, Adnan Ahmed Khan Khattak, Muhammad Bin Amjad (Children's Hospital, PIMS, Islamabad, Pakistan), Marlene Dominguez Anaya (Children's Hospital Manuel A.Villarroel Cochabamba, Bolivia), Samiul Hasan, Sabbir Karim, Ashrarur Rahman Mitul (Dhaka Shishu (Children) Hospital, Bangladesh), Paolo Bragagnini, Segundo Rite (Hospital Universitario Miguel Servent, Zaragoza), Hana Arbab, Lubna Samad, Aqil Soomro (Indus Hospital, Pakistan), Niveshni Maistry (John Radcliffe Hospital, UK), Raed Nael Al-Taher, Ibrahim Rabi Nour, Osama Abdul Kareem Sarhan (Jordan University Hospital, Jordan), Muhammad Arshad, Taimur

Qureshi, Hina Yousaf (Liaquat National Hospital, Pakistan), Candy SC Choo, Doris Mae Dimatatac, Shireen Anne Nah (KK Women's and Children's Hospital, Singapore), Vijay Anand Ismavel, Ann Miriam, Shajin T (Makunda Christian Leprosy and General Hospital, India), Monica Ivanov, Andreea Serban (Marie Curie Hospital in Bucharest, Romania), Bruno Martinez-Leo (Moctezuma Children's Hospital, Mexico), Eva Blazquez-Gomez, Luis Garcia-Aparicio, Martí Iriondo, Jordi Prat, Xavier Tarrado (Hospital Sant Joan de Deu, Spain), Lars Hagander, Emma Svensson (Skane University Hospital's Pediatric Care Hospital, Lund, Sweden), Alhassan Abdul-Mumin, Dominic Bagbio, Sheila Owusu, Stephen Tabiri (Tamale Teaching Hospital, Ghana), Dayang Anita Abdul Aziz (UKM Medical Centre, Kuala Lumpur, Malaysia).

For peer review only

REFERENCES

1. GBD Child Mortality Collaborators. Global, regional, national, and selected subnational levels of stillbirths, neonatal, infant, and under-5 mortality, 1980-2015: a systematic analysis for the Global Burden of Disease Study 2015. Lancet 2016; 388(10053): 1725-74.
2. Flores A, Valencia D, Sekkarie A, et al. Building capacity for birth defects surveillance in Africa: Implementation of an intermediate birth defects surveillance workshop. J Glob Health Perspect 2015; 2015.
3. Sitkin NA, Ozgediz D, Donkor P, Farmer DL. Congenital anomalies in low- and middle-income countries: the unborn child of global surgery. World J Surg 2015; 39(1): 36-40.
4. Boyle B, Addor MC, Arriola L, et al. Estimating Global Burden of Disease due to congenital anomaly: an analysis of European data. Arch Dis Child Fetal Neonatal Ed 2017.
5. Ozgediz D, Langer M, Kisa P, Poenaru D. Pediatric surgery as an essential component of global child health. Semin Pediatr Surg 2016; 25(1): 3-9.
6. Farmer D, Sitkin N, Lofberg K, Donkor P, Ozgediz D. Surgical Interventions for Congenital Anomalies. In: Debas HT, Donkor P, Gawande A, Jamison DT, Kruk ME, Mock CN, eds. Essential Surgery: Disease Control Priorities, Third Edition (Volume 1). Washington (DC); 2015.
7. Sakonidou S, Ali K, Farmer I, Hickey A, Greenough A. Mortality and short-term morbidity in infants with exomphalos. Pediatr Int 2018.
8. Ameh EA, Seyi-Olajide JO, Sholadoye TT. Neonatal surgical care: a review of the burden, progress and challenges in sub-Saharan Africa. Paediatr Int Child Health 2015; 35(3): 243-51.
9. Ekenze SO, Ajuzieogu OV, Nwomeh BC. Challenges of management and outcome of neonatal surgery in Africa: a systematic review. Pediatr Surg Int 2016; 32(3): 291-9.
10. United Nations. Sustainable Development Goals. 2015. http://www.un.org/sustainabledevelopment/health/ (accessed 27th February 2018).
11. Wright NJ, Anderson JE, Ozgediz D, Farmer DL, Banu T. Addressing paediatric surgical care on World Birth Defects Day. Lancet 2018; 391(10125): 1019.
12. Wright NJ, Zani A, Ade-Ajayi N. Epidemiology, management and outcome of gastroschisis in Sub-Saharan Africa: Results of an international survey. Afr J Paediatr Surg 2015; 12(1): 1-6.
13. PaedSurg Africa Research Collaboration. Paediatric Surgery across Sub-Saharan Africa: A Multi-Centre Prospective Cohort Study. https://clinicaltrials.gov/ct2/show/NCT03185637 (Accessed 26th January 2019).
14. Bradnock T, Marven S, Owen A, et al. Gastroschisis: one year outcomes from national cohort study. BMJ 2011; 343(d6749).
15. Krishnaswami S, Nwomeh BC, Ameh AE. The pediatric surgery workforce in low- and middle-income countries: problems and priorities. Semin Pediatr Surg 2016; 25(1): 32-42.
16. Okoye MT, Ameh EA, Kushner AL, Nwomeh BC. A pilot survey of pediatric surgical capacity in West Africa. World J Surg 2015; 39(3): 669-76.
17. GlobalSurg Collaborative. Mortality of emergency abdominal surgery in high-, middle- and low-income countries. Br J Surg 2016; 103(8): 971-88
18. GlobalSurg Collaborative. Surgical site infection after gastrointestinal surgery in high-income, middle-income, and low-income countries: a prospective, international, multi-centre cohort study. Lancet Infect Dis 2018; 18(5): 516-25.
19. GlobalSurg Collaborative. Laparoscopy in management of appendicitis in high-, middle-, and low-income countries: a multicenter, prospective, cohort study. Surg Endosc 2018.
20. GlobalSurg Collaborative. Determinants of morbidity and mortality following emergency abdominal surgery in children in low-income and middle-income countries. BMJ Glob Health 2016; 1(4): e000091.
21. Bhangu A, Kolias AG, Pinkney T, Hall NJ, Fitzgerald JE. Surgical research collaboratives in the UK. Lancet 2013; 382(9898): 1091-2.
22. Long AM, Bunch KJ, Knight M, Kurinczuk JJ, Losty PD, Baps C. Early population-based outcomes of infants born with congenital diaphragmatic hernia. Arch Dis Child Fetal Neonatal Ed 2018.
23. Ruttenstock E, Wright N, Barrena S, et al. Best oxygenation index on day 1: a reliable marker for outcome and survival in infants with congenital diaphragmatic hernia. Eur J Pediatr Surg 2015; 25(1): 3-8.
24. Burjonrappa S, Crete E, Bouchard S. Comparative outcomes in intestinal atresia: a clinical outcome and pathophysiology analysis. Pediatr Surg Int 2011; 27(4): 437-42.
25. Forrester MB, Merz RD. Structural birth defects associated with omphalocele and gastroschisis, Hawaii, 1986-2001. Congenit Anom (Kyoto) 2008; 48(2): 87-91.

For peer review only - http://bmjopen.bmj.com/site/about/guidelines.xhtml

26. Kunz SN, Tieder JS, Whitlock KJ, Jackson C, Avansino JR. Primary fascial closure versus staged closure with silo in patients with gastroschisis: a meta-analysis. J Pediatr Surg 2013; 48(4): 845-57.
27. Ross AR, Eaton S, Zani A, Ade-Ajayi N, Pierro A, Hall NJ. The role of preformed silos in the management of infants with gastroschisis: a systematic review and meta-analysis. Pediatr Surg Int 2015; 31(5): 473-83.
28. Levitt MA, Pena A. Anorectal malformations. Orphanet J Rare Dis 2007; 2: 33.
29. Van der Steeg HJ, Schmiedeke E, Bagolan P, et al. European consensus meeting of ARM-Net members concerning diagnosis and early management of newborns with anorectal malformations. Tech Coloproctol 2015; 19(3): 181-5.
30. Holschneider A, Hutson J, Pena A, et al. Preliminary report on the International Conference for the Development of Standards for the Treatment of Anorectal Malformations. J Pediatr Surg 2005; 40(10): 1521-6.
31. Bradnock TJ, Knight M, Kenny S, Nair M, Walker GM, British Association of Paediatric Surgeons Congenital Anomalies Surveillance S. Hirschsprung's disease in the UK and Ireland: incidence and anomalies. Arch Dis Child 2017; 102(8): 722-7.
32. Ross AR, Hall NJ. Outcome reporting in randomized controlled trials and systematic reviews of gastroschisis treatment: a systematic review. J Pediatr Surg 2016; 51(8): 1385-9.
33. Allin BSR, Hall NJ, Ross AR, et al. Development of a gastroschisis core outcome set. Arch Dis Child Fetal Neonatal Ed 2018.
34. Allin BS, Irvine A, Patni N, Knight M. Variability of outcome reporting in Hirschsprung's Disease and gastroschisis: a systematic review. Sci Rep 2016; 6: 38969.
35. Watanabe S, Suzuki T, Hara F, Yasui T, Uga N, Naoe A. Omphalocele and Gastroschisis in Newborns: Over 16 Years of Experience from a Single Clinic. J Neonatal Surg 2017; 6(2): 27.
36. Tan KB, Tan KH, Chew SK, Yeo GS. Gastroschisis and omphalocele in Singapore: a ten-year series from 1993 to 2002. Singapore Med J 2008; 49(1): 31-6.
37. Schneider A, Blanc S, Bonnard A, et al. Results from the French National Esophageal Atresia register: one-year outcome. Orphanet J Rare Dis 2014; 9: 206.
38. World Bank. World Bank Country and Lending Groups. 2018. https://datahelpdesk.worldbank.org/knowledgebase/articles/906519-world-bank-country-and-lending-groups (Accessed 26th January 2019).

For peer review only

Management and Outcomes of Congenital Anomalies in Low-, Middle- and High-Income Countries: Protocol for a Multi-centre, International, Prospective Cohort Study

SUPPLEMENTARY FILE 1: Condition Specific Data Points

Oesophageal Atresia (OA) +/- Tracheo-Oesophageal Fistula (TOF)

Question	Answers
Type of OA +/- TOF (Gross classification)	A, B, C, D, E<br>A: without a fistula, B: proximal TOF with distal OA, C: distal TOF with proximal OA, D: proximal and distal TOF, E: H-type TOF without OA.
Long or short gap?	Long, Short, Unknown<br>Long gap OA: A gap of 4 vertebral bodies or more. Anatomically cases either have no TOF or a gap of over 4 vertebral bodies following division of the distal fistula making primary repair unfeasible. Short OA: A gap of less than 4 vertebral bodies. Primary anastomosis typically feasible.
Pneumonia at presentation?	Yes: diagnosed clinically, Yes: diagnosed radiologically, Yes: other means of diagnosis, No: patient born in the study centre, No: patient born outside the study centre but no evidence of pneumonia on arrival<br>Pneumonia is defined as lung inflammation typically caused by bacterial or viral infection, in which the air sacs fill with pus and may become solid.
Primary intervention:<br><br><br>If the patient had a primary oesophageal anastomosis, was a post-operative oesophagogram undertaken?<br>If yes, routine or clinically indicated?<br>If yes, when?<br>If yes, what was the result?<br>For patients diagnosed with a leak radiologically, was it associated with clinical symptoms?<br>Time to first oral feed post-operatively<br><br><br><br>Time to full oral feeds<br><br><br><br>For patient's not receiving a primary oesophageal anastomosis, at what age is definitive surgery planned?<br>For patient's not receiving a primary oesophageal anastomosis, what is the future planned procedure?<br><br>If the patient had surgery, what was the approach?<br><br><br>If thoracoscopic or laparoscopic, was the surgery converted to open?<br>Did the patient have a condition specific complication within 30-days of primary intervention?<br><br>Did the patient have tracheomalacia?<br><br>If yes, was an intervention undertaken? If other, please specify	TOF ligation, Oesophageal anastomosis, Oesophagostomy, Gastrostomy, Ligation of the distal oesophagus, Gastro-oesophageal disconnection, Foker technique, Fundoplication, Other (please specify), Palliative care<br>Select all that apply.<br>Yes, No. (At any stage)<br><br>Routine, Clinically indicated<br>Number of days after primary surgery<br>Leak, No leak<br>Yes, No<br><br>In days. Please include the day of surgery and the first day of oral feeds in the calculation. Enter 0 if oral feeds were not commenced within 30-days of primary intervention. Do not include other types of enteral feeding such as nasogastric or gastrostomy feeding.<br>In days (enter 0 if the patient died before reaching full oral feeds or 30 if the patient had not reached full oral feeds at 30-days post primary intervention). Do not include other types of enteral feeding such as nasogastric or gastrostomy feeding.<br>In months (enter unknown if not planned or enter not applicable if primary anastomosis was undertaken).<br>Gap assessment, Primary oesophageal anastomosis if possible, Gastric pull-up, Jejunal interposition, Colonic interposition, Not applicable: primary anastomosis undertaken, Other, Unknown. Select all that apply. If other, please specify.<br>Thoracotomy muscle cutting, Thoracotomy muscle splitting, Thoracoscopy, Laparotomy, Laparoscopy, Limited local incision, Other.<br>During primary surgery. If other, please specify.<br>Yes, No<br><br>Pneumonia, Mediastinitis, Pneumothorax, Chylothorax, Haemothorax, Anastomotic leak, Anastomotic stricture, Recurrent TOF, Other, None<br>Select all that apply. If other, please specify.<br>Yes: diagnosed clinically, Yes: diagnosed on bronchoscopy, Yes: diagnosed on CT, Yes: diagnosed on bronchogram, Yes: other method of diagnosis, No<br>Yes: aortopexy, Yes: tracheostomy, Yes: tracheal stent, Yes: supportive management (oxygen +/- ventilation) only, Yes: other treatment, No

Congenital Diaphragmatic Hernia (CDH)

Question	Answers
Type of CDH.<br><br><br>Type of Bochdalek CDH (CDH Study Group Classification)	Left posteriolateral (Bochdalek), Right posteriolateral (Bochdalek), Bilateral posteriolateral (Bochdalek), Central, Anterior (Morgagni), Other. If other, please specify.<br>A, B, C, D, Other (specify), Unknown.<br>Defect A: smallest defect, usually "intramuscular" defect with >90% of the hemi-diaphragm present; this defect involves < 10% of the circumference of the chest wall. Defect B: 50-75% hemi-diaphragm present; this defect involves < 50% of

For peer review only - http://bmjopen.bmj.com/site/about/guidelines.xhtml

If bilateral, what was the type of Bochdalek hernia on the left?<br>If bilateral, what was the type of Bochdalek hernia on the right?	the chest wall. Defect C: < 50% hemi-diaphragm present; this defect involves >50% of the chest wall. Defect D: largest defect (previously known as "agenesis"); complete or near complete absence of the diaphragm with < 10% hemi-diaphragm present; this defect involves >90% of the chest wall. Surgically, it is an absent posterior rim beyond the spine, absent posterior-lateral rim, and an anterior/anterior-medial rim which is miniscule. As it is truly unusual to have zero tissue at all, this is the CDHSG member consensus. "D" defects should all require a patch (or muscle flap) for repair.<br>A, B, C, D, Other, Unknown<br>If other, please specify.<br>A, B, C, D, Other, Unknown<br>If other, please specify.
If antenatally diagnosed, what was the lung-to-head ratio (LHR)?	Enter zero if not undertaken/ not known.
Was foetal tracheal occlusion (FETO) undertaken?<br>If yes, at what gestational age was it inserted?<br>If yes, at was gestational age was it removed?	Yes, No<br>__________, unknown.<br>__________, at birth, unknown.
Liver position?	Chest, Abdomen, Unknown
Did the patient have pulmonary hypertension (at any stage)?<br>If yes, treatment given? If other, please specify.	Yes: diagnosed clinically, Yes: diagnosis confirmed on echocardiography, Yes: other method of confirming diagnosis, No, Unknown<br>Persistent pulmonary hypertension of the newborn (PPHN) is defined as the failure of the normal circulatory transition that occurs after birth. It is a syndrome characterised by marked pulmonary hypertension that causes hypoxemia secondary to right-to-left extrapulmonary shunting of deoxygenated blood. It should be suspected whenever the level of hypoxemia is out of proportion to the level of pulmonary disease. Echocardiography plays a major role in screening and assisting in making the diagnosis of PPHN.<br>Nitric oxide, Prostacyclin, Alprostadil, Milrinone, Other, None: not required, None: required but not available.
Did the patient receive extracorporeal membrane oxygenation (ECMO)?<br>If yes, for how long?	Yes, No<br>In days. Include the day the patient went onto ECMO and the day they were taken off in the calculation.
Primary intervention<br>If patch repair, material used?<br>Other procedures undertaken at the same time?<br>Surgical approach:<br>If laparoscopic or thoracoscopic, was the surgery converted to open?<br>Condition specific complication within 30-days of primary surgery?	Primary repair (absorbable sutures), Primary repair (non-absorbable sutures), Patch repair, Palliation, Discharged with planned elective repair, Other<br>Permacol, PTFE, Alloderm, Dacron, Mesh plug, Muscle flap, Surgisis, Other. If other, please specify.<br>Chest drain insertion, Abdominal wall patch, Fundoplication, Correction of malrotation, Appendicectomy, Other (specify), None<br>Select all that apply. If other, please specify.<br>Laparotomy, Laparoscopy, Thoracotomy, Thoracoscopy, Other (please specify)<br>Yes/No.<br>Air leak (not just redundant space in the pleural cavity which is common), Chylothorax, Recurrence, Adhesional obstruction, Other, None. Select all that apply. If other, please specify.

Intestinal Atresia

Question	Answers
Type of intestinal atresia	Duodenal, Jejuno-ileal, Colonic
Classification of duodenal or colonic atresia<br>Classification of jejuno-ileal atresia	1,2,3,4<br>1) intraluminal web with continuity of the muscular layer, 2) atretic segment without a mesenteric defect, 3) atretic segment with mesenteric defect, 4) multiple atresias = string of sausages appearance.<br>1,2,3a,3b,4<br>1) intraluminal web with continuity of the muscular layer, 2) atretic segment without a mesenteric defect, 3a) atretic segment with mesenteric defect, 3b) apple-peel (bowel wrapped around a single artery), 4) multiple atresias = string of sausages appearance.
Primary intervention for duodenal atresia:<br>Surgical approach<br>Conversion to open procedure?<br>Type of anastomosis<br>Primary intervention for jejuno-ileal and colonic atresia:<br>If bowel was excised, what was the total length of bowel excised?<br>Surgical approach:<br>Conversion to open procedure?<br>Was the distal bowel flushed to check for patency?	Duodenoduodenostomy, Duodenojenunostomy, Web excision only, Palliation, Other. If other, please specify.<br>Laparotomy, Laparoscopy, Endoscopy, Other<br>Yes/ No<br>Kimura's diamond shape, Side-to-side, End-to-end<br>Primary anastomosis, Bowel resection, Division of web only, Loop stoma, Divided stoma, Bishop-Koop stoma, Santulli stoma, Palliation, Other. Select all that apply.<br>In centimetres (cm). Enter 0 if unknown<br>Laparotomy, Laparoscopy, Endoscopy, Other<br>Yes, No<br>Yes, No

For peer review only

If the patient underwent surgery, did they have a condition specific complication within 30-days of primary intervention?	Anastomotic leak, Anastomotic stenosis, Short-gut, Missed additional atresia, Adhesive bowel obstruction, Stoma prolapse, Stoma retraction, Parastomal hernia, Parastomal skin breakdown, Other. If other, please specify. Select all that apply. For the purposes of this study short gut is defined as more than 50% of the small intestine excised (when short bowel syndrome can occur).

Gastroschisis

Question	Answers
Type of gastroschisis	Simple, Complex: associated with atresia, Complex: associated with necrosis, Complex: associated with perforation, Complex: associated with closing gastroschisis. Select all that apply.
Primary intervention:<br><br><br><br><br>Method of defect closure:<br><br><br><br><br>On what day following admission was abdominal wall closure achieved?<br><br>Did the neonate have any of these complications within 30-days of primary intervention?<br><br><br><br><br>If the patient has ACS, was the abdomen re-opened?	Primary closure in the operating room (OR), Primary closure at the cotside (Bianchi technique), Staged closure using a preformed silo, Staged closure using an Alexis Wound Retractor and Protector, Staged closure using a surgical silo (including improvised silo), Other method, No intervention undertaken. If other, please specify.<br>Fascia and skin closed with sutures, Just skin closed with sutures: fascia left open, Umbilical cord sutured over the defect: fascia left open, Sutureless closure with skin edges opposed and dressing applied, Dressing applied: defect left open to close by secondary intention, Other, Patient died before the defect was closed. If other, please specify.<br>In days. Please include the first day of admission and the day of closure in the calculation. For example, for a neonate admitted with gastroschisis on 2nd October who had the defect closed on 4th October, please insert 3 days.<br>Ischemic bowel, Abdominal compartment syndrome (ACS), Necrotising enterocolitis, None of these<br>Select all that apply. ACS is defined as respiratory insufficiency secondary to compromised tidal volumes, decreased urine output caused by falling renal perfusion or any other organ dysfunction caused by increased intra-abdominal pressure.<br>Yes/ No

Exomphalos

Question	Answers
Type of Exomphalos?	Major, Minor<br>Major: >50% of the liver in the exomphalos sac and abdominal wall defect >5cm. Minor: Infants with defects less than 5cm.
Hypoglycaemic on arrival?	Yes, No, Blood glucose not measured<br>Hypoglycaemia is defined as a blood glucose level below 4 mmol/L (72mg/dL).
Primary intervention<br>If the patient had a staged closure, what was the time from primary intervention to closure?<br><br>If conservative management, was a topical treatment applied to the exomphalos sac?<br>If conservative management was undertaken, what is the plan for future management?	Primary operative closure, Staged closure, Conservative management<br>In days. Please include the day of the primary intervention and the day of closure in the calculation. Enter 30 if still not closed at 30-days after primary intervention.<br>Yes: silver sulfadiazine, Yes: betadine, Yes: honey, Yes: merbromide tannage, Yes: other, no. If other, please specify.<br>No further surgery planned, Delayed closure at this hospital, Delayed closure at another hospital, Other. If other, please specify.
Did the patient have a ruptured sac?	Yes, No

Anorectal Malformation (ARM)

Question	Answers
Type of anorectal malformation (Krickenbeck classification)	Low ARM: Perineal (cutaneous) fistula, High ARM: Rectourethral fistula (bulbar), High ARM: Rectourethral fistula (prostatic), High ARM: Rectovesical fistula, High ARM: Vestibular fistula, High ARM: Cloaca, High ARM: No fistula, High ARM: Type unknown at present, Rare variant: Pouch colon, Rare variant: Rectal atresia/ stenosis, Rare variant: Rectovaginal fistula, Rare variant: H fistula, Other
Did the neonate have pre-operative bowel perforation?	Yes, No
What was the primary intervention undertaken?<br><br><br><br><br>If primary anorectal reconstruction was undertaken, was a Peña stimulator or equivalent used to identify the position of the muscle complex intra-operatively?<br>Did the patient have any of the following complications within 30-days of surgery?<br>- Electrolyte disturbance<br>- High output stoma (over 20mls/kg/day)	Fistula dilation: no surgery, Loop sigmoid colostomy, Divided sigmoid colostomy, Loop transverse colostomy, Divided transverse colostomy, Other stoma, Anoplasty, Posterior sagittal anorectoplasty (PSARP), Abdominosacroperineal pull-through, Abdominoperineal pull-through, Laparoscopic-assisted pull-through, Palliative care, Other. If other, please specify. Select all that apply.<br>Yes, no: equipment was not available, no: the equipment was available but not used. Peña stimulator: Muscle locating stimulator commonly used to identify the anal sphincter muscles whilst undertaking a PSARP for patients with ARM.<br>For each of the below answer: Yes, No, Not applicable

For peer review only - http://bmjopen.bmj.com/site/about/guidelines.xhtml

- Stoma prolapse/ retraction/ herniation<br>- Peri-stoma skin breakdown (or perianal if primary reconstructive surgery undertaken without a covering stoma)<br>- Anal stenosis in those undergoing primary anorectal reconstruction without covering stoma.<br>What is the plan for future management?	No further operative management, Anoplasty/ pull-through planned at your hospital, Anoplasty/ pull-through planned at another hospital, Stoma closure planned at your hospital, Stoma closure planned at another hospital, Other<br>Please tick all that apply. If other, please specify.

Hirschsprung's Disease

Question	Answers
Time to first passage of meconium after birth	Less than 24 hours, 24-48 hours, Over 48 hours, Unknown
Features at presentation:	Abdominal distension, Bilious vomiting, Non-bilious vomiting, Poor feeding, Suspected enterocolitis, Perforation, Other. Select all that apply.
Source of diagnosis of Hirschsprung's disease<br><br>If on biopsy, what was the method of histology staining.	Genetic, Mucosal biopsy, Full thickness biopsy, Anorectal manometry, Barium enema, Not confirmed: suspected only, Other.<br>Hemotoxilin and Eosin (H&E), Acetylcholinesterase, Calretinin, Other.<br>Select all that apply. If other, please specify.
Length of aganglionosis:	Rectal, Sigmoid, Descending colon, Transverse colon, Ascending colon, Small bowel, Unknown at present
Primary intervention<br><br><br><br><br><br><br>If primary pull-through was undertaken, did the patient have a covering stoma?<br>Was it laparoscopic assisted?<br>Did the patient have any condition specific complications within 30-days of primary intervention?<br><br><br><br><br><br><br><br><br>What is the plan for future management?	Conservative: no treatment, Conservative: digital stimulation and laxatives, Conservative: regular rectal washouts/ enemas, Failed conservative management followed by a stoma during the same hospital admission, Primary stoma (with or without pre-operative washouts or enemas prior to a planned stoma placement), Primary pull-through (Swenson), Primary pull-through (Duhamel), Primary pull-through (Soave), Primary pull-through (Other), Transanal posterior anorectal myectomy, Palliative care, Other.<br>Yes, No<br><br>Yes, No<br>Hirschsprung's associated enterocolitis (HAEC), Electrolyte disturbance, High stoma output (over 20mls/kg/day), Stoma prolapse/ retraction/ herniation, Peri-stoma skin breakdown (or perianal if primary pull-through was undertaken without a covering stoma), Anal stenosis, Post-operative obstruction, Anastomotic leak (if primary pull-through was undertaken without a covering stoma), Other<br>HAEC is defined as inflammation of the small and or large bowel in patient's born with Hirschsprung's disease. If the patient was managed conservatively, please tick if they developed enterocolitis within 30-days of presentation. Select all that apply.<br>No further surgery planned, Anorectal pull-through at your hospital, Anorectal pull-through at a different hospital, Stoma closure, Other, Unknown

For peer review only

For peer review only - http://bmjopen.bmj.com/site/about/guidelines.xhtml

Management and Outcomes of Congenital Anomalies in Low-, Middle- and High-Income Countries: Protocol for a Multi-centre, International, Prospective Cohort Study

SUPPLEMENTARY FILE 2

Collaborator Survey: Resources and Facilities for Neonatal and Paediatric Surgery

Question	Answers
Title	Professor, Dr, Mr, Mrs, Miss, Ms, Other
Surname	
First Name	
Professional Position	Professor, Consultant or attending, Registrar or resident, Intern/ house officer/ senior house officer, Medical Student, Nurse, Other
Are you the study lead at your centre?	Yes, No<br>Answers provided by the study lead will be used as the 'Gold Standard' to which the other survey will be compared (this will remain anonymous).
Speciality	General Surgery (adult and paediatric), Paediatric Surgery, Anaesthetics, Paediatrics, Neonatology, Nursing, Not specialised yet, Other
Full name of institution:	
Address of institution:	
Country	Afghanistan, Albania, Algeria, Andorra, Angola, Antigua and Barbuda, Argentina, Armenia, Aruba, Australia, Austria, Azerbaijan, Bahamas, Bahrain, Bangladesh, Barbados, Belarus, Belgium, Belize, Benin, Bhutan, Bolivia, Bosnia and Herzegovina, Botswana, Brazil, Brunei, Bulgaria, Burkina Faso, Burma, Burundi, Cambodia, Cameroon, Canada, Cabo Verde, Central African Republic, Chad, Chile, China, Colombia, Comoros, Republic of the Congo, Costa Rica, Cote d'Ivoire, Croatia, Cuba, Curacao, Cyprus, Czechia, Denmark, Djibouti, Dominica, Dominican Republic, Ecuador, Egypt, El Salvador, Equatorial Guinea, Eritrea, Estonia, Eswatini, Ethiopia, Fiji, Finland, France, Gabon, The Gambia, Georgia, Germany, Ghana, Greece, Grenada, Guatemala, Guinea, Guinea-Bissau, Guyana, Haiti, Holy See, Honduras, Hong Kong, Hungary, Iceland, India, Indonesia, Iran, Iraq, Ireland, Israel, Italy, Jamaica, Japan, Jordan, Kazakhstan, Kenya, Kiribati, South Korea, Kosovo, Kuwait, Kyrgyzstan, Laos, Latvia, Lebanon, Lesotho, Liberia, Libya, Liechtenstein, Lithuania, Luxembourg, Macau, Macedonia, Madagascar, Malawi, Malaysia, Maldives, Mali, Malta, Marshall Islands, Mauritania, Mauritius, Mexico, Micronesia, Moldova, Monaco, Mongolia, Montenegro, Morocco, Mozambique, Namibia, Nauru, Nepal, Netherlands, New Zealand, Nicaragua, Niger, Nigeria, North Korea, Norway, Oman, Pakistan, Palau, Palestinian Territories, Panama, Papua New Guinea, Paraguay, Peru, Philippines, Poland, Portugal, Qatar, Reunion Island, Romania, Russia, Rwanda, Saint Kitts and Nevis, Saint Lucia, Saint Vincent and the Grenadines, Samoa, San Marino, Sao Tome and Principe, Saudi Arabia, Senegal, Serbia, Seychelles, Sierra Leone, Singapore, Sint Maarten, Slovakia, Slovenia, Solomon Islands, Somalia, Somaliland, South Africa, South Korea, South Sudan, Spain, Sri Lanka, Sudan, Suriname, Swaziland (See Eswatini), Sweden, Switzerland, Syria, Taiwan, Tajikistan, Tanzania, Thailand, Timor-Leste, Togo, Tonga, Trinidad and Tobago, Tunisia, Turkey, Turkmenistan, Tuvalu, Uganda, Ukraine, United Arab Emirates, United Kingdom, Uruguay, USA, Uzbekistan, Vanuatu, Venezuela, Vietnam, Yemen, Zambia, Zimbabwe
Type of institution (WHO classification)	- **Specialised children's hospital** (Provides highly specialised care dedicated to children)<br>- **Referral hospital** (WHO defined tertiary healthcare. Includes academic, university, teaching, national, central and specialised mission hospitals. Can provide specialised surgical services)<br>- **District hospital** (WHO defined secondary healthcare. Includes provincial, general, general mission or regional hospitals. Has general anaesthesia and can provide general surgical care)<br>- **Health centre** (WHO defined primary healthcare. No general anaesthesia, can do minor local procedures, wound management, triage and referral).
Institutional classification	Government, Non-government
Institutional financial classification	Not for profit, For profit
Population served by your institution (in millions, including children and adults)	
**Personnel**	
Number of Consultant Paediatric Surgeons undertaking	____________________ (Excluding trainees)

For peer review only - http://bmjopen.bmj.com/site/about/guidelines.xhtml

neonatal surgery at your institution:	
Number of Consultant General Surgeons (covering adults and children) undertaking neonatal surgery independently at your institution:	____________________ (Excluding trainees)
Number of medical officers or other non-surgeon healthcare professionals undertaking neonatal surgery independently at your institution	____________________ (Without a consultant surgeon present at the time of surgery)
**Infrastructure** Please state whether the following facilities are available at your institution when required:	
Running Water	Always, Sometimes, Never
Electricity	Always, Sometimes, Never
Electricity generator back-up	Always, Sometimes, Never
Laboratory for biochemistry	Always, Sometimes, Never
Laboratory for haematology	Always, Sometimes, Never
Blood bank	Always, Sometimes, Never
Sterile gloves and gown	Always, Sometimes, Never
Autoclave for sterilising surgical equipment	Always, Sometimes, Never
Neonatal ventilation outside the operating room	Always, Sometimes, Never
Paediatric ventilation outside the operating room	Always, Sometimes, Never
Neonatal intensive care unit for surgical neonates pre and post operatively (including if a stoma is present)	Always, Sometimes, Never
Paediatric intensive care unit for surgical paediatric patients pre and post operatively if required	Always, Sometimes, Never
Parenteral nutrition for neonates	Always, Sometimes, Never
Parenteral nutrition for adults and older children	Always, Sometimes, Never
Extracorporeal membrane oxygenation (ECMO)	Always, Sometimes, Never
Peña stimulator or equivalent device to identify the muscle complex during anorectal reconstruction	Always, Sometimes, Never
Suction rectal biopsy gun to investigate for Hirschsprung's disease	Always, Sometimes, Never
**Procedures** Please state whether the following procedures are available at your institution when clinically appropriate/ required:	
Neonatal laparotomy	Always, Sometimes, Never
Neonatal laparoscopy	Always, Sometimes, Never
Neonatal thoracotomy	Always, Sometimes, Never
Neonatal thoracoscopy	Always, Sometimes, Never
Neonatal central line insertion	Always, Sometimes, Never
Paediatric central line insertion	Always, Sometimes, Never
Umbilical vein catheterisation	Always, Sometimes, Never
Bedside primary reduction and closure of gastroschisis (Bianchi technique)	Always, Sometimes, Never
Preformed silo application, reduction and closure of gastroschisis	Always, Sometimes, Never
Surgical silo application, reduction and closure of gastroschisis	Always, Sometimes, Never
Primary closure of gastroschisis in the operating room	Always, Sometimes, Never
Sigmoid colostomy	Always, Sometimes, Never
Posterior Sagittal Anorectoplasty (PSARP) for anorectal malformation	Always, Sometimes, Never
Foetal tracheal occlusion (FETO) for CDH	Always, Sometimes, Never
Pull-through for Hirschsprung's disease	Always, Sometimes, Never
**Anaesthesia and resuscitation** Please state whether the following facilities are available at your institution when required:	
Neonatal bag, valve and mask	Always, Sometimes, Never
Paediatric bag, valve and mask	Always, Sometimes, Never
Bottled oxygen	Always, Sometimes, Never
Piped oxygen	Always, Sometimes, Never
Oxygen saturation monitor	Always, Sometimes, Never
Apnoea monitor	Always, Sometimes, Never
Multi-parameter intra-operative monitoring	Always, Sometimes, Never
Anaesthetic machine for neonates	Always, Sometimes, Never
Anaesthetic machine for children	Always, Sometimes, Never
Ketamine anaesthesia for neonates	Always, Sometimes, Never
Ketamine anaesthesia for children	Always, Sometimes, Never
Spinal/ caudal anaesthesia for neonates	Always, Sometimes, Never
Spinal/ caudal anaesthesia for children	Always, Sometimes, Never
Anaesthetic doctor competent to perform neonatal anaesthesia	Always, Sometimes, Never
Anaesthetic doctor competent to perform paediatric anaesthesia	Always, Sometimes, Never
Anaesthetic nurse competent to perform neonatal anaesthesia	Always, Sometimes, Never
Anaesthetic nurse competent to perform paediatric anaesthesia	Always, Sometimes, Never

For peer review only

Does your country have at least one specialised children's hospital that can provide neonatal and paediatric surgery?	Yes, No
Any other comments?	

For peer review only

For peer review only - http://bmjopen.bmj.com/site/about/guidelines.xhtml

BMJ Open

Management and Outcomes of Gastrointestinal Congenital Anomalies in Low-, Middle- and High-Income Countries: Protocol for a Multi-Centre, International, Prospective Cohort Study

Journal:	*BMJ Open*
Manuscript ID	bmjopen-2019-030452.R1
Article Type:	Protocol
Date Submitted by the Author:	17-Jun-2019
Complete List of Authors:	Wright, Naomi; King's College London, King's Centre for Global Health and Health Partnerships
**Primary Subject Heading**:	Global health
Secondary Subject Heading:	Paediatrics, Surgery
Keywords:	PAEDIATRIC SURGERY, Paediatric anaesthesia < ANAESTHETICS, Paediatric intensive & critical care < INTENSIVE & CRITICAL CARE, NEONATOLOGY, PAEDIATRICS, Neonatal intensive & critical care < INTENSIVE & CRITICAL CARE

SCHOLARONE™
Manuscripts

Management and Outcomes of Gastrointestinal Congenital Anomalies in Low-, Middle- and High-Income Countries: Protocol for a Multi-Centre, International, Prospective Cohort Study

Global PaedSurg Research Collaboration

Correspondence to: Naomi J. Wright, King's Centre for Global Health and Health Partnerships, School of Population Health and Environmental Sciences, King's College London, Denmark Hill, SE5 9RJ, UK. Email: naomiwright@doctors.org.uk

For peer review only

ABSTRACT

Introduction

Congenital anomalies are the 5th leading cause of death in children under 5-years of age globally, contributing an estimated half a million deaths per year. Very limited literature exists from low- and middle-income countries (LMICs) where most of these deaths occur. The Global PaedSurg Research Collaboration aims to undertake the first multi-centre, international, prospective cohort study of a selection of common congenital anomalies comparing management and outcomes between low-, middle- and high-income countries (HICs) globally.

Methods and Analysis

The Global PaedSurg Research Collaboration consists of surgeons, paediatricians, anaesthetists and allied healthcare professionals involved in the surgical care of children globally. Collaborators will prospectively collect observational data on consecutive patients presenting for the first time, with one of seven common congenital anomalies (oesophageal atresia, congenital diaphragmatic hernia, intestinal atresia, gastroschisis, exomphalos, anorectal malformation and Hirschsprung's disease).

Patient recruitment will be for a minimum of one month from October 2018 to April 2019 with a 30-day post-primary intervention follow-up period. Anonymous data will be collected on patient demographics, clinical status, interventions and outcomes using REDCap. Collaborators will complete a survey regarding the resources and facilities for neonatal and paediatric surgery at their centre.

The primary outcome is all-cause in-hospital mortality. Secondary outcomes include the occurrence of post-operative complications. Chi-squared analysis will be used to compare mortality between LMICs and HICs. Multilevel, multivariate logistic regression analysis will be undertaken to identify patient level and hospital level factors affecting outcomes with adjustment for confounding factors.

Ethics and Dissemination

At the host centre this study is classified as an audit not requiring ethical approval. All participating collaborators have gained local approval in accordance with their institutional ethical regulations. Collaborators will be encouraged to present the results locally, nationally and internationally. The results will be submitted for open access publication in a peer reviewed journal.

Registration Details

This study has been registered with ClinicalTrials.Gov, identifier: NCT03666767. The registration is available to view via: https://goo.gl/ffXNMH

For peer review only - http://bmjopen.bmj.com/site/about/guidelines.xhtml

Strengths and Limitations of this Study

- This will be the first large-series, geographically comprehensive, multi-centre, international, prospective cohort study to define the management and outcomes of a selection of common congenital anomalies in low-, middle- and high-income countries across the globe.
- The collaborative approach for this study allows a large series of high-quality data to be collected in a timely manner without overburdening high-volume, low-resource centres.
- The seven study conditions constitute a selection of the commonest life-threatening congenital anomalies requiring emergency surgical care in the neonatal period **(Box 1)**.
- We recognise that some children may not reach a facility capable of providing acute paediatric surgical care and hence the results obtained may be an underestimation of true morbidity and mortality, especially in LMICs.
- The number of variables being collected per patient has been limited to those known to have the greatest impact on outcomes to optimise the feasibility of the study; follow-up is limited to 30-days post-primary intervention.

For peer review only

INTRODUCTION

In 2015, the Global Burden of Disease study concluded congenital anomalies (also known as congenital malformations, congenital abnormalities or birth defects) to be the 5th leading cause of death in children under 5-years of age globally.[1] This equates to approximately half a million deaths from congenital anomalies each year, 97% of which occur in low- and middle-income countries (LMICs). Indeed, this is likely to be an underestimation of the actual number of deaths due to under-diagnosis of neonates with congenital anomalies who die in the community and a lack of death certification in many LMICs.[2] Not only is the mortality rate higher in LMICs, the incidence and prevalence are also higher due to micronutrient deficiencies, infections and teratogens during pregnancy resulting in more cases and a lack of antenatal diagnosis prohibiting terminations.[3,4] There is limited research and a lack of congenital anomaly registries in LMICs and hence they have received very little global attention.[5]

The conditions forming the focus of this study **(Box 1)** constitute a selection of the most common life-threatening congenital anomalies during the neonatal period, which involve the gastrointestinal tract. They each have an incidence of 1/2000 – 1/5000, they collectively form up to 40% of emergency neonatal surgery and associated mortality can be in excess of 50% in many LMICs.[6-9] Disparities in outcomes globally can be stark; for example the mortality from gastroschisis is 75-100% in many LMICs compared to 4% or less in HICs.[10-12] Reasons for poor outcomes include a lack of antenatal diagnosis, delayed presentation, limited neonatal transport and in-hospital resources, a dearth of trained support personnel and a lack of intensive care and parenteral nutrition for neonates.[9,13,14] In Uganda, it was calculated that only 3.5% of the need for neonatal surgery was met by the healthcare system.[8]

Box 1. Congenital Anomalies in the Global PaedSurg Study

- Oesophageal atresia (OA) +/- tracheo-oesophageal fistula (TOF)
- Congenital diaphragmatic hernia (CDH)
- Intestinal atresia (IA)
- Gastroschisis
- Exomphalos
- Anorectal malformation (ARM)
- Hirschsprung's disease

In 2010, the World Health Assembly passed a resolution recommending 'prevention whenever possible, to implement screening programmes and to provide care and ongoing support to children with birth defects and their families'.[2] Prevention is paramount, however this is not yet possible for many congenital anomalies and hence a focus on improving postnatal care and outcomes is vital. The Sustainable Development Goal 3.2 aims to end preventable deaths of newborns and children under the age of 5-years by 2030.[6, 15,16] With a third of infant deaths being attributed to congenital anomalies, clearly this will not be achievable without an accelerated effort towards the provision of surgical care for children. It is estimated that two-thirds of deaths and disability from congenital anomalies can be avoided with the provision of neonatal and paediatric surgical care.[6] Indeed, studies have demonstrated such provision can be highly cost-effective in terms of disability adjusted life years saved.[5] Yet neonatal and paediatric surgical care remain a low priority on the global health agenda.[5]

A shift is needed to focus on the provision of surgical care for children within National Health Plans and International Organisations and to elevate congenital anomalies on the global health agenda. This large-scale, geographically comprehensive, multi-centre prospective cohort study aims to define the current management and outcomes of a selection of common congenital anomalies globally and identify factors affecting outcomes that can be modified to improve care. This is vital to aid advocacy and global health prioritisation and inform future interventional studies aimed at improving outcomes.

AIM

For peer review only - http://bmjopen.bmj.com/site/about/guidelines.xhtml

To undertake the first large-scale, geographically comprehensive multi-centre, prospective cohort study comparing the management and outcomes of a selection of common congenital anomalies in low-, middle-, and high-income countries across the globe.

OBJECTIVES

1. To compare the mortality and post-intervention complications of a selection of common congenital anomalies involving the gastrointestinal tract in LMICs and HICs globally.

2. To identify patient level and hospital level factors affecting outcomes that be modified to improve care.

3. To establish a research collaboration consisting of children's surgical care providers across the world to help enhance research capacity and to create a platform for ongoing collaborative research and intervention studies aimed at improving outcomes.

4. To raise awareness and provide advocacy for neonatal and paediatric surgical care within global health prioritisation, planning, policy and funding.

METHODS AND ANALYSIS

Study Design

This is an international, multi-centre, prospective observational cohort study. The Global PaedSurg Research Collaboration consisting of children's surgical care providers (collaborators) across the world was established from November 2017 to co-ordinate the study at an institutional level and facilitate data collection. Collaborators are free to choose one or more months between 1st October 2018 to the 30th April 2019 (inclusive) to recruit consecutive patients to the study, with a 30-day post-primary intervention follow up period. The primary intervention must occur within 30-days of presentation to be included in the study. Hence, the last date for primary data collection is 29th June 2019. Following this there will be a period of data collection for the data validation process continuing until the end of July 2019.

Collaborators

International collaborators will have a variety of roles and responsibilities within the study. Local collaborators will establish mini-teams locally, gain study approval, utilise the protocol criteria to appropriately identify patients for study inclusion, collect prospective data and upload it to REDCap. Each hospital will have a local study lead who will hold overall responsibility for ensuring the data is accurate, complete and without duplications. Country-lead collaborators will help to recruit other collaborators from within their country and provide advice and support regarding gaining local study approval and data collection. They may also help with translation of the study literature to the local language if required. Continent and regional leads will help to recruit country leads, provide them with advice regarding the study and also encourage and co-ordinate presentations of the protocol at national and international meetings. Lead investigators will contribute to the study design through the provision of feedback from the pilot studies undertaken in multiple languages. An organising committee will help to co-ordinate all study activities and a steering committee will provide guidance throughout.

There are a number of benefits for collaborators participating the study. Publishing journal(s) will be asked to make all collaborators PubMed citable co-authors. This is based on an equal partnership model described by the Lancet and is used by a number of national and international collaboratives.[17-21] All collaborators will be listed as an author on resulting presentations. Collaborators will have the opportunity to present the study locally, nationally and internationally, initially the study protocol and later the results. This often provides collaborators, especially those who are junior or from LMICs, the opportunity to apply for funding to attend, present and network at such meetings. Participation in the study provides an easy route and insight into clinical research, which can be further established

For peer review only - http://bmjopen.bmj.com/site/about/guidelines.xhtml
For peer review only

through participation in the 2-year Research Training Fellowship which is running alongside the main study free of charge for all interested collaborators.

Sample Selection

Collaborator and Hospital Inclusion Criteria:

All hospitals and healthcare professionals providing surgical care for neonates and children, presenting for the first time, with one or more of the study conditions can be included in the study. Collaborators should gain permission from the senior surgeon or physician who oversees the care of the children to be included in the study in order to participate. There can be up to three collaborators in a mini-team per month of data collection. One mini-team can collect data over one or more months or several mini-teams can collect data over a different month each. Each mini-team must contain at least one senior surgeon or physician to oversee the data collection process.

Patient Inclusion and Exclusion Criteria:

Any neonate, infant or child under the age of 16-years, presenting acutely for the first time, with one or more of the study conditions can be included in the study. Patients who have previously received surgery for their presenting condition or those representing with a complication of surgery are excluded. Patients presenting electively for surgery are excluded. Children who have received basic resuscitative care for their condition at a different healthcare facility and are then transferred to the study centre for their primary surgical intervention can be included. Children who only receive resuscitative treatment at the study centre and are then referred elsewhere for their primary surgical intervention cannot be included since the outcome of the surgical care will not be known and also to avoid the risk of duplicate patients in the study. Patients who receive conservative treatment as their primary intervention, palliative care, or no care must be included within the study to accurately reflect the management and outcomes of all presenting cases.

If a patient presents with more than one of the study conditions, the details of each condition that they present acutely with can be included, but not a previously managed condition. For example, a newborn presenting with oesophageal atresia and anorectal malformation would have both conditions included. A patient presenting for the first time with Hirschsprung's disease at several months of age who had a duodenal atresia repaired at birth would have the full details of the Hirschsprungs disease included, but the duodenal atresia would simply be noted as an associated anomaly.

Outcome Measures

The primary outcome is all-cause, in-hospital mortality.

For patient's hospitalised for over 30-days following primary intervention, a 30-day post-primary intervention mortality rate will be utilised. Those who do not receive a primary intervention, but remain alive and hospitalised at 30-days following primary admission, will have this time point used for recording their mortality status for the primary outcome. Primary outcome is defined in Table 1.

The secondary outcomes include complications occurring within 30-days of primary intervention:

- Surgical site-infection
- Wound dehiscence
- Need for re-intervention
- Condition specific complications
- Condition specific outcome variables
- Length of hospital stay or time from admission to death in patients who do not survive
- 30-day post primary intervention mortality.

Secondary outcomes will not be collected on patients who do not receive a primary intervention within 30-days of hospital admission, with the exception of length of hospital stay or time from admission to

For peer review only - http://bmjopen.bmj.com/site/about/guidelines.xhtml

death. 30-day follow-up will be undertaken within the capacity of the collaborating team; no additional funding will be provided.

Data Collection

Generic variables relating to the patient demographics, antenatal care, pre-hospital care, clinical condition, surgical intervention and outcomes will be collected for all patients in the study **(Table 1)**. Specific variables will be collected for each individual condition (**Supplementary File 1).**

Outcomes and variables have been chosen using published core outcome sets and commonly collected outcomes in systematic reviews and meta-analyses.[22-37] Collaborators will enter anonymous, de-identified data via the secure internet-based Research Electronic Data Capture (REDCap) system. This will be stored on King's College London REDCap server.

A short survey will be completed by the local study lead and one other collaborating consultant or registrar on the resources and facilities available for neonatal and paediatric surgical care at their centre **(Supplementary File 2)**.

Table 1. Generic Data Points

Generic questions	Answers
During which month did the patient present to your hospital?	Please select the month that the patient presented to your hospital for the first time with this congenital anomaly. For example, if a baby was born with gastroschisis on the 29th September and presented to your hospital on the 1st October you should select October.
Has consent been provided to include this patient in the study?<br>If no, which condition did the patient present with?	Yes / No / Patient consent is not required for this study at my institution<br><br>Oesophageal atresia / Congenital diaphragmatic hernia / Intestinal atresia / Gastroschisis / Exomphalos / Omphalocele / Anorectal malformation / Hirschsprung's Disease.<br>Please select all the conditions that the patient presented with. Do not select a condition which the patient has already received surgical treatment for previously.
**Demographics**	
Gestational age at birth	Number of weeks from the first day of the women's last menstrual cycle until birth. Round up or down to the nearest week.
Age at presentation (in hours)	We understand this information may be difficult to obtain - please be as accurate as you can. Please round to the nearest hour. This number may be very large for patients who have a delayed presentation - please still enter it. For neonates born within your centre please enter 0. Enter unknown if unknown.
Gender	Male / Female/ Ambiguous/ Unknown
Weight at presentation	In kilograms (kg) on the day of presentation. Please provide a value to 1 decimal place.
Does the patient have another anomaly in addition to the study condition?	Yes, Cardiovascular, Yes, Respiratory, Yes: Gastrointestinal, Yes: Neurological, Yes: Genito-urinary, Yes: Musculoskeletal, Yes: Down syndrome, Yes: Beckwith-Wiedemann syndrome, Yes: Cystic fibrosis, Yes: Chromosomal, Yes: Other, No<br>Select all that apply. Include all anomalies diagnosed at any stage up until 30-days post primary intervention or 30-days following presentation for those who didn't receive an intervention. If you suspect an associated anomaly, but it has yet to be diagnosed, select 'other'.
Distance from the patient's home to your hospital	In kilometres (km). Please round to the nearest kilometre. Please enter 0 if born in your hospital.
**Antenatal Care and Delivery**	
Antenatal ultrasound undertaken?<br><br>If the condition was diagnosed antenatally, at what gestational age?	Yes: study condition diagnosed, Yes: problem identified but study condition not diagnosed, Yes: no problem identified, No<br>Please round up to the nearest week. If the patient has more than one study condition, please note the gestational age at which one or more of the conditions was first diagnosed.
Mode of transport to hospital?<br><br>Where did the patient present from? If other, please specify.	Ambulance, Other transport provided by the health service, Patient's own transport, Born within the hospital<br>Home / Community Clinic / General Practice / District Hospital / Other / Unknown<br>District hospital includes: secondary level healthcare, provincial hospital, general hospital, general mission hospital or regional hospital. It has general anaesthesia and can provide general surgical care.
Type of delivery:	Vaginal (spontaneous), Vaginal (induced), Caesarean section (elective), Caesarean section (urgent/non-elective), Unknown. Vaginal delivery includes those requiring forceps and ventouse.
**Clinical condition and patient care**	
Was the patient septic on arrival?	Yes, no<br>Sepsis is SIRS (Systemic Inflammatory Response Syndrome) with a suspected or confirmed bacterial, viral, or fungal cause. SIRS is a response to a stimulus, which results in two or more of the following: temperature > 38.5°C or < 36°C, tachycardia*, bradycardia* in children < 1 year old, tachypnoea*, leukopenia or leucocytosis*, hyperglycaemia*, altered mental status, hyperlactaemia*, increased central capillary refill time >2 seconds. *Variables are defined as

For peer review only - http://bmjopen.bmj.com/site/about/guidelines.xhtml

If yes, were appropriate antibiotics administered?	values outside the normal range for age. Arrival is the time of birth for neonates born at your hospital.<br>Yes: within 1 hour of arrival, Yes: within the first day of arrival, No<br>Appropriate antibiotics are defined as either broad spectrum covering gram negative, gram positive and anaerobic bacteria OR antibiotics that are the standard empirical treatment for that condition according to local guidelines OR are based on sensitivities provided by a microbiology sample.
Was the patient hypovolaemic on arrival?<br><br>If yes, was an intravenous fluid bolus given?<br>If yes, how much intravenous fluid was given?	Yes/ No. Criteria for diagnosis include at least one of the following: prolonged central capillary refill time > 2 seconds, *tachycardia, mottled skin, *reduced urine output, cyanosis, impaired consciousness, *hypotension. *Variables are defined as values outside the normal range for age.<br>Yes: within 1 hour of arrival, Yes: on the first day of arrival, No<br>10 - 20mls/ kg, above 20mls/ kg<br>If less than 10mls/ kg was given please select 'no' for the question asking if intravenous fluid was given.
Was the patient hypothermic on arrival?<br><br>If yes, was the patient warmed on arrival to within a normal temperature range?	Yes/ No. Defined as < 36.5 degrees Celsius core temperature. Arrival is the time of birth for neonates born at your hospital.<br>Yes/ No. Only select yes if warming was commenced within 1 hour of arrival. Arrival is the time of birth for neonates born at your hospital.
Did the patient receive central venous access?<br><br>If yes, did the patient acquire central line sepsis?	Yes: umbilical catheter, Yes: peripherally inserted central catheter (PICC), Yes: percutaneously inserted central line with ultrasound guidance, Yes: surgically placed central line (open insertion), No.<br>Please select all that the patient received within 30-days of primary intervention or 30-days of presentation if no intervention was undertaken.<br>Yes: diagnosed clinically, Yes: confirmed on microbiology, No<br>Within 30-days of primary intervention or 30-days of presentation if no intervention was undertaken.
Time from arrival at your hospital to primary intervention in hours	(enter 0 if no intervention was undertaken)<br>**Primary intervention for each condition is defined as: Oesophageal atresia**; surgery, either temporising or definitive, to manage the oesophageal atresia and/ or tracheo-oesophageal fistula. **Congenital diaphragmatic hernia**; surgery to reduce the hernia and close the defect. **Intestinal atresia**; surgery, either temporising or definitive, to manage the obstruction including stoma formation and primary anastomosis. **Gastroschisis**; any procedure to either cover or reduce the bowel and/ or close the defect. This includes application of a silo (regardless of whether or not they go on to require surgery). It excludes initial covering of the bowel in a plastic covering (bag or cling film) prior to intervention. **Exomphalos**; surgery or application of topical treatment to the sac in patients managed conservatively (regardless of whether or not they go on to require surgery). **Hirschsprung's disease**; surgery, either temporising or definitive, or rectal/ distal bowel irrigation, laxatives or digital stimulation in patients managed conservatively. This does not include pre-operative washouts in patients planned to have surgery. **Anorectal malformation**; surgery, either temporising or definitive, or anal/ fistula dilatation in patients with a low anorectal malformation managed conservatively.
American Society of Anesthesiologists (ASA) Score at the time of primary intervention	1. Healthy person, 2. Mild systemic disease, 3. Severe systemic disease, 4. Severe systemic disease that is a constant threat to life, 5. A moribund patient who is not expected to survive without the operation, Not applicable - no intervention
What type of anaesthesia was used for the primary intervention?	General anaesthesia with endotracheal tube, General anaesthesia with laryngeal airway, Ketamine anaesthesia, Spinal/ caudal anaesthesia, Local anaesthesia only, No anaesthesia/ just analgesia, No anaesthesia/ no analgesia, Not applicable: no surgery or intervention undertaken.
Who undertook the anaesthetic for the primary intervention?	Anaesthetic doctor, Anaesthetic nurse, Medical officer, Surgeon, Other healthcare professional, No anaesthetic undertaken<br>If more than one of these personnel were present please select the most senior.
Who undertook the primary intervention?	Paediatric surgeon (or junior with paediatric surgeon assisting/ in the room), General surgeon (or junior with paediatric surgeon assisting/ in the room), Junior doctor, medical officer or other (without a paediatric or general surgeon assisting/ in the room), Trainee surgeon (without a paediatric or general surgeon assisting or in the room), Not applicable - no surgery or primary intervention undertaken.
Was a Surgical Safety Checklist used at the time of primary intervention?	Yes, No: but it was available, No: it was not available, Not applicable: a conservative primary intervention was undertaken, Not applicable: no surgery or primary intervention undertaken
Total duration of antibiotics following primary intervention	In days (including the day of surgery and the day antibiotics were stopped. Include intravenous and oral antibiotics).
Did the patient receive a blood transfusion?	Yes: not cross-matched, Yes: cross-matched, No: not required, No: it was required but not available. Within 30-days of primary intervention or 30-days of presentation if no intervention was undertaken.
Did the patient require ventilation?<br><br>If yes, for how long did the patient remain on ventilation?	Yes: and it was given, Yes: but it was not available, No<br>Within 30-days of primary intervention or 30-days of presentation if no intervention was undertaken. Please include all types of ventilation.<br>In days (include all days on ventilation within 30-days of primary intervention or 30-days of presentation if no intervention was undertaken).
Time to first enteral feed (post-primary intervention)	In days (include the day of primary intervention and the day of first enteral feed in the calculation). Enter 0 if enteral feeds were not commenced. Enter 999 if feeds were not stopped, for example in patients with Hirschsprung's Disease managed conservatively. Include all types of enteral feeding - oral, nasogastric, gastrostomy and other.
Time to full enteral feeds (post-primary intervention)	In days (enter 0 if the patient died before reaching full enteral feeds or 30 if the patient had not reached full enteral feeds at 30-days post primary intervention or 30-days following admission in

For peer review only

	patients who did not receive a primary intervention). Include all types of enteral feeding - oral, nasogastric, gastrostomy and other.
Did the patient require parenteral nutrition?<br><br>If yes, for how long did the patient receive parenteral nutrition?	Yes and it was given, Yes and it was sometimes available but less than required, Yes but it was not available, No<br>In days. Include all days that the patient received parenteral nutrition (any volume) up until 30-days post primary intervention or 30-days following presentation in patients who do not receive an intervention.
**Outcomes**	
Did the patient survive to discharge?<br><br><br>If the patient was discharged prior, were they still alive at 30-days following primary intervention?<br><br>If no, cause of death?	Yes/ No<br>Select yes if the patient was still alive in your hospital 30-days after primary intervention or 30-days after presentation in patients who do not receive a primary intervention.<br>Yes, No: not followed-up after discharge, Followed-up but not until 30-days post primary intervention<br>This can include all reliable communication with the patient/ patient's family including in person, via telephone and other.<br>Sepsis, Aspiration pneumonia, Respiratory failure, Cardiac failure, Malnutrition, Electrolyte disturbance, Haemorrhage, Lack of intravenous access, Hypoglycaemia, Recurrent tracheo-oesophageal fistula, Recurrent diaphragmatic hernia, Anastomotic leak, Ischaemic bowel, Ruptured exomphalos sac, Enterocolitis, Other. If other, please specify
Duration of hospital stay (days)	Please include the day of admission and the day of discharge in your calculation. For example, if a patient presented on 1st October and was discharged on the 5th October, their duration of hospital stay would be 5 days. If the patient died, please record the number of days from admission to death. Only include the duration of the primary admission, not subsequent admissions if the patient re-presented.
Did the patient have a surgical site infection?	Yes, No, Not applicable: no surgical wound<br>This is defined as one or more of the following within 30-days of surgery: 1) purulent drainage from the superficial or deep (fascia or muscle) incision, but not within the organ/ space component of the surgical site OR 2) at least two of: pain or tenderness; localised swelling; redness; heat; fever; AND the incision is opened deliberately to manage infection, spontaneously dehisces or the clinician diagnoses a SSI (negative culture swab excludes this criterion) OR 3) there is an abscess within the wound (clinically or radiologically detected).
Did the patient have a full thickness wound dehiscence?	Yes, No, Not applicable - no surgical wound.<br>This is defined as all layers of the wound opening within 30-days of surgery
Did the patient require a further unplanned intervention?	Yes - percutaneous intervention, Yes - surgical intervention, No, Not applicable - no primary intervention undertaken. Within 30-days of primary intervention. This does not include routine reduction and closure of the defect in neonates with gastroschisis receiving a preformed silo.
Was the patient followed up at 30-days post primary surgery or intervention to assess for complications?	Yes: reviewed in person, Yes: via telephone consultation, Yes: via other means, Yes: still an in-patient at 30-days, No: data is based on in-patient observations only, No: follow-up was done but prior to 30-days
If the patient had a complication, when was it diagnosed?	During the primary admission, As an emergency re-attender, At routine follow-up as an out-patient, Not applicable: no complications
What study condition does this patient have?	Oesophageal atresia, Congenital diaphragmatic hernia, Intestinal atresia, Gastroschisis, Exomphalos/ Omphalocele, Anorectal malformation, Hirschsprung's Disease<br>If the patient has presented for the first time with more than one of these conditions please select all that apply. If the patient presented on this occasion with one of these conditions, but previously had another condition managed then only select the condition they are presenting with on this occasion and enter that they have another anomaly in the demographics section above. For example, if the patient presents at 2-months with Hirschsprung's disease, but previously had a duodenal atresia repair please select Hirschsprung's disease here (not intestinal atresia) and tick in the section above that they have another gastrointestinal anomaly.

For peer review only

Data Quality

To ensure high quality of data, a detailed protocol for collaborators has been produced and published on the study website (www.globalpaedsurg.com) in 12 languages: English, French, Spanish, Portuguese, German, Italian, Chinese, Arabic, Korean, Lithuanian, Turkish and Russian. Clear and concise definitions have been provided for all data points on the protocol, on the data collection forms and within REDCap when entering the data. A study launch meeting was undertaken where the principal investigator presented the data collection process in detail, demonstrated use of REDCap and answered questions. This was recorded, circulated to all collaborators via email and placed on the website. A frequently asked questions document has been circulated via email and placed on the website. Two meetings were held by the principal investigator to detail the study, data collection process and answer questions amongst the country leads so they in turn can provide advice and support to local collaborators within their country. Again this was recorded, circulated and placed on the website.

A pilot study of the patient data collection form and institutional survey was undertaken by lead investigators to optimise the study design and to address any feasibility or other barriers to effective data collection and study completion across participating sites. The pilot study commenced on 1st

For peer review only - http://bmjopen.bmj.com/site/about/guidelines.xhtml

August 2018 for 30 days in English, Spanish and French by 41 collaborator colleagues. The data collection forms were amended following feedback to clarify terminology, add important missing variables or descriptions and correct any translation errors. All translated data collection forms, REDCap and study documentation has been checked and verified by a native speaker for accuracy.

Data Validation

Ten percent of collaborating centres will be selected at random for data validation by an independent research collaborator. The aim will be to determine the numbers of patients eligible during the data collection period to check if any were missed and collect a selection of data again to cross-check for accuracy. Validating questions have been built into the data collection tool. At least 90% of primary and secondary outcomes must be completed for each patient. All collaborators within validating centres will be asked to complete a brief survey regarding their experience with data collection to identify any potential errors and to aid with data interpretation.

Sample Size Calculation

A sample size calculation was undertaken using Stata/IC 15.0 based on Bonferroni correction for multiple testing, assuming 80% power and an overall type 1 error of 5%. The required sample size for each condition has been calculated for the primary outcome of mortality in LMICs compared to HICs and also low, middle and high-income countries separately **(Table 2).** Mortality estimations are based on pooled data from published studies on these conditions in low-, middle- and high-income countries respectively.

Based on the patient numbers included in the previously undertaken PaedSurg Africa study, which utilised a similar study design, the estimated sample sizes to detect a significant difference between LMICs and HICs in this study are achievable.[13]

Table 2. Estimated mortality and sample sizes for low, middle and high-income countries and the mean number of cases per month per institution globally

Condition	Mortality LIC (%, n)	Mortality MIC (%, n)	Mortality LMIC combined (%, n)	Mortality HIC (%, n)	Sample size for LIC	Sample size for MIC	Sample size for HIC	Sample size for LMIC vs HIC (per group)	Mean no. cases/ month/ institution (L,M&HIC combined)
OA +/- TOF	79.5% (62/78)	41.8% (623/1488)	43.7% (685/1566)	2.7% (6/221)	34	34	23	21	1.02
CDH	-	47.4% (130/274)	47.4% (130/274)	20.4% (201/982)	-	-	-	63	0.54
IA	42.9% (42/98)	40.0% (97/241)	41.0% (139/339)	2.9% (12/407)	6014	6014	25	24	0.63
Gastroschisis	83.1% (211/254)	42.6% (205/481)	56.6% (416/735)	3.7% (28/748)	29	29	24	15	0.85
Exomphalos	25.5% (41/161)	31.9% (132/414)	30.1% (173/575)	12.7% (40/316)	1040	1040	196	115	0.63
ARM	26.3% (26/99)	17.5% (243/1391)	18.1% (269/1490)	3% (14/462)	460	460	90	85	1.34
Hirschsprung's Disease	19.1% (33/173)	16.8% (55/328)	17.6% (88/501)	2.3% (43/1897)	5802	5802	85	79	2.21

Estimated Study Population

The mean number of cases presenting to an institution per month for each study condition was estimated from published studies across all income settings **(Table 2)**. On average most institutions caring for patients with these conditions receive 1-2 new cases per month; each participating institution would expect approximately 7-14 new cases in the study per month although this can vary. The aim is to include a minimum of 365 months of data; 183 months from LMICs and 183 months from HICs. This should ensure enough cases of exomphalos to determine a significant difference between LMICs and HICs; fewer months of data are required to determine significant differences between other study conditions. An up-to-date total of patient numbers within the study will be maintained on the study website.

For peer review only - http://bmjopen.bmj.com/site/about/guidelines.xhtml

Data Analysis

Patient and Institutional Data:

Data will be analysed using Stata and SAS 9.4 (Cary, NC; USA). Missing data for the covariates will be analysed to determine whether it is related to the outcome and either complete-case analyses or multiple imputation techniques will be used for analyses accordingly.

Significant differences in mortality between LMICs and HICs will be determined for each of the study conditions using Chi-squared analysis, or Fischer's exact test if either group contains less than 10 patients. World Bank classification of low-, middle- and high-income countries during the fiscal year 2018 will be used.[38]

Univariate logistic regression analyses will be conducted between covariates and the primary outcome of mortality. Based on the results, covariates with a p-value of <0.10 will be included in the multivariate model. The final multi-level multivariate logistic model will be determined using stepwise backward elimination to interventions and peri-operative factors affecting outcomes. Data will be adjusted for confounding factors and effect modifiers. Potential confounders include: gestation age at birth, weight, time from birth to presentation and ASA score at the time of primary intervention. Potential effect modifiers include: administration of peri-operative antibiotics, fluid resuscitation, thermal control and provision of other condition specific neonatal care such as parenteral nutrition in neonates with gastroschisis.

Multi-level multivariate logistic regression analysis will also be undertaken to identify institutional factors affecting mortality with adjustment for confounders. $P < 0.05$ will be deemed significant.

Data Validation:

A weighted kappa statistic will be utilised to determine the level of agreement between the patient data in the main study and the validation data. A weighted kappa statistic will be also utilised to determine the level of agreement between institutional surveys independently completed by the local study lead and one other consultant or registrar at each participating centre. Results will be presented as a proportion of agreement for each variable being validated.

Patient and Public Involvement

CDH UK, a patient and family advisory group and charity, provided input into the design of the study protocol and data collection tool. Their input will be sought on the findings and dissemination of the results.

ETHICS AND DISSEMINATION

Research Ethics Approval

The study has been classified as an audit at the host institution and hence did not require ethical approval. The study fulfils the audit criteria as follows: 1) All data collected measures current practice. The study does not involve any changes to patient management; 2) Current practice and outcomes in low, middle and high-income countries will be compared to published standards in the literature. **Table 2** details the current mortality standards for each of the seven study conditions in high-income countries; 3) All the study data is routinely collected information which should be known to the study team without asking additional questions to the patients/parents; 4) All data to be entered into REDCap is entirely anonymous; 5) No individual patient, collaborator, institution or country will be independently identifiable in the study results; 6) All data will be stored securely and will be governed by King's College London data protection team.

For peer review only - http://bmjopen.bmj.com/site/about/guidelines.xhtml
For peer review only

Research collaborators were required to gain approval to participate in the study at their institution according to their local ethical regulations. Data transfer agreements were legally signed between institutions where required. The participating institutions, type of study approval and study approval reference numbers are detailed in Supplementary File 3. It was not mandated for study approvals to be translated into English. Hence, some reference numbers are in the local scripture of the participating country and have therefore not been incorporated into the table.

Study Dissemination

The study concept and design will be presented at international conferences in order to recruit collaborators. Following completion, the results will be presented at local, national and international conferences globally. Both the promotional presentations of the study protocol and the study results will be presented by study collaborators of all levels of training, disciplines and regions of the world. The results will be submitted for open access publication in a peer reviewed journal. Following publication, the full anonymous, de-identified dataset will be made publicly available via an online repository. Collaborators will have the opportunity to undertake sub-analyses of the data for their country (if all collaborators from that country agree), region or continent.

DISCUSSION

This study aims to define, for the first time, the management and outcomes of a selection of common life-threatening congenital anomalies across the globe. This will help to raise awareness of the unacceptable disparities in outcomes between low-, middle- and high-income countries and the need to focus on improving access to quality surgical care for neonates with congenital anomalies within national health plans and global health prioritisation. It is hoped that factors affecting mortality and morbidity will be identified that can be modified to improve care. Establishment of the Global PaedSurg Research Collaboration developed during this study will create a platform for ongoing collaborative work and interventional studies aimed at improving outcomes in the future.

For peer review only

ADDITIONAL INFORMATION

Twitter: @GlobalPaedSurg

Website: http://globalpaedsurg.com

Author Contributions: The principal investigator conceived the idea for the study, gained study funding, wrote the study protocol, designed the data collection tools, established the study team, co-ordinated the pilot study, revised the study design/ data collection tools following feedback and made critical revisions to the manuscript for publication. The steering committee contributed critical input and revisions to the funding application, study design, protocol and manuscript for publication. The writing committee drafted the protocol manuscript for publication and contributed as organising committee members. The organising committee assisted in the recruitment of and communication with collaborators to participate in the pilot study, helped to co-ordinate the pilot study and summarise the feedback, made critical revisions to the data collection tools in multiple languages and contributed to the study design. The lead investigators contributed to the study design and content of the data collection forms through feedback following participation in the pilot study. All contributed to the content of this manuscript.

Funding Statement: Naomi Wright, Principal Investigator, is funded by the Wellcome Trust through a Clinical PhD in Global Health undertaken at King's College London (Funder Reference: 203905/Z/16/Z). The Wellcome Trust had no input into the study protocol other than to recommend open-access publication in a peer-reviewed journal and to make the anonymised dataset publicly available. Nick Sevdalis' (NS) research is supported by the National Institute for Health Research (NIHR) Collaboration for Leadership in Applied Health Research and Care South London at King's College Hospital NHS Foundation Trust. NS is a member of King's Improvement Science, which is part of the NIHR CLAHRC South London and comprises a specialist team of improvement scientists and senior researchers based at King's College London. Its work is funded by King's Health Partners (Guy's and St Thomas' NHS Foundation Trust, King's College Hospital NHS Foundation Trust, King's College London and South London and Maudsley NHS Foundation Trust), Guy's and St Thomas' Charity, the Maudsley Charity and the Health Foundation. NS and Andrew Leather are also supported by the NIHR Global Health Research Unit on Health System Strengthening in Sub-Saharan Africa, King's College London (GHRU 16/136/54) and by the ASPIRES research programme in LMICs (Antibiotic use across Surgical Pathways - Investigating, Redesigning and Evaluating Systems), funded by the Economic and Social Research Council. The views expressed are those of the authors and not necessarily those of the NHS, the NIHR or the Department of Health and Social Care.

Competing Interest's Statement: Nick Sevdalis is the director of the London Safety and Training Solutions Ltd, which offers training in patient safety, implementation solutions and human factors to healthcare organisations. No other conflicts of interest are declared.

Patient Consent: Collaborators must follow their local ethical guidelines regarding patient consent.

Ethics Approval: This study has been classified as a clinical audit with written confirmation from King's College London Ethics Committee that it does not therefore require ethical approval. All participating centres have gained study approval to participate according to their local institutional ethical regulations (Supplementary File 3).

Provenance and Peer Review: Not commissioned; externally peer reviewed.

Open Access: This is an Open Access article distributed in accordance with the terms of the Creative Commons Attribution (CC BY 4.0) license, which permits others to distribute, remix, adapt and build upon this work, for commercial use, provided the original work is properly cited.

Licence Statement: I, the Submitting Author has the right to grant and does grant on behalf of all authors of the Work (as defined in the below author licence), an exclusive licence and/or a non-exclusive licence for contributions from authors who are: i) UK Crown employees; ii) where BMJ has

For peer review only - http://bmjopen.bmj.com/site/about/guidelines.xhtml

agreed a CC-BY licence shall apply, and/or iii) in accordance with the terms applicable for US Federal Government officers or employees acting as part of their official duties; on a worldwide, perpetual, irrevocable, royalty-free basis to BMJ Publishing Group Ltd ("BMJ") its licensees and where the relevant Journal is co-owned by BMJ to the co-owners of the Journal, to publish the Work in BMJ Open and any other BMJ products and to exploit all rights, as set out in our licence.

The Submitting Author accepts and understands that any supply made under these terms is made by BMJ to the Submitting Author unless you are acting as an employee on behalf of your employer or a postgraduate student of an affiliated institution which is paying any applicable article publishing charge ("APC") for Open Access articles. Where the Submitting Author wishes to make the Work available on an Open Access basis (and intends to pay the relevant APC), the terms of reuse of such Open Access shall be governed by a Creative Commons licence – details of these licences and which Creative Commons licence will apply to this Work are set out in our licence referred to above.

Acknowledgements: Thank you to Bolaji Coker for the REDCap administration and management. Thank you to Beverley Power (CDH UK) for representing patients, parents and families through your feedback on the study design during the pilot study. Thank you to Xiya Ma and Dylan Goh for helping with the Chinese translation of study documentation.

COLLABORATORS

Principal Investigator: Naomi Wright (King's College London, UK)

Steering Committee: Niyi Ade-Ajayi (King's College Hospital, UK), Adesoji Ademuyiwa (Lagos University Teaching Hospital, Nigeria), Emmanuel Ameh (National Hospital, Abuja, Nigeria), Justine Davies (University of Birmingham, UK), Kokila Lakhoo (University of Oxford and Oxford University Hospitals, UK), Dan Poenaru (McGill University, Montreal, Canada), Nick Sevdalis (King's College London, UK), Emily Smith (Baylor University, Texas, USA), Andy Leather (King's College London, UK)

Writing Committee: Harmony Ubhi (King's College London, UK), Samuel Parker (Imperial College London, UK), Godfrey Sama Philipo (Muhimbili University of Health and Allied Sciences, Tanzania)

Organising Committee: Sadi Abukhalaf (Al Quds University, Palestine), Nana Adofo-Ansong (Mahikeng Provincial Hospital, South Africa), Melika Akhbari (King's College London, UK), Ahmad Alhamid (University of Aleppo, Syria), Osaid H. Alser (University of Oxford, UK), Emrah Aydin (Koc University, Turkey), Yousra-Imane Benaskeur (Universite de Montreal, Canada), Shrouk M. Elghazaly (Assiut University, Egypt), Safa abdal Elrais (University of Tripoli, Libya), Sophia Hashim (University College London, UK), Laura Herrera (Geisel School of Medicine, Dartmouth, USA), Gabriella Hyman (University of Witwatersrand, South Africa), Henang Kwasau (College of Medicine and Allied Health Sciences, University of Sierra Leone), Yang Liu (Children's Hospital, Zhejiang University School of Medicine, China), Bruno Martinez-Leo (Moctezuma Children's Hospital, Mexico), Kelly Naranjo (Columbia University Medical Centre, USA), Ibrahim Nour (Jordan University Hospital, Jordan), Cristiana Riboni (University of Pavia, Italy), Mahmoud Saleh (University of Gezira, Sudan), Hosni Khairy Salem (Cairo University, Egypt), Patricia Shinondo (University Teaching Hospital, Lusaka, Zambia), Marcus Sim (Stepping Hill Hospital, UK), Hannah Thompson (King's College Hospital, UK), Agota Vaitkiene (Vilnius University Hospital Santaros Kliniko, Lithuania), Dominique Vervoort (Harvard Medical School, USA), Isabelle Williams (Cambridge University, UK), Aayenah Yunus (King's College London, UK).

Lead Investigators: Muhammad Amjad Chaudhary, Adnan Ahmed Khan Khattak, Muhammad Bin Amjad (Children's Hospital, PIMS, Islamabad, Pakistan), Marlene Dominguez Anaya (Children's Hospital Manuel A.Villarroel Cochabamba, Bolivia), Samiul Hasan, Sabbir Karim, Ashrarur Rahman Mitul (Dhaka Shishu (Children) Hospital, Bangladesh), Paolo Bragagnini, Segundo Rite (Hospital Universitario Miguel Servent, Zaragoza), Hana Arbab, Lubna Samad, Aqil Soomro (Indus Hospital, Pakistan), Niveshni Maistry (John Radcliffe Hospital, UK), Raed Nael Al-Taher, Ibrahim Rabi Nour, Osama Abdul Kareem Sarhan (Jordan University Hospital, Jordan), Muhammad Arshad, Taimur

For peer review only - http://bmjopen.bmj.com/site/about/guidelines.xhtml

Qureshi, Hina Yousaf (Liaquat National Hospital, Pakistan), Candy SC Choo, Doris Mae Dimatatac, Shireen Anne Nah (KK Women's and Children's Hospital, Singapore), Vijay Anand Ismavel, Ann Miriam, Shajin T (Makunda Christian Leprosy and General Hospital, India), Monica Ivanov, Andreea Serban (Marie Curie Hospital in Bucharest, Romania), Bruno Martinez-Leo (Moctezuma Children's Hospital, Mexico), Eva Blazquez-Gomez, Luis Garcia-Aparicio, Martí Iriondo, Jordi Prat, Xavier Tarrado (Hospital Sant Joan de Deu, Spain), Lars Hagander, Emma Svensson (Skane University Hospital's Pediatric Care Hospital, Lund, Sweden), Alhassan Abdul-Mumin, Dominic Bagbio, Sheila Owusu, Stephen Tabiri (Tamale Teaching Hospital, Ghana), Dayang Anita Abdul Aziz (UKM Medical Centre, Kuala Lumpur, Malaysia).

For peer review only

REFERENCES

1. GBD Child Mortality Collaborators. Global, regional, national, and selected subnational levels of stillbirths, neonatal, infant, and under-5 mortality, 1980-2015: a systematic analysis for the Global Burden of Disease Study 2015. Lancet 2016; 388(10053): 1725-74.
2. Boyle B, Addor MC, Arriola L, et al. Estimating Global Burden of Disease due to congenital anomaly: an analysis of European data. Arch Dis Child Fetal Neonatal Ed 2017.
3. Flores A, Valencia D, Sekkarie A, et al. Building capacity for birth defects surveillance in Africa: Implementation of an intermediate birth defects surveillance workshop. J Glob Health Perspect 2015; 2015.
4. Sitkin NA, Ozgediz D, Donkor P, Farmer DL. Congenital anomalies in low- and middle-income countries: the unborn child of global surgery. World J Surg 2015; 39(1): 36-40.
5. Ozgediz D, Langer M, Kisa P, Poenaru D. Pediatric surgery as an essential component of global child health. Semin Pediatr Surg 2016; 25(1): 3-9.
6. Farmer D, Sitkin N, Lofberg K, Donkor P, Ozgediz D. Surgical Interventions for Congenital Anomalies. In: Debas HT, Donkor P, Gawande A, Jamison DT, Kruk ME, Mock CN, eds. Essential Surgery: Disease Control Priorities, Third Edition (Volume 1). Washington (DC); 2015.
7. Sakonidou S, Ali K, Farmer I, Hickey A, Greenough A. Mortality and short-term morbidity in infants with exomphalos. Pediatr Int 2018.
8. Ameh EA, Seyi-Olajide JO, Sholadoye TT. Neonatal surgical care: a review of the burden, progress and challenges in sub-Saharan Africa. Paediatr Int Child Health 2015; 35(3): 243-51.
9. Ekenze SO, Ajuzieogu OV, Nwomeh BC. Challenges of management and outcome of neonatal surgery in Africa: a systematic review. Pediatr Surg Int 2016; 32(3): 291-9.
10. Wright NJ, Zani A, Ade-Ajayi N. Epidemiology, management and outcome of gastroschisis in Sub-Saharan Africa: Results of an international survey. Afr J Paediatr Surg 2015; 12(1): 1-6.
11. PaedSurg Africa Research Collaboration. Paediatric Surgery across Sub-Saharan Africa: A Multi-Centre Prospective Cohort Study. https://clinicaltrials.gov/ct2/show/NCT03185637 (Accessed 26th January 2019).
12. Bradnock T, Marven S, Owen A, et al. Gastroschisis: one year outcomes from national cohort study. BMJ 2011; 343(d6749).
13. Krishnaswami S, Nwomeh BC, Ameh AE. The pediatric surgery workforce in low- and middle-income countries: problems and priorities. Semin Pediatr Surg 2016; 25(1): 32-42.
14. Okoye MT, Ameh EA, Kushner AL, Nwomeh BC. A pilot survey of pediatric surgical capacity in West Africa. World J Surg 2015; 39(3): 669-76.
15. United Nations. Sustainable Development Goals. 2015. http://www.un.org/sustainabledevelopment/health/ (accessed 27th February 2018).
16. Wright NJ, Anderson JE, Ozgediz D, Farmer DL, Banu T. Addressing paediatric surgical care on World Birth Defects Day. Lancet 2018; 391(10125): 1019.
17. GlobalSurg Collaborative. Mortality of emergency abdominal surgery in high-, middle- and low-income countries. Br J Surg 2016; 103(8): 971-88
18. GlobalSurg Collaborative. Surgical site infection after gastrointestinal surgery in high-income, middle-income, and low-income countries: a prospective, international, multi-centre cohort study. Lancet Infect Dis 2018; 18(5): 516-25.
19. GlobalSurg Collaborative. Laparoscopy in management of appendicitis in high-, middle-, and low-income countries: a multicenter, prospective, cohort study. Surg Endosc 2018.
20. GlobalSurg Collaborative. Determinants of morbidity and mortality following emergency abdominal surgery in children in low-income and middle-income countries. BMJ Glob Health 2016; 1(4): e000091.
21. Bhangu A, Kolias AG, Pinkney T, Hall NJ, Fitzgerald JE. Surgical research collaboratives in the UK. Lancet 2013; 382(9898): 1091-2.
22. Long AM, Bunch KJ, Knight M, Kurinczuk JJ, Losty PD, Baps C. Early population-based outcomes of infants born with congenital diaphragmatic hernia. Arch Dis Child Fetal Neonatal Ed 2018.
23. Ruttenstock E, Wright N, Barrena S, et al. Best oxygenation index on day 1: a reliable marker for outcome and survival in infants with congenital diaphragmatic hernia. Eur J Pediatr Surg 2015; 25(1): 3-8.
24. Burjonrappa S, Crete E, Bouchard S. Comparative outcomes in intestinal atresia: a clinical outcome and pathophysiology analysis. Pediatr Surg Int 2011; 27(4): 437-42.
25. Forrester MB, Merz RD. Structural birth defects associated with omphalocele and gastroschisis, Hawaii, 1986-2001. Congenit Anom (Kyoto) 2008; 48(2): 87-91.

For peer review only

26. Kunz SN, Tieder JS, Whitlock KJ, Jackson C, Avansino JR. Primary fascial closure versus staged closure with silo in patients with gastroschisis: a meta-analysis. J Pediatr Surg 2013; 48(4): 845-57.
27. Ross AR, Eaton S, Zani A, Ade-Ajayi N, Pierro A, Hall NJ. The role of preformed silos in the management of infants with gastroschisis: a systematic review and meta-analysis. Pediatr Surg Int 2015; 31(5): 473-83.
28. Levitt MA, Pena A. Anorectal malformations. Orphanet J Rare Dis 2007; 2: 33.
29. Van der Steeg HJ, Schmiedeke E, Bagolan P, et al. European consensus meeting of ARM-Net members concerning diagnosis and early management of newborns with anorectal malformations. Tech Coloproctol 2015; 19(3): 181-5.
30. Holschneider A, Hutson J, Pena A, et al. Preliminary report on the International Conference for the Development of Standards for the Treatment of Anorectal Malformations. J Pediatr Surg 2005; 40(10): 1521-6.
31. Bradnock TJ, Knight M, Kenny S, Nair M, Walker GM, British Association of Paediatric Surgeons Congenital Anomalies Surveillance S. Hirschsprung's disease in the UK and Ireland: incidence and anomalies. Arch Dis Child 2017; 102(8): 722-7.
32. Ross AR, Hall NJ. Outcome reporting in randomized controlled trials and systematic reviews of gastroschisis treatment: a systematic review. J Pediatr Surg 2016; 51(8): 1385-9.
33. Allin BSR, Hall NJ, Ross AR, et al. Development of a gastroschisis core outcome set. Arch Dis Child Fetal Neonatal Ed 2018.
34. Allin BS, Irvine A, Patni N, Knight M. Variability of outcome reporting in Hirschsprung's Disease and gastroschisis: a systematic review. Sci Rep 2016; 6: 38969.
35. Watanabe S, Suzuki T, Hara F, Yasui T, Uga N, Naoe A. Omphalocele and Gastroschisis in Newborns: Over 16 Years of Experience from a Single Clinic. J Neonatal Surg 2017; 6(2): 27.
36. Tan KB, Tan KH, Chew SK, Yeo GS. Gastroschisis and omphalocele in Singapore: a ten-year series from 1993 to 2002. Singapore Med J 2008; 49(1): 31-6.
37. Schneider A, Blanc S, Bonnard A, et al. Results from the French National Esophageal Atresia register: one-year outcome. Orphanet J Rare Dis 2014; 9: 206.
38. World Bank. World Bank Country and Lending Groups. 2018. https://datahelpdesk.worldbank.org/knowledgebase/articles/906519-world-bank-country-and-lending-groups (Accessed 26th January 2019).

For peer review only

Management and Outcomes of Gastrointestinal Congenital Anomalies in Low-, Middle- and High-Income Countries: Protocol for a Multi-Centre, International, Prospective Cohort Study

SUPPLEMENTARY FILE 1: Condition Specific Data Points

Oesophageal Atresia (OA) +/- Tracheo-Oesophageal Fistula (TOF)

Question	Answers
Type of OA +/- TOF (Gross classification)	A, B, C, D, E<br>A: without a fistula, B: proximal TOF with distal OA, C: distal TOF with proximal OA, D: proximal and distal TOF, E: H-type TOF without OA.
Long or short gap?	Long, Short, Unknown<br>Long gap OA: A gap of 4 vertebral bodies or more. Anatomically cases either have no TOF or a gap of over 4 vertebral bodies following division of the distal fistula making primary repair unfeasible. Short OA: A gap of less than 4 vertebral bodies. Primary anastomosis typically feasible.
Pneumonia at presentation?	Yes: diagnosed clinically, Yes: diagnosed radiologically, Yes: other means of diagnosis, No: patient born in the study centre, No: patient born outside the study centre but no evidence of pneumonia on arrival<br>Pneumonia is defined as lung inflammation typically caused by bacterial or viral infection, in which the air sacs fill with pus and may become solid.
Primary intervention:	TOF ligation, Oesophageal anastomosis, Oesophagostomy, Gastrostomy, Ligation of the distal oesophagus, Gastro-oesophageal disconnection, Foker technique, Fundoplication, Other (please specify), Palliative care<br>Select all that apply.
If the patient had a primary oesophageal anastomosis, was a post-operative oesophagogram undertaken?	Yes, No. (At any stage)
If yes, routine or clinically indicated?	Routine, Clinically indicated
If yes, when?	Number of days after primary surgery
If yes, what was the result?	Leak, No leak
For patients diagnosed with a leak radiologically, was it associated with clinical symptoms?	Yes, No
Time to first oral feed post-operatively	In days. Please include the day of surgery and the first day of oral feeds in the calculation. Enter 0 if oral feeds were not commenced within 30-days of primary intervention. Do not include other types of enteral feeding such as nasogastric or gastrostomy feeding.
Time to full oral feeds	In days (enter 0 if the patient died before reaching full oral feeds or 30 if the patient had not reached full oral feeds at 30-days post primary intervention). Do not include other types of enteral feeding such as nasogastric or gastrostomy feeding.
For patient's not receiving a primary oesophageal anastomosis, at what age is definitive surgery planned?	In months (enter unknown if not planned or enter not applicable if primary anastomosis was undertaken).
For patient's not receiving a primary oesophageal anastomosis, what is the future planned procedure?	Gap assessment, Primary oesophageal anastomosis if possible, Gastric pull-up, Jejunal interposition, Colonic interposition, Not applicable: primary anastomosis undertaken, Other, Unknown. Select all that apply. If other, please specify.
If the patient had surgery, what was the approach?	Thoracotomy muscle cutting, Thoracotomy muscle splitting, Thoracoscopy, Laparotomy, Laparoscopy, Limited local incision, Other.<br>During primary surgery. If other, please specify.
If thoracoscopic or laparoscopic, was the surgery converted to open?	Yes, No
Did the patient have a condition specific complication within 30-days of primary intervention?	Pneumonia, Mediastinitis, Pneumothorax, Chylothorax, Haemothorax, Anastomotic leak, Anastomotic stricture, Recurrent TOF, Other, None<br>Select all that apply. If other, please specify.
Did the patient have tracheomalacia?	Yes: diagnosed clinically, Yes: diagnosed on bronchoscopy, Yes: diagnosed on CT, Yes: diagnosed on bronchogram, Yes: other method of diagnosis, No
If yes, was an intervention undertaken? If other, please specify	Yes: aortopexy, Yes: tracheostomy, Yes: tracheal stent, Yes: supportive management (oxygen +/- ventilation) only, Yes: other treatment, No

Congenital Diaphragmatic Hernia (CDH)

Question	Answers
Type of CDH.	Left posteriolateral (Bochdalek), Right posteriolateral (Bochdalek), Bilateral posteriolateral (Bochdalek), Central, Anterior (Morgagni), Other. If other, please specify.
Type of Bochdalek CDH (CDH Study Group Classification)	A, B, C, D, Other (specify), Unknown.<br>Defect A: smallest defect, usually "intramuscular" defect with >90% of the hemi-diaphragm present; this defect involves < 10% of the circumference of the chest wall. Defect B: 50-75% hemi-diaphragm present; this defect involves < 50% of

For peer review only - http://bmjopen.bmj.com/site/about/guidelines.xhtml

 <br><br><br><br><br><br><br>If bilateral, what was the type of Bochdalek hernia on the left?<br>If bilateral, what was the type of Bochdalek hernia on the right?	the chest wall. Defect C: < 50% hemi-diaphragm present; this defect involves >50% of the chest wall. Defect D: largest defect (previously known as "agenesis"); complete or near complete absence of the diaphragm with < 10% hemi-diaphragm present; this defect involves >90% of the chest wall. Surgically, it is an absent posterior rim beyond the spine, absent posterior-lateral rim, and an anterior/anterior-medial rim which is miniscule. As it is truly unusual to have zero tissue at all, this is the CDHSG member consensus. "D" defects should all require a patch (or muscle flap) for repair.<br>A, B, C, D, Other, Unknown<br>If other, please specify.<br>A, B, C, D, Other, Unknown<br>If other, please specify.
If antenatally diagnosed, what was the lung-to-head ratio (LHR)?	Enter zero if not undertaken/ not known.
Was foetal tracheal occlusion (FETO) undertaken?<br>If yes, at what gestational age was it inserted?<br>If yes, at was gestational age was it removed?	Yes, No<br>__________, unknown.<br>__________, at birth, unknown.
Liver position?	Chest, Abdomen, Unknown
Did the patient have pulmonary hypertension (at any stage)?<br><br><br><br><br><br><br><br><br>If yes, treatment given? If other, please specify.	Yes: diagnosed clinically, Yes: diagnosis confirmed on echocardiography, Yes: other method of confirming diagnosis, No, Unknown<br>Persistent pulmonary hypertension of the newborn (PPHN) is defined as the failure of the normal circulatory transition that occurs after birth. It is a syndrome characterised by marked pulmonary hypertension that causes hypoxemia secondary to right-to-left extrapulmonary shunting of deoxygenated blood. It should be suspected whenever the level of hypoxemia is out of proportion to the level of pulmonary disease. Echocardiography plays a major role in screening and assisting in making the diagnosis of PPHN.<br>Nitric oxide, Prostacyclin, Alprostadil, Milrinone, Other, None: not required, None: required but not available.
Did the patient receive extracorporeal membrane oxygenation (ECMO)?<br>If yes, for how long?	Yes, No<br><br>In days. Include the day the patient went onto ECMO and the day they were taken off in the calculation.
Primary intervention<br><br>If patch repair, material used?<br><br>Other procedures undertaken at the same time?<br><br><br>Surgical approach:<br>If laparoscopic or thoracoscopic, was the surgery converted to open?<br>Condition specific complication within 30-days of primary surgery?	Primary repair (absorbable sutures), Primary repair (non-absorbable sutures), Patch repair, Palliation, Discharged with planned elective repair, Other<br>Permacol, PTFE, Alloderm, Dacron, Mesh plug, Muscle flap, Surgisis, Other. If other, please specify.<br>Chest drain insertion, Abdominal wall patch, Fundoplication, Correction of malrotation, Appendicectomy, Other (specify), None<br>Select all that apply. If other, please specify.<br>Laparotomy, Laparoscopy, Thoracotomy, Thoracoscopy, Other (please specify)<br>Yes/No.<br><br>Air leak (not just redundant space in the pleural cavity which is common), Chylothorax, Recurrence, Adhesional obstruction, Other, None. Select all that apply. If other, please specify.

Intestinal Atresia

Question	Answers
Type of intestinal atresia	Duodenal, Jejuno-ileal, Colonic
Classification of duodenal or colonic atresia<br><br><br><br>Classification of jejuno-ileal atresia	1,2,3,4<br>1) intraluminal web with continuity of the muscular layer, 2) atretic segment without a mesenteric defect, 3) atretic segment with mesenteric defect, 4) multiple atresias = string of sausages appearance.<br>1,2,3a,3b,4<br>1) intraluminal web with continuity of the muscular layer, 2) atretic segment without a mesenteric defect, 3a) atretic segment with mesenteric defect, 3b) apple-peel (bowel wrapped around a single artery), 4) multiple atresias = string of sausages appearance.
Primary intervention for duodenal atresia:<br><br>Surgical approach<br>Conversion to open procedure?<br>Type of anastomosis<br>Primary intervention for jejuno-ileal and colonic atresia:<br><br><br>If bowel was excised, what was the total length of bowel excised?<br>Surgical approach:<br>Conversion to open procedure?<br>Was the distal bowel flushed to check for patency?	Duodenoduodenostomy, Duodenojenunostomy, Web excision only, Palliation, Other. If other, please specify.<br>Laparotomy, Laparoscopy, Endoscopy, Other<br>Yes/ No<br>Kimura's diamond shape, Side-to-side, End-to-end<br>Primary anastomosis, Bowel resection, Division of web only, Loop stoma, Divided stoma, Bishop-Koop stoma, Santulli stoma, Palliation, Other. Select all that apply.<br>In centimetres (cm). Enter 0 if unknown<br><br>Laparotomy, Laparoscopy, Endoscopy, Other<br>Yes, No<br>Yes, No

For peer review only

If the patient underwent surgery, did they have a condition specific complication within 30-days of primary intervention?	Anastomotic leak, Anastomotic stenosis, Short-gut, Missed additional atresia, Adhesive bowel obstruction, Stoma prolapse, Stoma retraction, Parastomal hernia, Parastomal skin breakdown, Other. If other, please specify. Select all that apply. For the purposes of this study short gut is defined as more than 50% of the small intestine excised (when short bowel syndrome can occur).

Gastroschisis

Question	Answers
Type of gastroschisis	Simple, Complex: associated with atresia, Complex: associated with necrosis, Complex: associated with perforation, Complex: associated with closing gastroschisis. Select all that apply.
Primary intervention:	Primary closure in the operating room (OR), Primary closure at the cotside (Bianchi technique), Staged closure using a preformed silo, Staged closure using an Alexis Wound Retractor and Protector, Staged closure using a surgical silo (including improvised silo), Other method, No intervention undertaken. If other, please specify.
Method of defect closure:	Fascia and skin closed with sutures, Just skin closed with sutures: fascia left open, Umbilical cord sutured over the defect: fascia left open, Sutureless closure with skin edges opposed and dressing applied, Dressing applied: defect left open to close by secondary intention, Other, Patient died before the defect was closed. If other, please specify.
On what day following admission was abdominal wall closure achieved?	In days. Please include the first day of admission and the day of closure in the calculation. For example, for a neonate admitted with gastroschisis on 2nd October who had the defect closed on 4th October, please insert 3 days.
Did the neonate have any of these complications within 30-days of primary intervention?	Ischemic bowel, Abdominal compartment syndrome (ACS), Necrotising enterocolitis, None of these Select all that apply. ACS is defined as respiratory insufficiency secondary to compromised tidal volumes, decreased urine output caused by falling renal perfusion or any other organ dysfunction caused by increased intra-abdominal pressure.
If the patient has ACS, was the abdomen re-opened?	Yes/ No

Exomphalos

Question	Answers
Type of Exomphalos?	Major, Minor Major: >50% of the liver in the exomphalos sac and abdominal wall defect >5cm. Minor: Infants with defects less than 5cm.
Hypoglycaemic on arrival?	Yes, No, Blood glucose not measured Hypoglycaemia is defined as a blood glucose level below 4 mmol/L (72mg/dL).
Primary intervention	Primary operative closure, Staged closure, Conservative management
If the patient had a staged closure, what was the time from primary intervention to closure?	In days. Please include the day of the primary intervention and the day of closure in the calculation. Enter 30 if still not closed at 30-days after primary intervention.
If conservative management, was a topical treatment applied to the exomphalos sac?	Yes: silver sulfadiazine, Yes: betadine, Yes: honey, Yes: merbromide tannage, Yes: other, no. If other, please specify.
If conservative management was undertaken, what is the plan for future management?	No further surgery planned, Delayed closure at this hospital, Delayed closure at another hospital, Other. If other, please specify.
Did the patient have a ruptured sac?	Yes, No

Anorectal Malformation (ARM)

Question	Answers
Type of anorectal malformation (Krickenbeck classification)	Low ARM: Perineal (cutaneous) fistula, High ARM: Rectourethral fistula (bulbar), High ARM: Rectourethral fistula (prostatic), High ARM: Rectovesical fistula, High ARM: Vestibular fistula, High ARM: Cloaca, High ARM: No fistula, High ARM: Type unknown at present, Rare variant: Pouch colon, Rare variant: Rectal atresia/ stenosis, Rare variant: Rectovaginal fistula, Rare variant: H fistula, Other
Did the neonate have pre-operative bowel perforation?	Yes, No
What was the primary intervention undertaken?	Fistula dilation: no surgery, Loop sigmoid colostomy, Divided sigmoid colostomy, Loop transverse colostomy, Divided transverse colostomy, Other stoma, Anoplasty, Posterior sagittal anorectoplasty (PSARP), Abdominosacroperineal pull-through, Abdominoperineal pull-through, Laparoscopic-assisted pull-through, Palliative care, Other. If other, please specify. Select all that apply.
If primary anorectal reconstruction was undertaken, was a Peña stimulator or equivalent used to identify the position of the muscle complex intra-operatively?	Yes, no: equipment was not available, no: the equipment was available but not used. Peña stimulator: Muscle locating stimulator commonly used to identify the anal sphincter muscles whilst undertaking a PSARP for patients with ARM.
Did the patient have any of the following complications within 30-days of surgery?<br>- Electrolyte disturbance<br>- High output stoma (over 20mls/kg/day)	For each of the below answer: Yes, No, Not applicable

For peer review only - http://bmjopen.bmj.com/site/about/guidelines.xhtml

- Stoma prolapse/ retraction/ herniation<br>- Peri-stoma skin breakdown (or perianal if primary reconstructive surgery undertaken without a covering stoma)<br>- Anal stenosis in those undergoing primary anorectal reconstruction without covering stoma.	
What is the plan for future management?	No further operative management, Anoplasty/ pull-through planned at your hospital, Anoplasty/ pull-through planned at another hospital, Stoma closure planned at your hospital, Stoma closure planned at another hospital, Other<br>Please tick all that apply. If other, please specify.

Hirschsprung's Disease

Question	Answers
Time to first passage of meconium after birth	Less than 24 hours, 24-48 hours, Over 48 hours, Unknown
Features at presentation:	Abdominal distension, Bilious vomiting, Non-bilious vomiting, Poor feeding, Suspected enterocolitis, Perforation, Other. Select all that apply.
Source of diagnosis of Hirschsprung's disease	Genetic, Mucosal biopsy, Full thickness biopsy, Anorectal manometry, Barium enema, Not confirmed: suspected only, Other.
If on biopsy, what was the method of histology staining.	Hemotoxilin and Eosin (H&E), Acetylcholinesterase, Calretinin, Other.<br>Select all that apply. If other, please specify.
Length of aganglionosis:	Rectal, Sigmoid, Descending colon, Transverse colon, Ascending colon, Small bowel, Unknown at present
Primary intervention	Conservative: no treatment, Conservative: digital stimulation and laxatives, Conservative: regular rectal washouts/ enemas, Failed conservative management followed by a stoma during the same hospital admission, Primary stoma (with or without pre-operative washouts or enemas prior to a planned stoma placement), Primary pull-through (Swenson), Primary pull-through (Duhamel), Primary pull-through (Soave), Primary pull-through (Other), Transanal posterior anorectal myectomy, Palliative care, Other.
If primary pull-through was undertaken, did the patient have a covering stoma?	Yes, No
Was it laparoscopic assisted?	Yes, No
Did the patient have any condition specific complications within 30-days of primary intervention?	Hirschsprung's associated enterocolitis (HAEC), Electrolyte disturbance, High stoma output (over 20mls/kg/day), Stoma prolapse/ retraction/ herniation, Peri-stoma skin breakdown (or perianal if primary pull-through was undertaken without a covering stoma), Anal stenosis, Post-operative obstruction, Anastomotic leak (if primary pull-through was undertaken without a covering stoma), Other<br>HAEC is defined as inflammation of the small and or large bowel in patient's born with Hirschsprung's disease. If the patient was managed conservatively, please tick if they developed enterocolitis within 30-days of presentation. Select all that apply.
What is the plan for future management?	No further surgery planned, Anorectal pull-through at your hospital, Anorectal pull-through at a different hospital, Stoma closure, Other, Unknown

For peer review only

Management and Outcomes of Gastrointestinal Congenital Anomalies in Low-, Middle- and High-Income Countries: Protocol for a Multi-Centre, International, Prospective Cohort Study

SUPPLEMENTARY FILE 2

Collaborator Survey: Resources and Facilities for Neonatal and Paediatric Surgery

Question	Answers
Title	Professor, Dr, Mr, Mrs, Miss, Ms, Other
Surname	
First Name	
Professional Position	Professor, Consultant or attending, Registrar or resident, Intern/ house officer/ senior house officer, Medical Student, Nurse, Other
Are you the study lead at your centre?	Yes, No<br>Answers provided by the study lead will be used as the 'Gold Standard' to which the other survey will be compared (this will remain anonymous).
Speciality	General Surgery (adult and paediatric), Paediatric Surgery, Anaesthetics, Paediatrics, Neonatology, Nursing, Not specialised yet, Other
Full name of institution:	
Address of institution:	
Country	Afghanistan, Albania, Algeria, Andorra, Angola, Antigua and Barbuda, Argentina, Armenia, Aruba (Kingdom of the Netherlands), Australia, Austria, Azerbaijan, Bahamas, Bahrain, Bangladesh, Barbados, Belarus, Belgium, Belize, Benin, Bhutan, Bolivia, Bosnia and Herzegovina, Botswana, Brazil, Brunei Darussalam, Bulgaria, Burkina Faso, Burundi, Cambodia, Cameroon, Canada, Cape Verde, Central African Republic, Chad, Chile, China, Colombia, Comoros, Congo, Costa Rica, Cote d'Ivoire, Croatia, Cuba, Curacao (Kingdom of the Netherlands), Cyprus, Czech Republic, Democratic Peoples Republic of Korea, Democratic Republic of the Congo, Denmark, Djibouti, Dominica, Dominican Republic, Ecuador, Egypt, El Salvador, Equatorial Guinea, Eritrea, Estonia, Eswatini (Swaziland), Ethiopia, Fiji, Finland, France, Gabon, Gambia, Georgia, Germany, Ghana, Greece, Grenada, Guatemala, Guinea, Guinea-Bissau, Guyana, Haiti, Holy See (Vatican City State), Honduras, Hong Kong (China), Hungary, Iceland, India, Indonesia, Iran (Islamic Republic of), Iraq, Ireland, Israel, Italy, Jamaica, Japan, Jordan, Kazakhstan, Kenya, Kiribati, Korea (Republic of), , Kuwait, Kyrgyzstan, Lao People's Democratic Republic, Latvia, Lebanon, Lesotho, Liberia, Libya, Liechtenstein, Lithuania, Luxembourg, Macau (China), Macedonia (the former Yugoslav Republic of), Madagascar, Malawi, Malaysia, Maldives, Mali, Malta, Marshall Islands, Mauritania, Mauritius, Mexico, Micronesia (Federated States of), Moldova (Republic of), Monaco, Mongolia, Montenegro, Morocco, Mozambique, Myanmar, Namibia, Nauru, Nepal, Netherlands, New Zealand, Nicaragua, Niger, Nigeria, Niue, Norway, Oman, Pakistan, Palau, Palestinian Territories, Panama, Papua New Guinea, Paraguay, Peru, Philippines, Poland, Portugal, Qatar, Republic of Kosovo, Reunion Island, Romania, Russian Federation, Rwanda, Saint Kitts and Nevis, Saint Lucia, Saint Vincent and the Grenadines, Samoa, San Marino, Sao Tome and Principe, Saudi Arabia, Senegal, Serbia, Seychelles, Sierra Leone, Singapore, Sint Maarten (Kingdom of the Netherlands), Slovakia, Slovenia, Solomon Islands, Somalia, Somaliland, South Africa, South Sudan, Spain, Sri Lanka, Sudan, Suriname, Swaziland (See Eswatini), Sweden, Switzerland, Syrian Arab Republic, Taiwan, Tajikistan, Tanzania (United Republic of), Thailand, Timor-Leste, Togo, Tonga, Trinidad and Tobago, Tunisia, Turkey, Turkmenistan, Tuvalu, Uganda, Ukraine, United Arab Emirates, United Kingdom, Uruguay, USA, Uzbekistan, Vanuatu, Venezuela, Viet Nam, Yemen, Zambia, Zimbabwe
Type of institution (WHO classification)	- **Specialised children's hospital** (Provides highly specialised care dedicated to children)<br>- **Referral hospital** (WHO defined tertiary healthcare. Includes academic, university, teaching, national, central and specialised mission hospitals. Can provide specialised surgical services)<br>- **District hospital** (WHO defined secondary healthcare. Includes provincial, general, general mission or regional hospitals. Has general anaesthesia and can provide general surgical care)<br>- **Health centre** (WHO defined primary healthcare. No general anaesthesia, can do minor local procedures, wound management, triage and referral).

For peer review only - http://bmjopen.bmj.com/site/about/guidelines.xhtml

Institutional classification	Government, Non-government
Institutional financial classification	Not for profit, For profit
Population served by your institution (in millions, including children and adults)	
**Personnel**	
Number of Consultant Paediatric Surgeons undertaking neonatal surgery at your institution:	___________________ (Excluding trainees)
Number of Consultant General Surgeons (covering adults and children) undertaking neonatal surgery independently at your institution:	___________________ (Excluding trainees)
Number of medical officers or other non-surgeon healthcare professionals undertaking neonatal surgery independently at your institution	___________________ (Without a consultant surgeon present at the time of surgery)
**Infrastructure**<br>Please state whether the following facilities are available at your institution when required:	
Running Water	Always, Sometimes, Never
Electricity	Always, Sometimes, Never
Electricity generator back-up	Always, Sometimes, Never
Laboratory for biochemistry	Always, Sometimes, Never
Laboratory for haematology	Always, Sometimes, Never
Blood bank	Always, Sometimes, Never
Sterile gloves and gown	Always, Sometimes, Never
Autoclave for sterilising surgical equipment	Always, Sometimes, Never
Neonatal ventilation outside the operating room	Always, Sometimes, Never
Paediatric ventilation outside the operating room	Always, Sometimes, Never
Neonatal intensive care unit for surgical neonates pre and post operatively (including if a stoma is present)	Always, Sometimes, Never
Paediatric intensive care unit for surgical paediatric patients pre and post operatively if required	Always, Sometimes, Never
Parenteral nutrition for neonates	Always, Sometimes, Never
Parenteral nutrition for adults and older children	Always, Sometimes, Never
Extracorporeal membrane oxygenation (ECMO)	Always, Sometimes, Never
Peña stimulator or equivalent device to identify the muscle complex during anorectal reconstruction	Always, Sometimes, Never
Suction rectal biopsy gun to investigate for Hirschsprung's disease	Always, Sometimes, Never
**Procedures**<br>Please state whether the following procedures are available at your institution when clinically appropriate/ required:	
Neonatal laparotomy	Always, Sometimes, Never
Neonatal laparoscopy	Always, Sometimes, Never
Neonatal thoracotomy	Always, Sometimes, Never
Neonatal thoracoscopy	Always, Sometimes, Never
Neonatal central line insertion	Always, Sometimes, Never
Paediatric central line insertion	Always, Sometimes, Never
Umbilical vein catheterisation	Always, Sometimes, Never
Bedside primary reduction and closure of gastroschisis (Bianchi technique)	Always, Sometimes, Never
Preformed silo application, reduction and closure of gastroschisis	Always, Sometimes, Never
Surgical silo application, reduction and closure of gastroschisis	Always, Sometimes, Never
Primary closure of gastroschisis in the operating room	Always, Sometimes, Never
Sigmoid colostomy	Always, Sometimes, Never
Posterior Sagittal Anorectoplasty (PSARP) for anorectal malformation	Always, Sometimes, Never
Foetal tracheal occlusion (FETO) for CDH	Always, Sometimes, Never
Pull-through for Hirschsprung's disease	Always, Sometimes, Never
**Anaesthesia and resuscitation**<br>Please state whether the following facilities are available at your institution when required:	
Neonatal bag, valve and mask	Always, Sometimes, Never
Paediatric bag, valve and mask	Always, Sometimes, Never
Bottled oxygen	Always, Sometimes, Never
Piped oxygen	Always, Sometimes, Never
Oxygen saturation monitor	Always, Sometimes, Never
Apnoea monitor	Always, Sometimes, Never
Multi-parameter intra-operative monitoring	Always, Sometimes, Never
Anaesthetic machine for neonates	Always, Sometimes, Never
Anaesthetic machine for children	Always, Sometimes, Never
Ketamine anaesthesia for neonates	Always, Sometimes, Never
Ketamine anaesthesia for children	Always, Sometimes, Never
Spinal/ caudal anaesthesia for neonates	Always, Sometimes, Never
Spinal/ caudal anaesthesia for children	Always, Sometimes, Never

For peer review only - http://bmjopen.bmj.com/site/about/guidelines.xhtml

Anaesthetic doctor competent to perform neonatal anaesthesia	Always, Sometimes, Never
Anaesthetic doctor competent to perform paediatric anaesthesia	Always, Sometimes, Never
Anaesthetic nurse competent to perform neonatal anaesthesia	Always, Sometimes, Never
Anaesthetic nurse competent to perform paediatric anaesthesia	Always, Sometimes, Never
Does your country have at least one specialised children's hospital that can provide neonatal and paediatric surgery?	Yes, No
Any other comments?	

For peer review only

Management and Outcomes of Gastrointestinal Congenital Anomalies in Low-, Middle- and High-Income Countries: Protocol for a Multi-Centre, International, Prospective Cohort Study

SUPPLEMENTARY FILE 3: Study approval details of participating centres

Number	Country	Institution Name	Ethical/Audit Review Board Name	Approval Type	Approval Reference Number
**Low-Income Countries (LICs)**					
1	Afghanistan	Ataturk National Children's Hospital	Islamic Republic of Afghanistan Ministry of Public Health, Afghanistan National Public Health Institute, Institutional Review Board	Local (Ethical)	444738
2	Afghanistan	French Medical Institute for Mothers and Children (FMIC)	Institutional Review Board	Local (Ethical)	-
3	Afghanistan	Ghalib University Hospital	Ministry of Higher Education, Ghalib Cadre Hospital	Local (Ethical)	-
4	Afghanistan	Ghor Provincial Hospital	EPHS Directorate, GD of Curative Medicine, Ministry of Public Health	Local (Ethical)	-
5	Afghanistan	Indra Ghandi Institute of Child Health (IGICH)	Institutional Review Board	Local (Ethical)	-
6	Afghanistan	Irena Salimi Children Hospital (ISCH)	Institutional Review Board	Local (Ethical)	-
7	Burundi	Bujumbura, Kamenge Military Hospital	Head of Department Surgery	Local (Departmental)	-
8	Burundi	District Hospital Kibimba, Gitega	Medical Director of Kibimba Hospital	Local (Hospital)	4000342958
9	Burundi	Gitega Hospital Mutoyi	Hospital Director	Local (Hospital)	-
10	Burundi	Kibuye Hope Hospital, Hope Africa University, Gitega	Head of Surgery and Ethics Team	Local (Ethical and Hospital)	-
11	Burundi	King Khaled Teaching Hospital	Medical Director General	Local (Hospital)	2019|DECHUK.359|11.5
12	Burundi	Teaching Hospital University of Kamenge	Medical Director General	Local (Hospital)	2019|DECHUK.271|11.5
13	Burundi	Van Norman Clinic Bjumbura	Medical Director of Kibimba Hospital	Local (Hospital)	-
14	Democratic Republic of Congo	HEAL Africa Hospital, Goma	Institutional Review Board at HEAL Africa	Local (Ethical)	-
15	Ethiopia	Addis Ababa University	Deputy Head of Department of Surgery	Local (Departmental)	-
16	Gambia	Edward Francis Small Teaching Hospital, Banjul	Chair of Gambia Government/MRCG Joint Ethics Committee	Local (Ethical)	-
17	Gambia	Kanifing General Hospital, Kanifing	Gambia Government / MRCG Joint Ethics Committee	Regional (Ethical)	-
18	Nepal	Annapurna Children and Women's Hospital	Medical Director	Local (Hospital)	-
19	Niger	National Hospital Lamorde, Niamey	Hospital Director General	Local (Hospital)	000306
20	Rwanda	Centre Hospitalier Universitaire de Butare	College of Medicine and Health Science Institutional Review Board	Local (Ethical)	301/CMHS IRB/2018
21	Rwanda	Rwanda Military Hospital	College of Medicine and Health Science Institutional Review Board	Local (Ethical)	301/CMHS IRB/2018
22	Rwanda	University Teaching Hospital of Kigali	College of Medicine and Health Science Institutional Review Board	Local (Ethical)	301/CMHS IRB/2018
23	Somaliland	Hargeisa Group Hospital	Director of Hargeisa Group Hospital	Local (Hospital)	HGH/800/0.1/18
24	Tanzania	Kilimanjaro Christian Medical Centre	Executive Director	Local (Hospital)	-
25	Zimbabwe	Mpilo Hospital	Clinical Director of Mpilo Central Hospital	Local (Hospital)	-
**TOTAL LICs:** 25					

For peer review only - http://bmjopen.bmj.com/site/about/guidelines.xhtml

Middle-Income Countries (MICs)					
26	Algeria	Hôpital Mère-Enfant EHS EL EULMA	Institutional Ethical Committee of Ferhat ABBAS University SETIF	Local (Ethical)	-
27	Algeria	Hospital Chu Tizi-ouzou	Ethical Committee of the University Mouloud Maameri de Tizi-ouzou	Local (Ethical)	-
28	Algeria	Le Nefissa Hamoud University Hospital	Institutional Ethics Comittee	Local (Ethical)	-
29	Angola	Pediatric Hospital David Bernardino	Hospital Director	Local (Hospital)	55/09/DIRPED/HPDB/018
30	Argentina	Children's Hospital of Santa Fe	Associate Director of Teaching and Research	Local (Hospital)	-
31	Argentina	Clinica Pueyrredon	Medical Director	Local (Hospital)	-
32	Argentina	Dr Humberto Notti Pediatric Hospital of Mendoza	Executive Director	Local (Hospital)	3542-D-2018-04238-E-0-6
33	Argentina	Fundación Dr JR Villavicencio	Clinical Investigation Ethical Committee	Local (Ethical)	-
34	Argentina	Fundacion Hospitalaria	Chief of Department of Paediatric Surgery	Local (Departmental)	-
35	Argentina	Hospital de Niños Victor J. Vilela, Rosario, Santa Fe	President of the Ethics Committee in Research	Local (Ethical)	-
36	Argentina	Hospital Escuela Eva Perón, Rosario	Medical Director	Local (Hospital)	30630225
37	Argentina	Hospital Italiano de Buenos Aires, Buenos Aires	Ethical Committee	Local (Ethical)	139/DGDOIN/14 Anexo 1
38	Argentina	Hospital Provincial de Rosario	Research Ethics Committee (CEI)	Local (Ethical)	-
39	Argentina	Hospital Público Materno Infantil	President of the Advisory Commission on Teaching and Investigation	Local (Hospital)	-
40	Argentina	Hospital Rawson, San Juan	Ethics committee in research	Local (Ethical)	33/18
41	Argentina	Maternidad Martin, Rosario, Santa Fe	President of the Ethics Committee in Research	Local (Ethical)	-
42	Argentina	Maternity Clinic Colón	Teaching and Research committee	Local (Hospital)	-
43	Argentina	Materno Provincial	Health Minister, Training, Teaching and Investigation Committee	Local (Hospital)	-
44	Argentina	Maternoneonatal, Córdoba	Surgical Director	Local (Departmental)	-
45	Argentina	Pediatric Hospital of Santiago del Estero	Deputy Medical Director	Local (Hospital)	-
46	Argentina	Zona Norte Children Hospital	Medical Director	Local (Hospital)	-
47	Bangladesh	Bangabandhu Sheik Mujib Medical University (BSMMU), Dhaka	Chairman, Department of Paediatric Surgery	Local (Departmental)	-
48	Bangladesh	Dhaka Medical College Hospital	Ethical Committee	Local (Ethical)	MEU-DMC/ECC/2018/249
49	Bangladesh	Dhaka Shishu Hospital	Hospital Authority	Local (Hospital)	-
50	Belarus	Republican Scientific Practical Centre of Pediatric Surgery, Minsk	Ethical Council	Local (Ethical)	1-18/394
51	Bolivia	Children's Hospital Manuel A.Villarroel Cochabamba	Medical Director	Local (Hospital)	-
52	Bolivia	Hospital Hernandez Vera, Santa Cruz	Medical Director	Local (Hospital)	-
53	Bolivia	Hospital San Juan de Dios, Tarija	Medical Director	Local (Hospital)	-
54	Bolivia	Mario Ortiz Children's Hospital Suarez, Santa Cruz	Medical Director	Local (Hospital)	-
55	Bolivia	Women's Hospital Percy Boland Rodriguez Santa Cruz	Medical Director	Local (Hospital)	-
56	Bosnia and Herzegovnia	Clinic of Paediatric Surgery, Clinical Centre University of Sarajevo, Sarajevo	Ethical Committee	Local (Ethical)	03-02-47488 (Protocol: 0602-40400)
57	Brazil	Associação de Ensino Superior de Nova Iguaçu	National Ethical Committee	National (Ethical)	3.130.042

For peer review only - http://bmjopen.bmj.com/site/about/guidelines.xhtml

58	Brazil	Associação Hospitalar de Prot Infancia Dr. Raul Carneiro	National Ethical Committee	National (Ethical)	3.130.042
59	Brazil	Botucatu Medical School, State of Sao Paulo University	National Ethical Committee	National (Ethical)	3.130.042
60	Brazil	Centro de Ensino São Lucas Ltda/ RO	National Ethical Committee	National (Ethical)	3.130.042
61	Brazil	Clinical Hospital from Federal University of Uberlandia	National Ethical Committee	National (Ethical)	3.130.042
62	Brazil	Federal University of Minas Gerais	National Ethical Committee	National (Ethical)	3.130.042
63	Brazil	Federal University of Parana	National Ethical Committee	National (Ethical)	3.130.042
64	Brazil	Fundação Facudade Regional de Medicina S J Rio Preto	National Ethical Committee	National (Ethical)	3.130.042
65	Brazil	Fundação Hospitalar Blumenau: Hospital Santo Antoni	National Ethical Committee	National (Ethical)	3.130.042
66	Brazil	Hospital da Criança Santo Antônio – Santa Casa/RS	National Ethical Committee	National (Ethical)	3.130.042
67	Brazil	Hospital da Luz/ São Paulo – SP	National Ethical Committee	National (Ethical)	3.130.042
68	Brazil	Hospital de Base Dr. Ary Pinheiro, Porto Velho	National Ethical Committee	National (Ethical)	3.130.042
69	Brazil	Hospital do Rocio	National Ethical Committee	National (Ethical)	3.130.042
70	Brazil	Hospital Federal de Bonsucesso	National Ethical Committee	National (Ethical)	3.130.042
71	Brazil	Hospital Infantil Jona de Gusmão/ SES – SC	National Ethical Committee	National (Ethical)	3.130.042
72	Brazil	Hospital Jorge Valente/ Salvador - BA	National Ethical Committee	National (Ethical)	3.130.042
73	Brazil	Hospital Materno Infantil de Brasília – HMIB	National Ethical Committee	National (Ethical)	3.130.042
74	Brazil	Hospital Nossa Senhora da Conceição SA	National Ethical Committee	National (Ethical)	3.130.042
75	Brazil	Hospital Santo Antônio Blumenau Santa Catarina	National Ethical Committee	National (Ethical)	3.130.042
76	Brazil	Hospital Universitário de Santa Maria	National Ethical Committee	National (Ethical)	3.130.042
77	Brazil	Hospital Universitário do Oeste do Paraná	National Ethical Committee	National (Ethical)	3.130.042
78	Brazil	Hospitalar Municipal do M'boi Mirim/ São Paulo - SP	National Ethical Committee	National (Ethical)	3.130.042
79	Brazil	Instituto de Medicina Integral Professor Fernando Figueira	National Ethical Committee	National (Ethical)	3.130.042
80	Brazil	Instituto Fernandes Figueira, Rio de Janeiro	National Ethical Committee	National (Ethical)	3.130.042
81	Brazil	Irmandade da Santa Casa de Misericórdia de Santos	National Ethical Committee	National (Ethical)	3.130.042
82	Brazil	Irmandade Nossa Senhora das Merces de Montes Claros	National Ethical Committee	National (Ethical)	3.130.042
83	Brazil	Instituto Fernandes Figueira, Rio de Janeiro	National Ethical Committee	National (Ethical)	3.130.042
84	Brazil	SPDM – Associação Paulista para o Desenvolvimento da Medicina	National Ethical Committee	National (Ethical)	3.130.042
85	Brazil	Universidade Estadual de Montes Claros - UNIMONTES	National Ethical Committee	National (Ethical)	3.130.042
86	Brazil	Universidade Federal de São Paulo – UNIFESP/EPM	National Ethical Committee	National (Ethical)	3.130.042
87	Brazil	University Hospital, Porto Alegre	National Ethical Committee	National (Ethical)	3.130.042
88	Cameroon	Mbingo Hospital in North West Cameroon	Institutional Review Board	Local (Ethical)	-
89	China	Bayi Children's Hospital	Research Ethics Criteria	Local (Ethical)	-
90	China	Children's Hospital of Shanghai	In Chinese script	Local (Ethical)	-

For peer review only - http://bmjopen.bmj.com/site/about/guidelines.xhtml

For peer review only

91	China	Children's Hospital of Soochow University	President of the Seventh Medical Center of PLA General Hospital	Local (Ethical)	-
92	China	Children's Hospital, Zhejiang University School of Medicine, Binjiang District, Hangzhou	Hospital President and Ethics Committee	Local (Ethical)	-
93	China	First Affiliated Hospital of Xinjiang Medical University	Research Ethics Criteria	Local (Ethical)	-
94	China	Hunan Children's Hospital	In Chinese script	Local (Ethical)	-
95	China	Jinhua Municipal Central Hospital	Research Ethics Committee	Local (Ethical)	-
96	China	Kunming Children's Hospital	Medical Ethics Committee	Local (Ethical)	-
97	China	Nanjing Children's Hospital	Research Ethics Criteria	Local (Ethical)	-
98	China	Ningbo Women Children's Hospital	Research Ethics Criteria	Local (Ethical)	-
99	China	Shengjing Hospital	In Chinese script	Local (Ethical)	-
100	China	Wenzhou Medical University Affiliate 2nd Hospital and Yuying Children Hospital	Research Ethics Criteria	Local (Ethical)	-
101	China	Wuhan Children's Hospital	In Chinese script	Local (Ethical)	-
102	Colombia	Bogota Hospital Militar Central	Director General	Local (Hospital)	2018046
103	Colombia	Bogota Hospital San Jose Centro Society for Surgery	President of Society	Local (Departmental)	-
104	Colombia	Fundacion Valle de Lili Cali Colombia	Research and Biomedical Ethics Committee	Local (Ethical)	234-2018
105	Colombia	Materno Infantil Saludcoop, Villavicencio	Medical Director	Local (Hospital)	-
106	Dominican Republic	Hospital Infantil Dr. Robert Reid Cabral, Santa Domingo	Investigational Committee	Local (Hospital)	
107	Ecuador	Hospital Especialidades Carlos Andrade Martin, Quito	Technical Director	Local (Hospital)	IESS-HJCA-DT-2018-5098-M
108	Ecuador	Hospital de Especialidades José Carrasco Arteaga, Cuenca	Hospital Director	Local (Hospital)	-
109	Ecuador	Hospital de Los Valles	Medical Director	Local (Hospital)	-
110	Ecuador	Hospital General del Norte de Guayaquil	Deputy Director of Teaching	Local (Hospital)	IESS-HG-NGC-DO-2018-0008-O
111	Ecuador	Omni Hospital, Gayaquil	Director of Teaching	Local (Hospital)	-
112	Egypt	Al Zahra Hospital	In Arabic script	Local (Ethical)	-
113	Egypt	Alexandria University Children's Hospital	Ethics Committee	Local (ethical)	0304041
114	Egypt	Assiut University Hospital	In Arabic script	Local (Ethical)	-
115	Egypt	Mansoura University Children's Hospital	Head of Department of Pediatric Surgery Council	Local (Departmental)	0032610/2020/81A
116	Egypt	Nasser Institution for Research and Treatment, Cairo	Hospital Director Letter	Local (Ethical)	-
117	Egypt	Tanta University Hospital	In Arabic script	Local (Ethical)	-
118	Gabon	Bongolo Hospital, Lebamba	Institutional Review Board at Bongolo Hospital	Local (Ethical)	-
119	Ghana	Korle-Bu Teaching Hospital	Scientific and Technical Committee & KBTH Institutional Review Board	Local (Ethical)	KBTH/MIS/G3/19
120	Ghana	Tamale Teaching Hospital	Head of Research and Development	Local (Ethical)	TTH/R&D/SR/18/8
121	India	Bazaricherra, Karimganj District	Chairman Research Committee	Local (Ethical)	-
122	India	Christian Medical college Ludhiana, Punjab	Institutional Research Committee	Local (Ethical)	-
123	India	Kasturba Medical College (Kasturba Hospital) Manipal	Institutional Ethics Committee	Local (Ethical)	ECR/146/Inst/KA/2013/RR-16
124	India	Makunda Christian Leprosy and General Hospital, Bazaricherra, Karimganj District	Chairman of Research Committee	Local (Ethical)	-

For peer review only

125	Indonesia	Dr Soetomo Hospital, Surabaya	President of Indonesian Association of Pediatric Surgeons and Head of Institutional Review Board	Local (Association and Ethical)	83/PERBANI/XI/2018 IRB/22/04/ETIK/2019
126	Indonesia	Rumah Sakit Anak dan Bunda Harapan Kita Jakarta	Indonesian Association of Pediatric Surgeons president approval	Local and Regional	-
127	Iran	Mofid Children's Hospital (MCH)	Research Council of Research Institute for Children Health	Local (Ethical)	0477, 162
128	Iraq	Al-Mustansiriyah University Central	Ministry of Higher Education and Scientific Research	Local (Ethical)	239
129	Iraq	Child's Central Teaching Hospital, Baghdad/Mustansiriyah Medical College	Ethical Committee	Local (Ethical)	-
130	Iraq	University of Kufa	Medical Ethics Committee for University	Local (Ethical)	MEC-035
131	Jordan	Al-Basheer Hospital	Al-Basheer Hospital and Chairman of Pediatric Surgery	Local (Departmental, Hospital)	15999
132	Jordan	Jordon University Hospital of Medicine	Institutional Review Board	Local (Ethical)	10/2018/2448
133	Jordan	JUST King Abdallah University Hospital	Institutional Review Board	Local (Ethical)	4/118/2018
134	Kenya	Aga Khan University Hospital	Research Ethics Committee, Aga Khan University	Local (Ethical)	2018/REC-138 (v1)
135	Kenya	Joromongi Oginga Odinga Teaching & Referral Hospital, Kisumu	JOOTRH Institutional Ethics and Research Committee	Local (Ethical)	ERC.IB/VOL.1/475
136	Kenya	University of Nairobi and Kenyatta National Hospital	Kenyatta National Hospital – University of Nairobi Ethics and Research Committee	Local (Ethical)	KNH-ERC/A/6
137	Libya	Al Fardous Clinic	Ethical Approval from Head of Medical Services	Local (Ethical)	2/018 P.
138	Libya	Alaml Specialised Hospital, Misurata	Ethical Approval from Head Director of Alaml Specialized Hospital	Local (Ethical)	-
139	Libya	Asalam, Misurata	In local script	Local (Ethical)	-
140	Libya	Assafwa international hospital, Misurata	Ethical Approval from Head of Medical Service	Local (Ethical)	-
141	Libya	Dar Alhekma Hospital	Head Director of Dar Alhekma Specialized Hospital	Local (Ethical)	-
142	Libya	Ghout El-Shaal Specialized Hospital	In local script	Local (Ethical)	-
143	Libya	Misarata Cancer Centre	Director of Medical Affairs, Chief of Medical Ethics	Local (Ethical)	708/42.
144	Libya	Misurata Medical Centre	Medical Director	Local (Ethical)	-
145	Libya	Tripoli Children's Hospital	Ethical Committee	Local (Ethical)	NCDC:E17:2018.
146	Libya	Tripoli Medical Centre	Hospital Director	Local (Hospital)	-
147	Macedonia	University Clinic for Paediatric Surgery	Ethical Committee	Local (Ethical)	-
148	Malaysia	Hospital Canselor Tuanku Muhriz, UKM Medical Centre	Research Ethics Committee	Local (Ethical)	UKM PPI/111/8/JEP-2018-464
149	Malaysia	Hospital Kuala Lumpar	Medical Research and Ethics Committee of Ministry of Health Malaysia	Regional (Ethical)	KKM/NIHSEC/ P18-1816 (14)
150	Malaysia	Hospital Pulau Pinang	Medical Research and Ethics Committee of Ministry of Health Malaysia	Regional (Ethical)	KKM/NIHSEC/ P18-1816 (14)
151	Malaysia	Hospital Raja Perempuan Zainab II, Kota Bharu, Kelantan	Medical Research and Ethics Committee of Ministry of Health Malaysia	Regional (Ethical)	KKM/NIHSEC/ P18-1816 (14)
152	Malaysia	Hospital Sultanah Aminah, Johor Bharu, Johor	Medical Research and Ethics Committee of Ministry of Health Malaysia	Regional (Ethical)	KKM/NIHSEC/ P18-1816 (14)
153	Malaysia	Hospital Sultanah Nur Zahirah, Kuala Terengganu, Terengganu	Medical Research and Ethics Committee of Ministry of Health Malaysia	Regional (Ethical)	KKM/NIHSEC/ P18-1816 (14)
154	Malaysia	Hospital Tengku Ampuan Afzan, Kuantan	Medical Research and Ethics Committee of Ministry of Health Malaysia	Regional (Ethical)	KKM/NIHSEC/ P18-1816 (14)
155	Malaysia	Hospital Wanita dan Kanak-Kanak, Likas, Sabah	Medical Research and Ethics Committee of Ministry of Health Malaysia	Regional (Ethical)	KKM/NIHSEC/ P18-1816 (14)

For peer review only - http://bmjopen.bmj.com/site/about/guidelines.xhtml

156	Malaysia	Penang General Hospital	Ethics & Medical research Committee	Local (Ethical)	KKM/NIHSEC/P18-1816(11)
157	Mexico	Hospital General Tijuana, Baja California	Research and Ethics Committee	Local (Ethical)	-
158	Mexico	Hospital Civil de Guadalajara	Research Committee and Ethics Committee	Local (Ethical)	0247/18 HCJIM/2018 and HCG/CEI-0913/18
159	Mexico	Hospital de Especialidades del Nino y Mujer de Queretaro	Research Committee	Local (Hospital)	135/10-10-2018/CIR.PED / HENM
160	Mexico	Hospital del Niño Poblano	Research Committee	Local (Hospital)	HNP 2018-16
161	Mexico	Hospital Infantil de Mexico Federico Gomez	Committee of Research, Ethics and Biosecurity	Local (Ethical)	HIM 2018-043
162	Mexico	Hospital Regional de Alta Especialidad del Niño “Dr. Rodolfo Nieto Padrón”	Research and Ethics Committee	Local (Ethical)	DI/051/18
163	Mexico	Moctezuma Children’s Hospital	Research and Ethics Committee	Local (Ethical)	09-CEI-006-20180328
164	Mexico	Pediatric Hospital, Western Medical Center, Mexican Institute of Social Security	SIRELCIS Administration	Local (Hospital)	F-CNNIC-2018-209
165	Morocco	Centre Hospitalier Universaitaire IBN Sina de Rabat, Rabat	Chief of Paediatric Emergency Services	Local (Departmental)	-
166	Nigeria	Amino Kano Teaching Hospital	Research Ethics Committee	Local (Ethical)	LAKTH/MAC/SUB/12A/ P-3/VI/2402
167	Nigeria	Bowen University Teaching Hospital	Ethics Committee	Local (Ethical)	NHREC/12/04/2012
168	Nigeria	Federal Medical Centre Abeokuta	Health Research Ethics Committee	Local (Ethical)	FMCA/470/19
169	Nigeria	Federal Medical Centre Umuahia	Health Research Ethics Committee	Local (Ethical)	FMC/QEH/G.596/Vol.10/ 371
170	Nigeria	Federal Medical Centre Yola	Research and Ethics Committee	Local (Ethical)	FMCY/SUB/S.128/042
171	Nigeria	Federal Teaching Hospital Gombe	Health Research Ethics Committee	Local (Ethical)	NHREC/25/10/2013
172	Nigeria	Lagos State University Teaching Hospital	Health Research Ethics Committee of LASUTH (LREC)	Local (Ethical)	NHREC04/04/2008
173	Nigeria	Lagos University Teaching Hospital	Lagos University Teaching Hospital Health Research Ethics Committee (LUTHHREC)	Local (Ethical)	NHREC 19/12/2008a
174	Nigeria	National Hospital Abuja	Institute Review Board Committee	Local (Ethical)	NHA/EC/054/2018
175	Nigeria	Nnamdi Azikiwe University Teaching hospital	Ethics Committee	Local (Ethical)	NAUTH/CS/668/VOL.2 /024 and NAUTH/CS/66/VOL.11/ 171/2018/107
176	Nigeria	Olabisi Onabanjo University Teaching Hospital	Health Research Ethics Committee	Local (Ethical)	NHREC/28/11/2017
177	Nigeria	University College Hospital, Ibadan	Ethics Committee and Head of Department of Surgery	Local (Ethical)	UI/EC/18/0432
178	Nigeria	University of Abuja Teaching Hospital	Health Research Ethics Committee	Local (Ethical)	UATH/HREC/PR/2019/001
179	Nigeria	University of Nigeria Teaching Hospital	Health Research Ethics Committee	Local (Ethical)	UNTH/CSA/329/OL5
180	Pakistan	Aga Khan University	Ethics Review Committee	Local (Ethical)	2019-0454-2658
181	Pakistan	Children's Hospital, PIMS, and Shaheed Zulfiqar Ali Bhutto Medical University, Islamabad	Ethical Review Board	Local (Ethical)	1-1 /2015 /ERB /SZABMU /274
182	Pakistan	Karachi Indus hospital	Interactive Research and Development Institute Review Board	Local (Ethical)	IRD_IRB_2018_07_012
183	Pakistan	Lahore King Edward Medical University	Institute Review Board	Local (Ethical)	109/RC/KEMU
184	Pakistan	Liaquat National Hospital	Ethics Review Committee	Local (Ethical)	0434-2018 LNH-ERC
185	Pakistan	The Children’s Hospital and the Institute of Child Health	Institute Review Board	Local (Ethical)	45868
186	Palestine, Gaza	Al-Aqsa Hospital, Gaza Strip	Helsinki Committee for Ethical Approval	National (ethical)	PHRC/HC/41//18
187	Palestine, Gaza	Al-Shifa Hospital, Gaza Strip	Helsinki Committee for Ethical Approval	National (ethical)	PHRC/HC/41//18
188	Palestine, Gaza	European Gaza Hospital, Gaza Strip	Helsinki Committee for Ethical Approval	National (ethical)	PHRC/HC/41//18
189	Palestine, West Bank	Ahli Hospital, Hebron	In Arabic script	National/Local (ethical)	-

For peer review only - http://bmjopen.bmj.com/site/about/guidelines.xhtml

190	Palestine, West Bank	Jenin Government Hospital, Jenin	In Arabic script	National/Local (ethical)	-
191	Palestine, West Bank	Mount of Olives/Makassed, Jerusalem	In Arabic script	National/Local (ethical)	-
192	Palestine, West Bank	Palestinian Medical Complex (PMC), Ramallah	In Arabic script	National/Local (ethical)	-
193	Palestine, West Bank	Rafidia Surgery Hospital, Nablus	In Arabic script	National/Local (ethical)	-
194	Palestine, West Bank	Red Crescent Society, Hebron	In Arabic script	National/Local (ethical)	-
195	Papua New Guinea	Mount Hagen General Hospital	Director Clinical Excellence	Local (Hospital)	-
196	Papua New Guinea	Port Moresby General Hospital	Director Medical Services and Ethical Committee	Local (Ethical)	-
197	Peru	Cayetano Heredia Hospital	Institutional Committee of Ethics and Investigation	Local (Ethical)	-
198	Peru	Guillermo Almenara National Hospital, Lima	Ethical Committee	Local (Ethical)	174-2018
199	Peru	Hospital Dos de Mayo	Ethics committee in biomedical research	Local (Ethical)	085-2018-CEIB-HNDM
200	Peru	Hospital Nacional Alberto Sabogal Sologuren	Institutional committee on ethics in research	Local (Ethical)	549-OAIyD-HNASS-ESSALUD-2018<br>79-UCP-DC-GQ-HNASS-ESSALUD-2018
201	Peru	Hospital Nacional Docente Madre Niño San Bartolomé	Committee of Institutional Ethics and Research	Local (Ethical)	12200-18
202	Peru	Hospital Nacional Edgardo Rebagliati Martins	Ethics Committee	Local (Ethical)	943-OCIYD-GHNERM-GRPR-ESSALUD-2018
203	Peru	Hospital Regional Del Cusco	Research committee and training unit	Local (Hospital)	8868-18.
204	Peru	Instituto Nacional de Salud Del Niño	Institutional Committee of Ethics and Investigation	Local (Ethical)	PI-64/18
205	Peru	Instituto Nacional de Salud de Nino de San Borja	Institutional Committee of Ethics and Investigation	Local (Ethical)	PI-2018-246
206	Peru	Instituto Nacional Materno Perinatal	Health Minister	Local (Hospital)	699-2018-DEN/INMP
207	Peru	Pediatric Emergencies Hospital	Head of the Teaching and Research Support Office	Local (Hospital)	065-2018-OADI-HEP/MINSA
208	Philippines	Philippines Children's Medical Center	Institutional Review Board – Ethics Committee	Local (Ethical)	PCMC IRB-EC 2018-040
209	Romania	Children Clinical Hospital of Brasov, Brasov/University of Brasov	Ethical Committee	Local (Ethical)	-
210	Romania	Marie Curie Hospital in Bucharest	Ethical Committee	Local (Ethical)	-
211	Serbia	Clinical Centre for Paediatric Surgery and Orthopaedics, Clinical Center Niš	Ethics Committee of the Faculty of Medicine University of Niš President	Local (Ethical)	12-3182-2/11
212	Serbia	University Children's Hospital	Ethics Committee	Local (Ethical)	14/390
213	South Africa	Frere Hospital	Clinical Governance Committee and Frere and Cecilia Makiwane Hospitals Research Ethics Committee	Local (Ethical)	FCMHREC/013/2019
214	South Africa	Greys Hospital, Pietermaritzburg	Subcommittee of Biomedical Research Ethics Committee	Local (Ethical)	BCA221/13
215	South Africa	Grootte Schurr Hospital	Human Research Ethics Committee and Medical Services Manager	Local (Ethical)	572/2018 and RCC158
216	South Africa	Red Cross War Memorial	Human Research Ethics Committee and Medical Services Manager	Local (Ethical)	572/2018 and RCC158
217	South Africa	Tygerberg Children's Hospital	Human Research Ethics Committee and Medical Services Manager	Local (Ethical)	N18/09/101
218	Sri Lanka	Lady Ridgeway Hospital for Children	Ethical Committee	Local (Ethical)	-
219	Sudan	Khartoum University Hospital	Head of Department of Paediatric Surgery	Local (Departmental)	-
220	Syria	Aleppo University Hospital	Dean of Faculty of Medicine	Local (Dean)	-
221	Syria	AlShahbaa Hospital, Aleppo city	Head of Alshahbaa Private Hospital	Local (Hospital)	-

For peer review only - http://bmjopen.bmj.com/site/about/guidelines.xhtml

222	Syria	Tishreen University Hospital	In Arabic	Local (Ethical)	-
223	Thailand	Bangkok Ramathibodi Hospital	Institutional Review Board	Local (Ethical)	07-61-17
224	Thailand	Chiangmai Jiraporn Khorana	Ethics Committee	Local (Ethical)	SUR-2561-05619
225	Thailand	Hatyai Hospital	Research Ethics Committee	Local (Ethical)	80/2561
226	Thailand	Khon Kaen University	Ethics Committee for Human Research	Local (Ethical)	HE611526
227	Thailand	National Institute of Child Health (QSNICH)	Research Ethics Review Committee	Local (Ethical)	61-065
228	Thailand	Siriraj Hospital	Institutional Review Board	Local (Ethical)	687/2561(EC2)
229	Thailand	Sisaket Hospital	Research Ethics Committee	Local (Ethical)	071/2561.
230	Tunisia	CHU Hedi Chaker Hospital	President of the Ethical Committee	Local (Ethical)	-
231	Tunisia	Fattouma Bourguiba Hospital	President of the Ethical Committee	Local (Ethical)	-
232	Turkey	Adana State Training Hospital	Head Physician	Local (Hospital)	60247264-799-41
233	Turkey	Amerikan Hospital, Istanbul	Ethics Committee Chairman	Local (Ethical)	2018.235.irbi.028.
234	Turkey	Bezmialem University School of Medicine	Hospital President	Local (Hospital)	54022451-050.05.04
235	Turkey	Biruni University Hospital	University Ethics Committee	Local (Ethical)	-
236	Turkey	Gazi University School of Medicine	University Dean	Local (Dean)	24074710-604.01.01-47
237	Turkey	Karadeniz technical University	Head Physician	Local (Hospital)	E.10031
238	Turkey	Koç University	Ethics Committee Chairman	Local (Ethical)	2018.235.irbi.028
239	Turkey	Muğla Education and Research Hospital	Chief of Staff	Local (Hospital)	2018.235.IRB1.028
240	Uzbekistan	Republican Perinatal Center	In local script	Local (Ethical)	000174
241	Uzbekistan	Republican Specialized Scientific Practical Medical Center of Pediatrics, Tashkent	In local script	Local (Ethical)	-
242	Zambia	University Teaching Hospital, Lusaka	ERES Converge I Institutional Review Board	Local (Ethical)	2018-Sept-001
**TOTAL MICs:** 217					
**High-Income Countries (HICs)**					
243	Australia	Children's Hospital at Westmead, Sydney	Research Governance Officer and Human Research Ethics Committee	Local (Ethical)	LNR/18/SCHN/347
244	Australia	Gold Coast University Hospital	Human Research Ethics Committee	Local (Ethical)	HREC/18/QGC/47042
245	Australia	Monash Children's Hospital, Victoria	Human Research Ethics Committees	Local (Ethical)	HREC/43418/MonH-2018-67965(v1)
246	Australia	The Royal Children's Hospital, Melbourne	Murdoch Children's Research Institute and Research Ethics and Governance Officer	Local (Ethical)	QA/50138/RCHM-2018
247	Austria	Medical University of Graz	Ethics Committee	Local (Ethical)	31-157 ex. 18/19
248	Austria	Medical University of Vienna, Spitalgasse	Ethics Committee	Local (Ethical)	2006/2018
249	Belgium	Queen Fabiola University Children's Hospital	Ethics Committee	Local (Ethical)	B406201837832
250	Brunei Darussalam	RIPAS Hospital, Bandar Seri Begawan	Medical and Health Research and Ethics Committee	Local (Ethical)	MHREC/MOH/2018/7
251	Canada	Ste-Justine Children's Hospital	Scientific Research Committee and Research Ethics Board for Ethical Review	Local (Ethical)	2019-2158
252	Chile	San Juan de Dios Hospital	Ethics Committee for Scientific Investigation	Local (Ethical)	47/2018
253	Czech Republic	Fakultni Nemocnice BRNO	Ethics Committee	Local (Ethical)	13-130219/EK
254	Czech Republic	University Hospital Hradec Kralove	Ethics Committee	Local (Ethical)	201812 s17p
255	Czech Republic	University Hospital Motol	Ethics Committee	Local (Ethical)	-
256	England	Alder Hey Children's Hospital	Governance and Quality Assurance Team	Local (Hospital)	5714
257	England	Birmingham Children's Hospital	Clinical Audit Registration and Management System	Local (Hospital)	CARMS-30164
258	England	Bristol Royal Hospital for Children	Audit Governor	Local (Hospital)	-
259	England	Evelina Children's Hospital, Guy's & St.Thomas' NHS Trust	Audit Team	Local (Hospital)	8956

For peer review only - http://bmjopen.bmj.com/site/about/guidelines.xhtml

260	England	John Radcliffe Hospital, Oxford	Clinical Effectiveness Committee	Local (Hospital)	5150
261	England	King's College Hospital	Governance Manager	Local (Hospital)	CH011
262	England	Leeds General Infirmary	Clinical Director and Deputy Caldicott Guardian	Local (Hospital)	Globalpaedsurg
263	England	Leicester and Kettering Hospitals	Clinical Audit Facilitator	Local (Hospital)	9482e
264	England	Nottingham Hospital	Audit Department	Local (Hospital)	18-350C
265	England	Southampton General Hospital	Audit Department	Local (Hospital)	SEV/0049
266	England	St Georges Hospital	Audit Department	Local (Hospital)	AUD1000163
267	France	CHU Amiens	Ethical Review Board	National (Ethical)	18.105
268	France	CHU Amiens-Picardie, Amiens	Ethical Review Board	National (Ethical)	18.105
269	France	CHU Angers	Ethical Review Board	National (Ethical)	18.105
270	France	CHU APHM-Marseille	Ethical Review Board	National (Ethical)	18.105
271	France	CHU APHP-Bicêtre	Ethical Review Board	National (Ethical)	18.105
272	France	CHU APHP-Necker	Ethical Review Board	National (Ethical)	18.105
273	France	CHU APHP-Robert Debré	Ethical Review Board	National (Ethical)	18.105
274	France	CHU Bordeaux	Ethical Review Board	National (Ethical)	18.105
275	France	CHU Limoges	Ethical Review Board	National (Ethical)	18.105
276	France	CHU Poitiers	Ethical Review Board	National (Ethical)	18.105
277	France	CHU Rennes	Ethical Review Board	National (Ethical)	18.105
278	France	CHU Rouen	Ethical Review Board	National (Ethical)	18.105
279	France	CHU StEtienne	Ethical Review Board	National (Ethical)	18.105
280	France	CHU Toulouse	Ethical Review Board	National (Ethical)	18.105
281	Germany	Frankfurt University Hospital	Ethics Committee	Local (Ethical)	346/18
282	Germany	Hospital St. Barbara Elisabeth Halle	Ethics Committee	Local (Ethical)	-
283	Germany	Krankenhaus Barmherzige Brüder Regensburg	Ethics Committee	Local (Ethical)	19-1263-101
284	Germany	Kinderchirurgie Vivantes Neukölln, Berlin	Coordinator of Clinical Research and Academic Teaching	Local (Hospital)	-
285	Germany	Medical Faculty Otto-von-Guericke University Magdeburg	Executive Director of Ethics Committee	Local (Ethical)	151/18
286	Germany	Universitat zu Lubeck	Ethics Committee	Local (Ethical)	18-249
287	Italy	San Matteo Hospital	Secretary to the Head of Science Technology	Local (Hospital)	20180097159
288	Hungary	Albert Szent-Györgyi Clinical Centre, Szeged	Human Investigation Review Board	Local (Ethical)	4413
289	Lithuania	Children's Hospital, Affiliate of Vilnius University Hospital SK	Chairman	Local (Hospital)	18VSR-1735<br>158200-18/9-1061-562
290	Lithuania	Lithuanian University of Health Sciences	Chairman of Kaunas RBTEK	Local (Hospital)	-
291	New Zealand	Christchurch Hospital	Quality Co-ordinator Childs Health and Audit Group	Local (Hospital)	BE-2-82
292	New Zealand	Starship Children's Hospital, NZ	Southern health and Disability Ethics Committee	Local (Ethical)	18/STH/143
293	New Zealand	Waikato Hospital	Director of Quality and Patient Safety	Local (Hospital)	RD018104
294	New Zealand	Wellington Hospital	Operations Manager Child Health Service	Local (Hospital)	-
295	Poland	Department of Children's Developmental Defects Surgery and Traumatology	Bioethical Commission	Local (Ethical)	KNW/0022/KB/204/I/18
296	Poland	Medical University of Gdańsk	Bioethical Committee for Research	Local (Ethical)	NKBBN/456/2018
297	Poland	Medical University of Silesia, Katowice	Bioethical Commission	Local (Ethical)	KNW/0022/KB/172/18
298	Poland	Warsaw Children's Memorial Health Institute	Bioethical Commission and Ethical Committee Assembly	Local (Ethical)	20/KBE/2018
299	Poland	Wladyslaw Buskowski Children's Hospital	Bioethical Commission	Local (Ethical)	44/2018
300	Poland	Wroclaw University	Bioethical Commission	Local (Ethical)	133/XV R/2017

For peer review only - http://bmjopen.bmj.com/site/about/guidelines.xhtml

301	Qatar	Sidra Medecine, Doha	Institutional Review Board	Local (Ethical)	1808029580
302	Saudi Arabia	King Fahd Armed Forces Hospital	Director of the Research Center, Research and Ethics Committee	Local (Ethical)	REC 261
303	Saudi Arabia	King Saud University College of Medicine	Institutional Review Board	Local (Ethical)	E-18-3427
304	Scotland	Glasgow Children's Hospital, NHS Greater Glasgow and Clyde	Caldicott Guardian	Local (Hospital)	-
305	Scotland	Royal Hospital for Sick Children	Associate Medical Director	Local (Hospital)	-
306	Singapore	KK Women's and Children's Hospital, Little India	Head of Department	Local (Departmental)	-
307	South Korea	Cheonnam National University Hospital, Dong-gu, Gwangju	Chonnam National University Hospital Biomedical Research Ethics Review Committee Chairperson	Local (Ethical)	-
308	South Korea	Samsung Medical Centre	Institutional Review Board	Local (Ethical)	2018-09-110
309	South Korea	Seoul National University Children's Hospital	Institutional Review Board	Local (Ethical)	H-1810-050-977
310	South Korea	Seoul St. Marys Hospital	Institutional Review Board	Local (Ethical)	KC18OCGI0669
311	Spain	Barcelona Sant Joan de Déu Hospital	Secretary of CEIM Sant Joan de Déu	Local (Hospital)	-
312	Spain	Complejo Hospitalario Universitario de a Coruña (CHUAC)	Head of Paediatric Surgery Unit	Local (Departmental)	-
313	Spain	Hospital Clinico Universitario de Valladolid	Director Manager and CEIM of Valladoid Health Area	Local (Hospital)	-
314	Spain	Maternal Hospital Infantil de Badajoz	Secretary of the Ethical Investigation Committee of Badajoz Clinic	Local (Ethical)	-
315	Spain	Zaragoza Hospital Universitario Miguel	Favourable Report Project of Biomedical Research a Secretary of the CEIC Aragón (CEICA)	Local (Hospital)	-
316	Sweden	Lund Skåne University Hospital Pediatric Care Hospital	Consultation group for quality registers	Local (Hospital)	-
317	Sweden	Queen Silvia Children's Hospital	Area Manager of QSCH and Head of operations or unit manager	Local (Hospital)	-
318	Sweden	Sachs Children's Hospital	Patient Area Director	Local (Hospital)	-
319	Sweden	Uppsala University Children's Hospital	Section Chief, Paediatric Surgery Clinician	Local (Departmental)	-
320	Switzerland	Inselspital/University Hospital, Bern	Kantonale Ethics Committee	Local (Ethical)	-
321	UAE	Danat Al Emarat Hospital	Head of Surgery	Local (Departmental)	-
322	Uruguay	University De La Republica Facultad de Medicina	Service Chief for the Neonatal Department	Local (Departmental)	-
323	Uruguay	Centro Hospitalario Pereira Rossell	Audit Team	Local (Hospital)	-
324	USA	Ann and Robert H. Lurie Children's Hospital of Chicago	Institutional Review Board	Local (Ethical)	IRB 2019-2334
325	USA	Beaumont's Children's Hospital in Royal Oak	Institutional Review Board	Local (Ethical)	IRB 2018-402
326	USA	Children's Hospital of Wisconsin	Institutional Review Board	Local (Ethical)	1352795-2
327	USA	Dartmouth-Hitchcock Medical Center	Committee for the Protection of Human Subjects	Local (Ethical)	STUDY00031666
328	USA	Le Bonheur Children's Hospital Memphis	UTHSC Institutional Review Board	Local (Ethical)	18-06176-XP
329	USA	Nationwide Children's Hospital	Institutional Review Board Office	Local (Ethical)	IRB18-01005
330	USA	New York Presbyterian Morgan Stanley Children's Hospital	Institutional Review Board	Local (Ethical)	IRB-AAAS0645
331	USA	Oregon Health and Science University	Institutional Review Board	Local (Ethical)	STUDY00018665
332	USA	Phoenix Children's Hospital	Institutional Review Board	Local (Ethical)	PCH IRB #18-100
333	USA	University of Miami	Institutional Review Board	Local (Ethical)	20181054
334	USA	University of Michigan	Co-chairs of Institutional Review Board MED	Local (Ethical)	HUM 00151299
335	USA	University of Texas Medical Branch	Institutional Review Board Vice Chairman	Local (Ethical)	IRB18-0318

For peer review only

336	USA	UT Southwestern Medical Center Children's Hospital	Institutional Review Board	Local (Ethical)	STU 072018-058
337	USA	Yale New Haven Hospital	Institutional Review Board	Local (Ethical)	2000024339
**TOTAL HICs:** 95					
**OVERALL TOTAL: 337**					

For peer review only

For peer review only - http://bmjopen.bmj.com/site/about/guidelines.xhtml

Management and Outcomes of Gastrointestinal Congenital Anomalies in Low-, Middle- and High-Income Countries: Protocol for a Multi-Centre, International, Prospective Cohort Study

STROBE checklist detailing where each item is addressed in the protocol

	Item No	Recommendation	Section where this is covered in the manuscript
Title and abstract	1	(a) Indicate the study's design with a commonly used term in the title or the abstract	Title, abstract
		(b) Provide in the abstract an informative and balanced summary of what was done and what was found	Abstract
Introduction			
Background/rationale	2	Explain the scientific background and rationale for the investigation being reported	Introduction
Objectives	3	State specific objectives, including any prespecified hypotheses	Objective
Methods			
Study design	4	Present key elements of study design early in the paper	Abstract, Methods - Study Design
Setting	5	Describe the setting, locations, and relevant dates, including periods of recruitment, exposure, follow-up, and data collection	Methods
Participants	6	(a) Give the eligibility criteria, and the sources and methods of selection of participants. Describe methods of follow-up	Methods – inclusion/ exclusion criteria, outcome measures
		(b) For matched studies, give matching criteria and number of exposed and unexposed	Not Applicable
Variables	7	Clearly define all outcomes, exposures, predictors, potential confounders, and effect modifiers. Give diagnostic criteria, if applicable	Methods – Outcomes, Data Collection, Data Analysis, Appendix
Data sources/ measurement	8*	For each variable of interest, give sources of data and details of methods of assessment (measurement). Describe comparability of assessment methods if there is more than one group	Table 1, Methods – sample selection, Appendix 1 and 2
Bias	9	Describe any efforts to address potential sources of bias	Data quality, data validation
Study size	10	Explain how the study size was arrived at	Sample size calculation
Quantitative variables	11	Explain how quantitative variables were handled in the analyses. If applicable, describe which groupings were chosen and why	Not applicable
Statistical methods	12	(a) Describe all statistical methods, including those used to control for confounding	Data Analysis
		(b) Describe any methods used to examine subgroups and interactions	Data Analysis
		(c) Explain how missing data were addressed	Data Analysis
		(d) If applicable, explain how loss to follow-up was addressed	Data Analysis
		(e) Describe any sensitivity analyses	Data Analysis
Results			
Participants	13*	(a) Report numbers of individuals at each stage of study—eg numbers potentially eligible, examined for eligibility, confirmed eligible, included in the study, completing follow-up, and analysed	Estimated Study Population
		(b) Give reasons for non-participation at each stage	Not applicable
		(c) Consider use of a flow diagram	Not applicable
Descriptive data	14*	(a) Give characteristics of study participants (eg demographic, clinical, social) and information on exposures and potential confounders	Not applicable
		(b) Indicate number of participants with missing data for each variable of interest	Not applicable
		(c) Summarise follow-up time (eg, average and total amount)	Not applicable
Outcome data	15*	Report numbers of outcome events or summary measures over time	Not applicable
Main results	16	(a) Give unadjusted estimates and, if applicable, confounder-adjusted estimates and their precision (eg, 95% confidence interval). Make clear which confounders were adjusted for and why they were included	Not applicable
		(b) Report category boundaries when continuous variables were categorized	Not applicable
		(c) If relevant, consider translating estimates of relative risk into absolute risk for a meaningful time period	Not applicable
Other analyses	17	Report other analyses done—eg analyses of subgroups and interactions, and sensitivity analyses	Data Analysis
Discussion			
Key results	18	Summarise key results with reference to study objectives	Not applicable
Limitations	19	Discuss limitations of the study, taking into account sources of potential bias or imprecision. Discuss both direction and magnitude of any potential bias	Strengths and Limitations
Interpretation	20	Give a cautious overall interpretation of results considering objectives, limitations, multiplicity of analyses, results from similar studies, and other relevant evidence	Not applicable

For peer review only - http://bmjopen.bmj.com/site/about/guidelines.xhtml

Generalisability	21	Discuss the generalisability (external validity) of the study results	Not applicable
Other information			
Funding	22	Give the source of funding and the role of the funders for the present study and, if applicable, for the original study on which the present article is based	Additional Information

For peer review only

Management and Outcomes of Gastrointestinal Congenital Anomalies in Low-, Middle- and High-Income Countries: Protocol for a Multi-Centre, International, Prospective Cohort Study

Journal:	*BMJ Open*
Manuscript ID	bmjopen-2019-030452.R2
Article Type:	Protocol
Date Submitted by the Author:	10-Jul-2019
Complete List of Authors:	Wright, Naomi; King's College London, King's Centre for Global Health and Health Partnerships
**Primary Subject Heading**:	Global health
Secondary Subject Heading:	Paediatrics, Surgery
Keywords:	PAEDIATRIC SURGERY, Paediatric anaesthesia < ANAESTHETICS, Paediatric intensive & critical care < INTENSIVE & CRITICAL CARE, NEONATOLOGY, PAEDIATRICS, Neonatal intensive & critical care < INTENSIVE & CRITICAL CARE

Management and Outcomes of Gastrointestinal Congenital Anomalies in Low-, Middle- and High-Income Countries: Protocol for a Multi-Centre, International, Prospective Cohort Study

Global PaedSurg Research Collaboration

Correspondence to: Naomi J. Wright, King's Centre for Global Health and Health Partnerships, School of Population Health and Environmental Sciences, King's College London, Denmark Hill, SE5 9RJ, UK. Email: naomiwright@doctors.org.uk

For peer review only

ABSTRACT

Introduction

Congenital anomalies are the 5th leading cause of death in children under 5-years of age globally, contributing an estimated half a million deaths per year. Very limited literature exists from low- and middle-income countries (LMICs) where most of these deaths occur. The Global PaedSurg Research Collaboration aims to undertake the first multi-centre, international, prospective cohort study of a selection of common congenital anomalies comparing management and outcomes between low-, middle- and high-income countries (HICs) globally.

Methods and Analysis

The Global PaedSurg Research Collaboration consists of surgeons, paediatricians, anaesthetists and allied healthcare professionals involved in the surgical care of children globally. Collaborators will prospectively collect observational data on consecutive patients presenting for the first time, with one of seven common congenital anomalies (oesophageal atresia, congenital diaphragmatic hernia, intestinal atresia, gastroschisis, exomphalos, anorectal malformation and Hirschsprung's disease).

Patient recruitment will be for a minimum of one month from October 2018 to April 2019 with a 30-day post-primary intervention follow-up period. Anonymous data will be collected on patient demographics, clinical status, interventions and outcomes using REDCap. Collaborators will complete a survey regarding the resources and facilities for neonatal and paediatric surgery at their centre.

The primary outcome is all-cause in-hospital mortality. Secondary outcomes include the occurrence of post-operative complications. Chi-squared analysis will be used to compare mortality between LMICs and HICs. Multilevel, multivariate logistic regression analysis will be undertaken to identify patient level and hospital level factors affecting outcomes with adjustment for confounding factors.

Ethics and Dissemination

At the host centre this study is classified as an audit not requiring ethical approval. All participating collaborators have gained local approval in accordance with their institutional ethical regulations. Collaborators will be encouraged to present the results locally, nationally and internationally. The results will be submitted for open access publication in a peer reviewed journal.

Registration Details

This study has been registered with ClinicalTrials.Gov, identifier: NCT03666767. The registration is available to view via: https://goo.gl/ffXNMH

For peer review only - http://bmjopen.bmj.com/site/about/guidelines.xhtml

Strengths and Limitations of this Study

- This will be the first large-series, geographically comprehensive, multi-centre, international, prospective cohort study to define the management and outcomes of a selection of common congenital anomalies in low-, middle- and high-income countries across the globe.
- The collaborative approach for this study allows a large series of high-quality data to be collected in a timely manner without overburdening high-volume, low-resource centres.
- The seven study conditions constitute a selection of the commonest life-threatening congenital anomalies requiring emergency surgical care in the neonatal period **(Box 1)**.
- We recognise that some children may not reach a facility capable of providing acute paediatric surgical care and hence the results obtained may be an underestimation of true morbidity and mortality, especially in LMICs.
- The number of variables being collected per patient has been limited to those known to have the greatest impact on outcomes to optimise the feasibility of the study; follow-up is limited to 30-days post-primary intervention.

For peer review only

For peer review only - http://bmjopen.bmj.com/site/about/guidelines.xhtml

INTRODUCTION

In 2015, the Global Burden of Disease study concluded congenital anomalies (also known as congenital malformations, congenital abnormalities or birth defects) to be the 5th leading cause of death in children under 5-years of age globally.[1] This equates to approximately half a million deaths from congenital anomalies each year, 97% of which occur in low- and middle-income countries (LMICs). Indeed, this is likely to be an underestimation of the actual number of deaths due to under-diagnosis of neonates with congenital anomalies who die in the community and a lack of death certification in many LMICs.[2] Not only is the mortality rate higher in LMICs, the prevalence is also higher due to micronutrient deficiencies, infections and teratogens during pregnancy resulting in more cases and a lack of antenatal diagnosis prohibiting terminations.[3,4] There is limited research and a lack of congenital anomaly registries in LMICs and hence they have received very little global attention.[5]

The conditions forming the focus of this study **(Box 1)** constitute a selection of the most common life-threatening congenital anomalies during the neonatal period, which involve the gastrointestinal tract. They each have an incidence of 1/2000 – 1/5000, they collectively form up to 40% of emergency neonatal surgery and associated mortality can be in excess of 50% in many LMICs.[6-9] Disparities in outcomes globally can be stark; for example the mortality from gastroschisis is 75-100% in many LMICs compared to 4% or less in HICs.[10-12] Reasons for poor outcomes include a lack of antenatal diagnosis, delayed presentation, limited neonatal transport and in-hospital resources, a dearth of trained support personnel and a lack of intensive care and parenteral nutrition for neonates.[9,13,14] In Uganda, it was calculated that only 3.5% of the need for neonatal surgery was met by the healthcare system.[8]

Box 1. Congenital Anomalies in the Global PaedSurg Study

- Oesophageal atresia (OA) +/- tracheo-oesophageal fistula (TOF)
- Congenital diaphragmatic hernia (CDH)
- Intestinal atresia (IA)
- Gastroschisis
- Exomphalos
- Anorectal malformation (ARM)
- Hirschsprung’s disease

In 2010, the World Health Assembly passed a resolution recommending ‘prevention whenever possible, to implement screening programmes and to provide care and ongoing support to children with birth defects and their families’.[2] Prevention is paramount, however this is not yet possible for many congenital anomalies and hence a focus on improving postnatal care and outcomes is vital. The Sustainable Development Goal 3.2 aims to end preventable deaths of newborns and children under the age of 5-years by 2030.[6, 15,16] With a third of infant deaths being attributed to congenital anomalies, clearly this will not be achievable without an accelerated effort towards the provision of surgical care for children. It is estimated that two-thirds of deaths and disability from congenital anomalies can be avoided with the provision of neonatal and paediatric surgical care.[6] Indeed, studies have demonstrated such provision can be highly cost-effective in terms of disability adjusted life years saved.[5] Yet neonatal and paediatric surgical care remain a low priority on the global health agenda.[5]

A shift is needed to focus on the provision of surgical care for children within National Health Plans and International Organisations and to elevate congenital anomalies on the global health agenda. This large-scale, geographically comprehensive, multi-centre prospective cohort study aims to define the current management and outcomes of a selection of common congenital anomalies globally and identify factors affecting outcomes that can be modified to improve care. This is vital to aid advocacy and global health prioritisation and inform future interventional studies aimed at improving outcomes.

AIM

For peer review only - http://bmjopen.bmj.com/site/about/guidelines.xhtml

To undertake the first large-scale, geographically comprehensive multi-centre, prospective cohort study comparing the management and outcomes of a selection of common congenital anomalies in low-, middle-, and high-income countries across the globe.

OBJECTIVES

1. To compare the mortality and post-intervention complications of a selection of common congenital anomalies involving the gastrointestinal tract in LMICs and HICs globally.

2. To identify patient level and hospital level factors affecting outcomes that be modified to improve care.

3. To establish a research collaboration consisting of children's surgical care providers across the world to help enhance research capacity and to create a platform for ongoing collaborative research and intervention studies aimed at improving outcomes.

4. To raise awareness and provide advocacy for neonatal and paediatric surgical care within global health prioritisation, planning, policy and funding.

METHODS AND ANALYSIS

Study Design

This is an international, multi-centre, prospective observational cohort study. The Global PaedSurg Research Collaboration consisting of children's surgical care providers (collaborators) across the world was established from November 2017 to co-ordinate the study at an institutional level and facilitate data collection. Collaborators are free to choose one or more months between 1st October 2018 to the 30th April 2019 (inclusive) to recruit consecutive patients to the study, with a 30-day post-primary intervention follow up period. The primary intervention must occur within 30-days of presentation to be included in the study. Hence, the last date for primary data collection is 29th June 2019. Following this there will be a period of data collection for the data validation process continuing until the end of August 2019.

Collaborators

International collaborators will have a variety of roles and responsibilities within the study. Local collaborators will establish mini-teams locally, gain study approval, utilise the protocol criteria to appropriately identify patients for study inclusion, collect prospective data and upload it to REDCap. Each hospital will have a local study lead who will hold overall responsibility for ensuring the data is accurate, complete and without duplications. Country-lead collaborators will help to recruit other collaborators from within their country and provide advice and support regarding gaining local study approval and data collection. They may also help with translation of the study literature to the local language if required. Continent and regional leads will help to recruit country leads, provide them with advice regarding the study and also encourage and co-ordinate presentations of the protocol at national and international meetings. Lead investigators will contribute to the study design through the provision of feedback from the pilot studies undertaken in multiple languages. An organising committee will help to co-ordinate all study activities and a steering committee will provide guidance throughout.

There are a number of benefits for collaborators participating the study. Publishing journal(s) will be asked to make all collaborators PubMed citable co-authors. This is based on an equal partnership model described by the Lancet and is used by a number of national and international collaboratives.[17-21] All collaborators will be listed as an author on resulting presentations. Collaborators will have the opportunity to present the study locally, nationally and internationally, initially the study protocol and later the results. This often provides collaborators, especially those who are junior or from LMICs, the opportunity to apply for funding to attend, present and network at such meetings. Participation in the study provides an easy route and insight into clinical research, which can be further established

For peer review only - http://bmjopen.bmj.com/site/about/guidelines.xhtml

through participation in the 2-year Research Training Fellowship which is running alongside the main study free of charge for all interested collaborators.

Sample Selection

Collaborator and Hospital Inclusion Criteria:

All hospitals and healthcare professionals providing surgical care for neonates and children, presenting for the first time, with one or more of the study conditions can be included in the study. Collaborators should gain permission from the senior surgeon or physician who oversees the care of the children to be included in the study in order to participate. There can be up to three collaborators in a mini-team per month of data collection. One mini-team can collect data over one or more months or several mini-teams can collect data over a different month each. Each mini-team must contain at least one senior surgeon or physician to oversee the data collection process.

Patient Inclusion and Exclusion Criteria:

Any neonate, infant or child under the age of 16-years, presenting acutely for the first time, with one or more of the study conditions can be included in the study. Patients who have previously received surgery for their presenting condition or those representing with a complication of surgery are excluded. Patients presenting electively for surgery are excluded. Children who have received basic resuscitative care for their condition at a different healthcare facility and are then transferred to the study centre for their primary surgical intervention can be included. Children who only receive resuscitative treatment at the study centre and are then referred elsewhere for their primary surgical intervention cannot be included since the outcome of the surgical care will not be known and also to avoid the risk of duplicate patients in the study. Patients who receive conservative treatment as their primary intervention, palliative care, or no care must be included within the study to accurately reflect the management and outcomes of all presenting cases.

If a patient presents with more than one of the study conditions, the details of each condition that they present acutely with can be included, but not a previously managed condition. For example, a newborn presenting with oesophageal atresia and anorectal malformation would have both conditions included. A patient presenting for the first time with Hirschsprung's disease at several months of age who had a duodenal atresia repaired at birth would have the full details of the Hirschsprungs disease included, but the duodenal atresia would simply be noted as an associated anomaly.

Outcome Measures

The primary outcome is all-cause, in-hospital mortality.

For patient's hospitalised for over 30-days following primary intervention, a 30-day post-primary intervention mortality rate will be utilised. Those who do not receive a primary intervention, but remain alive and hospitalised at 30-days following primary admission, will have this time point used for recording their mortality status for the primary outcome. Primary outcome is defined in Table 1.

The secondary outcomes include complications occurring within 30-days of primary intervention:

- Surgical site-infection
- Wound dehiscence
- Need for re-intervention
- Condition specific complications
- Condition specific outcome variables
- Length of hospital stay or time from admission to death in patients who do not survive
- 30-day post primary intervention mortality.

Secondary outcomes will not be collected on patients who do not receive a primary intervention within 30-days of hospital admission, with the exception of length of hospital stay or time from admission to

For peer review only - http://bmjopen.bmj.com/site/about/guidelines.xhtml

death. 30-day follow-up will be undertaken within the capacity of the collaborating team; no additional funding will be provided.

Data Collection

Generic variables relating to the patient demographics, antenatal care, pre-hospital care, clinical condition, surgical intervention and outcomes will be collected for all patients in the study **(Table 1)**. Specific variables will be collected for each individual condition (**Supplementary File 1).**

Outcomes and variables have been chosen using published core outcome sets and commonly collected outcomes in systematic reviews and meta-analyses.[22-37] Collaborators will enter anonymous, de-identified data via the secure internet-based Research Electronic Data Capture (REDCap) system. This will be stored on King's College London REDCap server.

A short survey will be completed by the local study lead and one other collaborating consultant or registrar on the resources and facilities available for neonatal and paediatric surgical care at their centre **(Supplementary File 2)**.

Table 1. Generic Data Points

Generic questions	Answers
During which month did the patient present to your hospital?	Please select the month that the patient presented to your hospital for the first time with this congenital anomaly. For example, if a baby was born with gastroschisis on the 29th September and presented to your hospital on the 1st October you should select October.
Has consent been provided to include this patient in the study?<br>If no, which condition did the patient present with?	Yes / No / Patient consent is not required for this study at my institution<br><br>Oesophageal atresia / Congenital diaphragmatic hernia / Intestinal atresia / Gastroschisis / Exomphalos / Omphalocele / Anorectal malformation / Hirschsprung's Disease.<br>Please select all the conditions that the patient presented with. Do not select a condition which the patient has already received surgical treatment for previously.
**Demographics**	
Gestational age at birth	Number of weeks from the first day of the women's last menstrual cycle until birth. Round up or down to the nearest week.
Age at presentation (in hours)	We understand this information may be difficult to obtain - please be as accurate as you can. Please round to the nearest hour. This number may be very large for patients who have a delayed presentation - please still enter it. For neonates born within your centre please enter 0. Enter unknown if unknown.
Gender	Male / Female/ Ambiguous/ Unknown
Weight at presentation	In kilograms (kg) on the day of presentation. Please provide a value to 1 decimal place.
Does the patient have another anomaly in addition to the study condition?	Yes, Cardiovascular, Yes, Respiratory, Yes: Gastrointestinal, Yes: Neurological, Yes: Genito-urinary, Yes: Musculoskeletal, Yes: Down syndrome, Yes: Beckwith-Wiedemann syndrome, Yes: Cystic fibrosis, Yes: Chromosomal, Yes: Other, No<br>Select all that apply. Include all anomalies diagnosed at any stage up until 30-days post primary intervention or 30-days following presentation for those who didn't receive an intervention. If you suspect an associated anomaly, but it has yet to be diagnosed, select 'other'.
Distance from the patient's home to your hospital	In kilometres (km). Please round to the nearest kilometre. Please enter 0 if born in your hospital.
**Antenatal Care and Delivery**	
Antenatal ultrasound undertaken?<br><br>If the condition was diagnosed antenatally, at what gestational age?	Yes: study condition diagnosed, Yes: problem identified but study condition not diagnosed, Yes: no problem identified, No<br>Please round up to the nearest week. If the patient has more than one study condition, please note the gestational age at which one or more of the conditions was first diagnosed.
Mode of transport to hospital?<br><br>Where did the patient present from? If other, please specify.	Ambulance, Other transport provided by the health service, Patient's own transport, Born within the hospital<br>Home / Community Clinic / General Practice / District Hospital / Other / Unknown<br>District hospital includes: secondary level healthcare, provincial hospital, general hospital, general mission hospital or regional hospital. It has general anaesthesia and can provide general surgical care.
Type of delivery:	Vaginal (spontaneous), Vaginal (induced), Caesarean section (elective), Caesarean section (urgent/non-elective), Unknown. Vaginal delivery includes those requiring forceps and ventouse.
**Clinical condition and patient care**	
Was the patient septic on arrival?	Yes, no<br>Sepsis is SIRS (Systemic Inflammatory Response Syndrome) with a suspected or confirmed bacterial, viral, or fungal cause. SIRS is a response to a stimulus, which results in two or more of the following: temperature > 38.5°C or < 36°C, tachycardia*, bradycardia* in children < 1 year old, tachypnoea*, leukopenia or leucocytosis*, hyperglycaemia*, altered mental status, hyperlactaemia*, increased central capillary refill time >2 seconds. *Variables are defined as

For peer review only - http://bmjopen.bmj.com/site/about/guidelines.xhtml

If yes, were appropriate antibiotics administered?	values outside the normal range for age. Arrival is the time of birth for neonates born at your hospital.<br>Yes: within 1 hour of arrival, Yes: within the first day of arrival, No<br>Appropriate antibiotics are defined as either broad spectrum covering gram negative, gram positive and anaerobic bacteria OR antibiotics that are the standard empirical treatment for that condition according to local guidelines OR are based on sensitivities provided by a microbiology sample.
Was the patient hypovolaemic on arrival?<br><br><br>If yes, was an intravenous fluid bolus given?<br>If yes, how much intravenous fluid was given?	Yes/ No. Criteria for diagnosis include at least one of the following: prolonged central capillary refill time > 2 seconds, *tachycardia, mottled skin, *reduced urine output, cyanosis, impaired consciousness, *hypotension. *Variables are defined as values outside the normal range for age.<br>Yes: within 1 hour of arrival, Yes: on the first day of arrival, No<br>10 - 20mls/ kg, above 20mls/ kg<br>If less than 10mls/ kg was given please select 'no' for the question asking if intravenous fluid was given.
Was the patient hypothermic on arrival?<br><br>If yes, was the patient warmed on arrival to within a normal temperature range?	Yes/ No. Defined as < 36.5 degrees Celsius core temperature. Arrival is the time of birth for neonates born at your hospital.<br>Yes/ No. Only select yes if warming was commenced within 1 hour of arrival. Arrival is the time of birth for neonates born at your hospital.
Did the patient receive central venous access?<br><br><br><br>If yes, did the patient acquire central line sepsis?	Yes: umbilical catheter, Yes: peripherally inserted central catheter (PICC), Yes: percutaneously inserted central line with ultrasound guidance, Yes: surgically placed central line (open insertion), No.<br>Please select all that the patient received within 30-days of primary intervention or 30-days of presentation if no intervention was undertaken.<br>Yes: diagnosed clinically, Yes: confirmed on microbiology, No<br>Within 30-days of primary intervention or 30-days of presentation if no intervention was undertaken.
Time from arrival at your hospital to primary intervention in hours	(enter 0 if no intervention was undertaken)<br>**Primary intervention for each condition is defined as: Oesophageal atresia**; surgery, either temporising or definitive, to manage the oesophageal atresia and/ or tracheo-oesophageal fistula. **Congenital diaphragmatic hernia**; surgery to reduce the hernia and close the defect. **Intestinal atresia**; surgery, either temporising or definitive, to manage the obstruction including stoma formation and primary anastomosis. **Gastroschisis**; any procedure to either cover or reduce the bowel and/ or close the defect. This includes application of a silo (regardless of whether or not they go on to require surgery). It excludes initial covering of the bowel in a plastic covering (bag or cling film) prior to intervention. **Exomphalos**; surgery or application of topical treatment to the sac in patients managed conservatively (regardless of whether or not they go on to require surgery). **Hirschsprung's disease**; surgery, either temporising or definitive, or rectal/ distal bowel irrigation, laxatives or digital stimulation in patients managed conservatively. This does not include pre-operative washouts in patients planned to have surgery. **Anorectal malformation**; surgery, either temporising or definitive, or anal/ fistula dilatation in patients with a low anorectal malformation managed conservatively.
American Society of Anesthesiologists (ASA) Score at the time of primary intervention	1. Healthy person, 2. Mild systemic disease, 3. Severe systemic disease, 4. Severe systemic disease that is a constant threat to life, 5. A moribund patient who is not expected to survive without the operation, Not applicable - no intervention
What type of anaesthesia was used for the primary intervention?	General anaesthesia with endotracheal tube, General anaesthesia with laryngeal airway, Ketamine anaesthesia, Spinal/ caudal anaesthesia, Local anaesthesia only, No anaesthesia/ just analgesia, No anaesthesia/ no analgesia, Not applicable: no surgery or intervention undertaken.
Who undertook the anaesthetic for the primary intervention?	Anaesthetic doctor, Anaesthetic nurse, Medical officer, Surgeon, Other healthcare professional, No anaesthetic undertaken<br>If more than one of these personnel were present please select the most senior.
Who undertook the primary intervention?	Paediatric surgeon (or junior with paediatric surgeon assisting/ in the room), General surgeon (or junior with paediatric surgeon assisting/ in the room), Junior doctor, medical officer or other (without a paediatric or general surgeon assisting/ in the room), Trainee surgeon (without a paediatric or general surgeon assisting or in the room), Not applicable - no surgery or primary intervention undertaken.
Was a Surgical Safety Checklist used at the time of primary intervention?	Yes, No: but it was available, No: it was not available, Not applicable: a conservative primary intervention was undertaken, Not applicable: no surgery or primary intervention undertaken
Total duration of antibiotics following primary intervention	In days (including the day of surgery and the day antibiotics were stopped. Include intravenous and oral antibiotics).
Did the patient receive a blood transfusion?	Yes: not cross-matched, Yes: cross-matched, No: not required, No: it was required but not available. Within 30-days of primary intervention or 30-days of presentation if no intervention was undertaken.
Did the patient require ventilation?<br><br><br>If yes, for how long did the patient remain on ventilation?	Yes: and it was given, Yes: but it was not available, No<br>Within 30-days of primary intervention or 30-days of presentation if no intervention was undertaken. Please include all types of ventilation.<br>In days (include all days on ventilation within 30-days of primary intervention or 30-days of presentation if no intervention was undertaken).
Time to first enteral feed (post-primary intervention)	In days (include the day of primary intervention and the day of first enteral feed in the calculation). Enter 0 if enteral feeds were not commenced. Enter 999 if feeds were not stopped, for example in patients with Hirschsprung's Disease managed conservatively. Include all types of enteral feeding - oral, nasogastric, gastrostomy and other.
Time to full enteral feeds (post-primary intervention)	In days (enter 0 if the patient died before reaching full enteral feeds or 30 if the patient had not reached full enteral feeds at 30-days post primary intervention or 30-days following admission in

For peer review only - http://bmjopen.bmj.com/site/about/guidelines.xhtml

	patients who did not receive a primary intervention). Include all types of enteral feeding - oral, nasogastric, gastrostomy and other.
Did the patient require parenteral nutrition?<br><br>If yes, for how long did the patient receive parenteral nutrition?	Yes and it was given, Yes and it was sometimes available but less than required, Yes but it was not available, No<br>In days. Include all days that the patient received parenteral nutrition (any volume) up until 30-days post primary intervention or 30-days following presentation in patients who do not receive an intervention.
**Outcomes**	
Did the patient survive to discharge?<br><br><br>If the patient was discharged prior, were they still alive at 30-days following primary intervention?<br><br>If no, cause of death?	Yes/ No<br>Select yes if the patient was still alive in your hospital 30-days after primary intervention or 30-days after presentation in patients who do not receive a primary intervention.<br>Yes, No: not followed-up after discharge, Followed-up but not until 30-days post primary intervention<br>This can include all reliable communication with the patient/ patient's family including in person, via telephone and other.<br>Sepsis, Aspiration pneumonia, Respiratory failure, Cardiac failure, Malnutrition, Electrolyte disturbance, Haemorrhage, Lack of intravenous access, Hypoglycaemia, Recurrent tracheo-oesophageal fistula, Recurrent diaphragmatic hernia, Anastomotic leak, Ischaemic bowel, Ruptured exomphalos sac, Enterocolitis, Other. If other, please specify
Duration of hospital stay (days)	Please include the day of admission and the day of discharge in your calculation. For example, if a patient presented on 1st October and was discharged on the 5th October, their duration of hospital stay would be 5 days. If the patient died, please record the number of days from admission to death. Only include the duration of the primary admission, not subsequent admissions if the patient re-presented.
Did the patient have a surgical site infection?	Yes, No, Not applicable: no surgical wound<br>This is defined as one or more of the following within 30-days of surgery: 1) purulent drainage from the superficial or deep (fascia or muscle) incision, but not within the organ/ space component of the surgical site OR 2) at least two of: pain or tenderness; localised swelling; redness; heat; fever; AND the incision is opened deliberately to manage infection, spontaneously dehisces or the clinician diagnoses a SSI (negative culture swab excludes this criterion) OR 3) there is an abscess within the wound (clinically or radiologically detected).
Did the patient have a full thickness wound dehiscence?	Yes, No, Not applicable - no surgical wound.<br>This is defined as all layers of the wound opening within 30-days of surgery
Did the patient require a further unplanned intervention?	Yes - percutaneous intervention, Yes - surgical intervention, No, Not applicable - no primary intervention undertaken. Within 30-days of primary intervention. This does not include routine reduction and closure of the defect in neonates with gastroschisis receiving a preformed silo.
Was the patient followed up at 30-days post primary surgery or intervention to assess for complications?	Yes: reviewed in person, Yes: via telephone consultation, Yes: via other means, Yes: still an in-patient at 30-days, No: data is based on in-patient observations only, No: follow-up was done but prior to 30-days
If the patient had a complication, when was it diagnosed?	During the primary admission, As an emergency re-attender, At routine follow-up as an out-patient, Not applicable: no complications
What study condition does this patient have?	Oesophageal atresia, Congenital diaphragmatic hernia, Intestinal atresia, Gastroschisis, Exomphalos/ Omphalocele, Anorectal malformation, Hirschsprung's Disease<br>If the patient has presented for the first time with more than one of these conditions please select all that apply. If the patient presented on this occasion with one of these conditions, but previously had another condition managed then only select the condition they are presenting with on this occasion and enter that they have another anomaly in the demographics section above. For example, if the patient presents at 2-months with Hirschsprung's disease, but previously had a duodenal atresia repair please select Hirschsprung's disease here (not intestinal atresia) and tick in the section above that they have another gastrointestinal anomaly.

Data Quality

To ensure high quality of data, a detailed protocol for collaborators has been produced and published on the study website (www.globalpaedsurg.com) in 12 languages: English, French, Spanish, Portuguese, German, Italian, Chinese, Arabic, Korean, Lithuanian, Turkish and Russian. Clear and concise definitions have been provided for all data points on the protocol, on the data collection forms and within REDCap when entering the data. A study launch meeting was undertaken where the principal investigator presented the data collection process in detail, demonstrated use of REDCap and answered questions. This was recorded, circulated to all collaborators via email and placed on the website. A frequently asked questions document has been circulated via email and placed on the website. Two meetings were held by the principal investigator to detail the study, data collection process and answer questions amongst the country leads so they in turn can provide advice and support to local collaborators within their country. Again this was recorded, circulated and placed on the website.

A pilot study of the patient data collection form and institutional survey was undertaken by lead investigators to optimise the study design and to address any feasibility or other barriers to effective data collection and study completion across participating sites. The pilot study commenced on 1st

For peer review only - http://bmjopen.bmj.com/site/about/guidelines.xhtml

August 2018 for 30 days in English, Spanish and French by 41 collaborator colleagues. The data collection forms were amended following feedback to clarify terminology, add important missing variables or descriptions and correct any translation errors. All translated data collection forms, REDCap and study documentation has been checked and verified by a native speaker for accuracy.

Data Validation

Ten percent of collaborating centres will be selected at random for data validation by an independent research collaborator. The aim will be to determine the numbers of patients eligible during the data collection period to check if any were missed and collect a selection of data again to cross-check for accuracy. Validating questions have been built into the data collection tool. At least 90% of primary and secondary outcomes must be completed for each patient. All collaborators within validating centres will be asked to complete a brief survey regarding their experience with data collection to identify any potential errors and to aid with data interpretation.

Sample Size Calculation

A sample size calculation was undertaken using Stata/IC 15.0 based on Bonferroni correction for multiple testing, assuming 80% power and an overall type 1 error of 5%. The required sample size for each condition has been calculated for the primary outcome of mortality in LMICs compared to HICs and also low, middle and high-income countries separately **(Table 2).** Mortality estimations are based on pooled data from published studies on these conditions in low-, middle- and high-income countries respectively.

Based on the patient numbers included in the previously undertaken PaedSurg Africa study, which utilised a similar study design, the estimated sample sizes to detect a significant difference between LMICs and HICs in this study are achievable.[13]

Table 2. Estimated mortality and sample sizes for low, middle and high-income countries and the mean number of cases per month per institution globally

Condition	Mortality LIC (%, n)	Mortality MIC (%, n)	Mortality LMIC combined (%, n)	Mortality HIC (%, n)	Sample size for LIC	Sample size for MIC	Sample size for HIC	Sample size for LMIC vs HIC (per group)	Mean no. cases/ month/ institution (L,M&HIC combined)
OA +/- TOF	79.5% (62/78)	41.8% (623/1488)	43.7% (685/1566)	2.7% (6/221)	34	34	23	21	1.02
CDH	-	47.4% (130/274)	47.4% (130/274)	20.4% (201/982)	-	-	-	63	0.54
IA	42.9% (42/98)	40.0% (97/241)	41.0% (139/339)	2.9% (12/407)	6014	6014	25	24	0.63
Gastroschisis	83.1% (211/254)	42.6% (205/481)	56.6% (416/735)	3.7% (28/748)	29	29	24	15	0.85
Exomphalos	25.5% (41/161)	31.9% (132/414)	30.1% (173/575)	12.7% (40/316)	1040	1040	196	115	0.63
ARM	26.3% (26/99)	17.5% (243/1391)	18.1% (269/1490)	3% (14/462)	460	460	90	85	1.34
Hirschsprung's Disease	19.1% (33/173)	16.8% (55/328)	17.6% (88/501)	2.3% (43/1897)	5802	5802	85	79	2.21

Estimated Study Population

The mean number of cases presenting to an institution per month for each study condition was estimated from published studies across all income settings **(Table 2)**. On average most institutions caring for patients with these conditions receive 1-2 new cases per month; each participating institution would expect approximately 7-14 new cases in the study per month although this can vary. The aim is to include a minimum of 365 months of data; 183 months from LMICs and 183 months from HICs. This should ensure enough cases of exomphalos to determine a significant difference between LMICs and HICs; fewer months of data are required to determine significant differences between other study conditions. An up-to-date total of patient numbers within the study will be maintained on the study website.

For peer review only - http://bmjopen.bmj.com/site/about/guidelines.xhtml

Data Analysis

Patient and Institutional Data:

Data will be analysed using Stata and SAS 9.4 (Cary, NC; USA). Missing data for the covariates will be analysed to determine whether it is related to the outcome and either complete-case analyses or multiple imputation techniques will be used for analyses accordingly.

Significant differences in mortality between LMICs and HICs will be determined for each of the study conditions using Chi-squared analysis, or Fischer's exact test if either group contains less than 10 patients. World Bank classification of low-, middle- and high-income countries during the fiscal year 2018 will be used.[38]

Univariate logistic regression analyses will be conducted between covariates and the primary outcome of mortality. Based on the results, covariates with a p-value of <0.10 will be included in the multivariate model. The final multi-level multivariate logistic model will be determined using stepwise backward elimination to interventions and peri-operative factors affecting outcomes. Data will be adjusted for confounding factors and effect modifiers. Potential confounders include: gestation age at birth, weight, time from birth to presentation and ASA score at the time of primary intervention. Potential effect modifiers include: administration of peri-operative antibiotics, fluid resuscitation, thermal control and provision of other condition specific neonatal care such as parenteral nutrition in neonates with gastroschisis.

Multi-level multivariate logistic regression analysis will also be undertaken to identify institutional factors affecting mortality with adjustment for confounders. P< 0.05 will be deemed significant.

Data Validation:

A weighted kappa statistic will be utilised to determine the level of agreement between the patient data in the main study and the validation data. A weighted kappa statistic will be also utilised to determine the level of agreement between institutional surveys independently completed by the local study lead and one other consultant or registrar at each participating centre. Results will be presented as a proportion of agreement for each variable being validated.

Patient and Public Involvement

CDH UK, a patient and family advisory group and charity, provided input into the design of the study protocol and data collection tool. Their input will be sought on the findings and dissemination of the results.

ETHICS AND DISSEMINATION

Research Ethics Approval

The study has been classified as an audit at the host institution and hence did not require ethical approval. The study fulfils the audit criteria as follows: 1) All data collected measures current practice. The study does not involve any changes to patient management; 2) Current practice and outcomes in low, middle and high-income countries will be compared to published standards in the literature. **Table 2** details the current mortality standards for each of the seven study conditions in high-income countries; 3) All the study data is routinely collected information which should be known to the study team without asking additional questions to the patients/parents; 4) All data to be entered into REDCap is entirely anonymous; 5) No individual patient, collaborator, institution or country will be independently identifiable in the study results; 6) All data will be stored securely and will be governed by King's College London data protection team.

For peer review only - http://bmjopen.bmj.com/site/about/guidelines.xhtml
For peer review only

Research collaborators were required to gain approval to participate in the study at their institution according to their local ethical regulations. Data transfer agreements were legally signed between institutions where required. The participating institutions, type of study approval and study approval reference numbers are detailed in Supplementary File 3. It was not mandated for study approvals to be translated into English. Hence, some reference numbers are in the local scripture of the participating country and have therefore not been incorporated into the table.

Study Dissemination

The study concept and design will be presented at international conferences in order to recruit collaborators. Following completion, the results will be presented at local, national and international conferences globally. Both the promotional presentations of the study protocol and the study results will be presented by study collaborators of all levels of training, disciplines and regions of the world. The results will be submitted for open access publication in a peer reviewed journal. Following publication, the full anonymous, de-identified dataset will be made publicly available via an online repository. Collaborators will have the opportunity to undertake sub-analyses of the data for their country (if all collaborators from that country agree), region or continent.

DISCUSSION

This study aims to define, for the first time, the management and outcomes of a selection of common life-threatening congenital anomalies across the globe. This will help to raise awareness of the unacceptable disparities in outcomes between low-, middle- and high-income countries and the need to focus on improving access to quality surgical care for neonates with congenital anomalies within national health plans and global health prioritisation. It is hoped that factors affecting mortality and morbidity will be identified that can be modified to improve care. Establishment of the Global PaedSurg Research Collaboration developed during this study will create a platform for ongoing collaborative work and interventional studies aimed at improving outcomes in the future.

For peer review only

ADDITIONAL INFORMATION

Twitter: @GlobalPaedSurg

Website: http://globalpaedsurg.com

Author Contributions: The principal investigator conceived the idea for the study, gained study funding, wrote the study protocol, designed the data collection tools, established the study team, co-ordinated the pilot study, revised the study design/ data collection tools following feedback and made critical revisions to the manuscript for publication. The steering committee contributed critical input and revisions to the funding application, study design, protocol and manuscript for publication. The writing committee drafted the protocol manuscript for publication and contributed as organising committee members. The organising committee assisted in the recruitment of and communication with collaborators to participate in the pilot study, helped to co-ordinate the pilot study and summarise the feedback, made critical revisions to the data collection tools in multiple languages and contributed to the study design. The lead investigators contributed to the study design and content of the data collection forms through feedback following participation in the pilot study. All contributed to the content of this manuscript.

Funding Statement: Naomi Wright, Principal Investigator, is funded by the Wellcome Trust through a Clinical PhD in Global Health undertaken at King's College London (Funder Reference: 203905/Z/16/Z). The Wellcome Trust had no input into the study protocol other than to recommend open-access publication in a peer-reviewed journal and to make the anonymised dataset publicly available. Nick Sevdalis' (NS) research is supported by the National Institute for Health Research (NIHR) Collaboration for Leadership in Applied Health Research and Care South London at King's College Hospital NHS Foundation Trust. NS is a member of King's Improvement Science, which is part of the NIHR CLAHRC South London and comprises a specialist team of improvement scientists and senior researchers based at King's College London. Its work is funded by King's Health Partners (Guy's and St Thomas' NHS Foundation Trust, King's College Hospital NHS Foundation Trust, King's College London and South London and Maudsley NHS Foundation Trust), Guy's and St Thomas' Charity, the Maudsley Charity and the Health Foundation. NS and Andrew Leather are also supported by the NIHR Global Health Research Unit on Health System Strengthening in Sub-Saharan Africa, King's College London (GHRU 16/136/54) and by the ASPIRES research programme in LMICs (Antibiotic use across Surgical Pathways - Investigating, Redesigning and Evaluating Systems), funded by the Economic and Social Research Council. The views expressed are those of the authors and not necessarily those of the NHS, the NIHR or the Department of Health and Social Care.

Competing Interest's Statement: Nick Sevdalis is the director of the London Safety and Training Solutions Ltd, which offers training in patient safety, implementation solutions and human factors to healthcare organisations. No other conflicts of interest are declared.

Patient Consent: Collaborators must follow their local ethical guidelines regarding patient consent.

Ethics Approval: This study has been classified as a clinical audit with written confirmation from King's College London Ethics Committee that it does not therefore require ethical approval. All participating centres have gained study approval to participate according to their local institutional ethical regulations (Supplementary File 3).

Provenance and Peer Review: Not commissioned; externally peer reviewed.

Open Access: This is an Open Access article distributed in accordance with the terms of the Creative Commons Attribution (CC BY 4.0) license, which permits others to distribute, remix, adapt and build upon this work, for commercial use, provided the original work is properly cited.

Licence Statement: I, the Submitting Author has the right to grant and does grant on behalf of all authors of the Work (as defined in the below author licence), an exclusive licence and/or a non-exclusive licence for contributions from authors who are: i) UK Crown employees; ii) where BMJ has

For peer review only - http://bmjopen.bmj.com/site/about/guidelines.xhtml

agreed a CC-BY licence shall apply, and/or iii) in accordance with the terms applicable for US Federal Government officers or employees acting as part of their official duties; on a worldwide, perpetual, irrevocable, royalty-free basis to BMJ Publishing Group Ltd ("BMJ") its licensees and where the relevant Journal is co-owned by BMJ to the co-owners of the Journal, to publish the Work in BMJ Open and any other BMJ products and to exploit all rights, as set out in our licence.

The Submitting Author accepts and understands that any supply made under these terms is made by BMJ to the Submitting Author unless you are acting as an employee on behalf of your employer or a postgraduate student of an affiliated institution which is paying any applicable article publishing charge ("APC") for Open Access articles. Where the Submitting Author wishes to make the Work available on an Open Access basis (and intends to pay the relevant APC), the terms of reuse of such Open Access shall be governed by a Creative Commons licence – details of these licences and which Creative Commons licence will apply to this Work are set out in our licence referred to above.

Acknowledgements: Thank you to Bolaji Coker for the REDCap administration and management. Thank you to Beverley Power (CDH UK) for representing patients, parents and families through your feedback on the study design during the pilot study. Thank you to Xiya Ma and Dylan Goh for helping with the Chinese translation of study documentation.

COLLABORATORS

Principal Investigator: Naomi Wright (King's College London, UK)

Steering Committee: Niyi Ade-Ajayi (King's College Hospital, UK), Adesoji Ademuyiwa (Lagos University Teaching Hospital, Nigeria), Emmanuel Ameh (National Hospital, Abuja, Nigeria), Justine Davies (University of Birmingham, UK), Kokila Lakhoo (University of Oxford and Oxford University Hospitals, UK), Dan Poenaru (McGill University, Montreal, Canada), Nick Sevdalis (King's College London, UK), Emily Smith (Baylor University, Texas, USA), Andy Leather (King's College London, UK)

Writing Committee: Harmony Ubhi (King's College London, UK), Samuel Parker (Imperial College London, UK), Godfrey Sama Philipo (Muhimbili University of Health and Allied Sciences, Tanzania)

Organising Committee: Sadi Abukhalaf (Al Quds University, Palestine), Nana Adofo-Ansong (Mahikeng Provincial Hospital, South Africa), Melika Akhbari (King's College London, UK), Ahmad Alhamid (University of Aleppo, Syria), Osaid H. Alser (University of Oxford, UK), Emrah Aydin (Koc University, Turkey), Yousra-Imane Benaskeur (Universite de Montreal, Canada), Shrouk M. Elghazaly (Assiut University, Egypt), Safa abdal Elrais (University of Tripoli, Libya), Sophia Hashim (University College London, UK), Laura Herrera (Geisel School of Medicine, Dartmouth, USA), Gabriella Hyman (University of Witwatersrand, South Africa), Henang Kwasau (College of Medicine and Allied Health Sciences, University of Sierra Leone), Yang Liu (Children's Hospital, Zhejiang University School of Medicine, China), Bruno Martinez-Leo (Moctezuma Children's Hospital, Mexico), Kelly Naranjo (Columbia University Medical Centre, USA), Ibrahim Nour (Jordan University Hospital, Jordan), Cristiana Riboni (University of Pavia, Italy), Mahmoud Saleh (University of Gezira, Sudan), Hosni Khairy Salem (Cairo University, Egypt), Patricia Shinondo (University Teaching Hospital, Lusaka, Zambia), Marcus Sim (Stepping Hill Hospital, UK), Hannah Thompson (King's College Hospital, UK), Agota Vaitkiene (Vilnius University Hospital Santaros Kliniko, Lithuania), Dominique Vervoort (Harvard Medical School, USA), Isabelle Williams (Cambridge University, UK), Aayenah Yunus (King's College London, UK).

Lead Investigators: Muhammad Amjad Chaudhary, Adnan Ahmed Khan Khattak, Muhammad Bin Amjad (Children's Hospital, PIMS, Islamabad, Pakistan), Marlene Dominguez Anaya (Children's Hospital Manuel A.Villarroel Cochabamba, Bolivia), Samiul Hasan, Sabbir Karim, Ashrarur Rahman Mitul (Dhaka Shishu (Children) Hospital, Bangladesh), Paolo Bragagnini, Segundo Rite (Hospital Universitario Miguel Servent, Zaragoza), Hana Arbab, Lubna Samad, Aqil Soomro (Indus Hospital, Pakistan), Niveshni Maistry (John Radcliffe Hospital, UK), Raed Nael Al-Taher, Ibrahim Rabi Nour, Osama Abdul Kareem Sarhan (Jordan University Hospital, Jordan), Muhammad Arshad, Taimur

For peer review only - http://bmjopen.bmj.com/site/about/guidelines.xhtml

Qureshi, Hina Yousaf (Liaquat National Hospital, Pakistan), Candy SC Choo, Doris Mae Dimatatac, Shireen Anne Nah (KK Women's and Children's Hospital, Singapore), Vijay Anand Ismavel, Ann Miriam, Shajin T (Makunda Christian Leprosy and General Hospital, India), Monica Ivanov, Andreea Serban (Marie Curie Hospital in Bucharest, Romania), Bruno Martinez-Leo (Moctezuma Children's Hospital, Mexico), Eva Blazquez-Gomez, Luis Garcia-Aparicio, Martí Iriondo, Jordi Prat, Xavier Tarrado (Hospital Sant Joan de Deu, Spain), Lars Hagander, Emma Svensson (Skane University Hospital's Pediatric Care Hospital, Lund, Sweden), Alhassan Abdul-Mumin, Dominic Bagbio, Sheila Owusu, Stephen Tabiri (Tamale Teaching Hospital, Ghana), Dayang Anita Abdul Aziz (UKM Medical Centre, Kuala Lumpur, Malaysia).

For peer review only

REFERENCES

1. GBD Child Mortality Collaborators. Global, regional, national, and selected subnational levels of stillbirths, neonatal, infant, and under-5 mortality, 1980-2015: a systematic analysis for the Global Burden of Disease Study 2015. Lancet 2016; 388(10053): 1725-74.
2. Boyle B, Addor MC, Arriola L, et al. Estimating Global Burden of Disease due to congenital anomaly: an analysis of European data. Arch Dis Child Fetal Neonatal Ed 2017.
3. Flores A, Valencia D, Sekkarie A, et al. Building capacity for birth defects surveillance in Africa: Implementation of an intermediate birth defects surveillance workshop. J Glob Health Perspect 2015; 2015.
4. Sitkin NA, Ozgediz D, Donkor P, Farmer DL. Congenital anomalies in low- and middle-income countries: the unborn child of global surgery. World J Surg 2015; 39(1): 36-40.
5. Ozgediz D, Langer M, Kisa P, Poenaru D. Pediatric surgery as an essential component of global child health. Semin Pediatr Surg 2016; 25(1): 3-9.
6. Farmer D, Sitkin N, Lofberg K, Donkor P, Ozgediz D. Surgical Interventions for Congenital Anomalies. In: Debas HT, Donkor P, Gawande A, Jamison DT, Kruk ME, Mock CN, eds. Essential Surgery: Disease Control Priorities, Third Edition (Volume 1). Washington (DC); 2015.
7. Sakonidou S, Ali K, Farmer I, Hickey A, Greenough A. Mortality and short-term morbidity in infants with exomphalos. Pediatr Int 2018.
8. Ameh EA, Seyi-Olajide JO, Sholadoye TT. Neonatal surgical care: a review of the burden, progress and challenges in sub-Saharan Africa. Paediatr Int Child Health 2015; 35(3): 243-51.
9. Ekenze SO, Ajuzieogu OV, Nwomeh BC. Challenges of management and outcome of neonatal surgery in Africa: a systematic review. Pediatr Surg Int 2016; 32(3): 291-9.
10. Wright NJ, Zani A, Ade-Ajayi N. Epidemiology, management and outcome of gastroschisis in Sub-Saharan Africa: Results of an international survey. Afr J Paediatr Surg 2015; 12(1): 1-6.
11. PaedSurg Africa Research Collaboration. Paediatric Surgery across Sub-Saharan Africa: A Multi-Centre Prospective Cohort Study. https://clinicaltrials.gov/ct2/show/NCT03185637 (Accessed 26th January 2019).
12. Bradnock T, Marven S, Owen A, et al. Gastroschisis: one year outcomes from national cohort study. BMJ 2011; 343(d6749).
13. Krishnaswami S, Nwomeh BC, Ameh AE. The pediatric surgery workforce in low- and middle-income countries: problems and priorities. Semin Pediatr Surg 2016; 25(1): 32-42.
14. Okoye MT, Ameh EA, Kushner AL, Nwomeh BC. A pilot survey of pediatric surgical capacity in West Africa. World J Surg 2015; 39(3): 669-76.
15. United Nations. Sustainable Development Goals. 2015. http://www.un.org/sustainabledevelopment/health/ (accessed 27th February 2018).
16. Wright NJ, Anderson JE, Ozgediz D, Farmer DL, Banu T. Addressing paediatric surgical care on World Birth Defects Day. Lancet 2018; 391(10125): 1019.
17. GlobalSurg Collaborative. Mortality of emergency abdominal surgery in high-, middle- and low-income countries. Br J Surg 2016; 103(8): 971-88
18. GlobalSurg Collaborative. Surgical site infection after gastrointestinal surgery in high-income, middle-income, and low-income countries: a prospective, international, multi-centre cohort study. Lancet Infect Dis 2018; 18(5): 516-25.
19. GlobalSurg Collaborative. Laparoscopy in management of appendicitis in high-, middle-, and low-income countries: a multicenter, prospective, cohort study. Surg Endosc 2018.
20. GlobalSurg Collaborative. Determinants of morbidity and mortality following emergency abdominal surgery in children in low-income and middle-income countries. BMJ Glob Health 2016; 1(4): e000091.
21. Bhangu A, Kolias AG, Pinkney T, Hall NJ, Fitzgerald JE. Surgical research collaboratives in the UK. Lancet 2013; 382(9898): 1091-2.
22. Long AM, Bunch KJ, Knight M, Kurinczuk JJ, Losty PD, Baps C. Early population-based outcomes of infants born with congenital diaphragmatic hernia. Arch Dis Child Fetal Neonatal Ed 2018.
23. Ruttenstock E, Wright N, Barrena S, et al. Best oxygenation index on day 1: a reliable marker for outcome and survival in infants with congenital diaphragmatic hernia. Eur J Pediatr Surg 2015; 25(1): 3-8.
24. Burjonrappa S, Crete E, Bouchard S. Comparative outcomes in intestinal atresia: a clinical outcome and pathophysiology analysis. Pediatr Surg Int 2011; 27(4): 437-42.
25. Forrester MB, Merz RD. Structural birth defects associated with omphalocele and gastroschisis, Hawaii, 1986-2001. Congenit Anom (Kyoto) 2008; 48(2): 87-91.

For peer review only - http://bmjopen.bmj.com/site/about/guidelines.xhtml

26. Kunz SN, Tieder JS, Whitlock KJ, Jackson C, Avansino JR. Primary fascial closure versus staged closure with silo in patients with gastroschisis: a meta-analysis. J Pediatr Surg 2013; 48(4): 845-57.
27. Ross AR, Eaton S, Zani A, Ade-Ajayi N, Pierro A, Hall NJ. The role of preformed silos in the management of infants with gastroschisis: a systematic review and meta-analysis. Pediatr Surg Int 2015; 31(5): 473-83.
28. Levitt MA, Pena A. Anorectal malformations. Orphanet J Rare Dis 2007; 2: 33.
29. Van der Steeg HJ, Schmiedeke E, Bagolan P, et al. European consensus meeting of ARM-Net members concerning diagnosis and early management of newborns with anorectal malformations. Tech Coloproctol 2015; 19(3): 181-5.
30. Holschneider A, Hutson J, Pena A, et al. Preliminary report on the International Conference for the Development of Standards for the Treatment of Anorectal Malformations. J Pediatr Surg 2005; 40(10): 1521-6.
31. Bradnock TJ, Knight M, Kenny S, Nair M, Walker GM, British Association of Paediatric Surgeons Congenital Anomalies Surveillance S. Hirschsprung's disease in the UK and Ireland: incidence and anomalies. Arch Dis Child 2017; 102(8): 722-7.
32. Ross AR, Hall NJ. Outcome reporting in randomized controlled trials and systematic reviews of gastroschisis treatment: a systematic review. J Pediatr Surg 2016; 51(8): 1385-9.
33. Allin BSR, Hall NJ, Ross AR, et al. Development of a gastroschisis core outcome set. Arch Dis Child Fetal Neonatal Ed 2018.
34. Allin BS, Irvine A, Patni N, Knight M. Variability of outcome reporting in Hirschsprung's Disease and gastroschisis: a systematic review. Sci Rep 2016; 6: 38969.
35. Watanabe S, Suzuki T, Hara F, Yasui T, Uga N, Naoe A. Omphalocele and Gastroschisis in Newborns: Over 16 Years of Experience from a Single Clinic. J Neonatal Surg 2017; 6(2): 27.
36. Tan KB, Tan KH, Chew SK, Yeo GS. Gastroschisis and omphalocele in Singapore: a ten-year series from 1993 to 2002. Singapore Med J 2008; 49(1): 31-6.
37. Schneider A, Blanc S, Bonnard A, et al. Results from the French National Esophageal Atresia register: one-year outcome. Orphanet J Rare Dis 2014; 9: 206.
38. World Bank. World Bank Country and Lending Groups. 2018. https://datahelpdesk.worldbank.org/knowledgebase/articles/906519-world-bank-country-and-lending-groups (Accessed 26th January 2019).

For peer review only

Management and Outcomes of Gastrointestinal Congenital Anomalies in Low-, Middle- and High-Income Countries: Protocol for a Multi-Centre, International, Prospective Cohort Study

SUPPLEMENTARY FILE 1: Condition Specific Data Points

Oesophageal Atresia (OA) +/- Tracheo-Oesophageal Fistula (TOF)

Question	Answers
Type of OA +/- TOF (Gross classification)	A, B, C, D, E<br>A: without a fistula, B: proximal TOF with distal OA, C: distal TOF with proximal OA, D: proximal and distal TOF, E: H-type TOF without OA.
Long or short gap?	Long, Short, Unknown<br>Long gap OA: A gap of 4 vertebral bodies or more. Anatomically cases either have no TOF or a gap of over 4 vertebral bodies following division of the distal fistula making primary repair unfeasible. Short OA: A gap of less than 4 vertebral bodies. Primary anastomosis typically feasible.
Pneumonia at presentation?	Yes: diagnosed clinically, Yes: diagnosed radiologically, Yes: other means of diagnosis, No: patient born in the study centre, No: patient born outside the study centre but no evidence of pneumonia on arrival<br>Pneumonia is defined as lung inflammation typically caused by bacterial or viral infection, in which the air sacs fill with pus and may become solid.
Primary intervention:	TOF ligation, Oesophageal anastomosis, Oesophagostomy, Gastrostomy, Ligation of the distal oesophagus, Gastro-oesophageal disconnection, Foker technique, Fundoplication, Other (please specify), Palliative care<br>Select all that apply.
If the patient had a primary oesophageal anastomosis, was a post-operative oesophagogram undertaken?	Yes, No. (At any stage)
If yes, routine or clinically indicated?	Routine, Clinically indicated
If yes, when?	Number of days after primary surgery
If yes, what was the result?	Leak, No leak
For patients diagnosed with a leak radiologically, was it associated with clinical symptoms?	Yes, No
Time to first oral feed post-operatively	In days. Please include the day of surgery and the first day of oral feeds in the calculation. Enter 0 if oral feeds were not commenced within 30-days of primary intervention. Do not include other types of enteral feeding such as nasogastric or gastrostomy feeding.
Time to full oral feeds	In days (enter 0 if the patient died before reaching full oral feeds or 30 if the patient had not reached full oral feeds at 30-days post primary intervention). Do not include other types of enteral feeding such as nasogastric or gastrostomy feeding.
For patient's not receiving a primary oesophageal anastomosis, at what age is definitive surgery planned?	In months (enter unknown if not planned or enter not applicable if primary anastomosis was undertaken).
For patient's not receiving a primary oesophageal anastomosis, what is the future planned procedure?	Gap assessment, Primary oesophageal anastomosis if possible, Gastric pull-up, Jejunal interposition, Colonic interposition, Not applicable: primary anastomosis undertaken, Other, Unknown. Select all that apply. If other, please specify.
If the patient had surgery, what was the approach?	Thoracotomy muscle cutting, Thoracotomy muscle splitting, Thoracoscopy, Laparotomy, Laparoscopy, Limited local incision, Other.<br>During primary surgery. If other, please specify.
If thoracoscopic or laparoscopic, was the surgery converted to open?	Yes, No
Did the patient have a condition specific complication within 30-days of primary intervention?	Pneumonia, Mediastinitis, Pneumothorax, Chylothorax, Haemothorax, Anastomotic leak, Anastomotic stricture, Recurrent TOF, Other, None<br>Select all that apply. If other, please specify.
Did the patient have tracheomalacia?	Yes: diagnosed clinically, Yes: diagnosed on bronchoscopy, Yes: diagnosed on CT, Yes: diagnosed on bronchogram, Yes: other method of diagnosis, No
If yes, was an intervention undertaken? If other, please specify	Yes: aortopexy, Yes: tracheostomy, Yes: tracheal stent, Yes: supportive management (oxygen +/- ventilation) only, Yes: other treatment, No

Congenital Diaphragmatic Hernia (CDH)

Question	Answers
Type of CDH.	Left posteriolateral (Bochdalek), Right posteriolateral (Bochdalek), Bilateral posteriolateral (Bochdalek), Central, Anterior (Morgagni), Other. If other, please specify.
Type of Bochdalek CDH (CDH Study Group Classification)	A, B, C, D, Other (specify), Unknown.<br>Defect A: smallest defect, usually "intramuscular" defect with >90% of the hemi-diaphragm present; this defect involves < 10% of the circumference of the chest wall. Defect B: 50-75% hemi-diaphragm present; this defect involves < 50% of

For peer review only - http://bmjopen.bmj.com/site/about/guidelines.xhtml

If bilateral, what was the type of Bochdalek hernia on the left?<br>If bilateral, what was the type of Bochdalek hernia on the right?	the chest wall. Defect C: < 50% hemi-diaphragm present; this defect involves >50% of the chest wall. Defect D: largest defect (previously known as "agenesis"); complete or near complete absence of the diaphragm with < 10% hemi-diaphragm present; this defect involves >90% of the chest wall. Surgically, it is an absent posterior rim beyond the spine, absent posterior-lateral rim, and an anterior/anterior-medial rim which is miniscule. As it is truly unusual to have zero tissue at all, this is the CDHSG member consensus. "D" defects should all require a patch (or muscle flap) for repair.<br>A, B, C, D, Other, Unknown<br>If other, please specify.<br>A, B, C, D, Other, Unknown<br>If other, please specify.
If antenatally diagnosed, what was the lung-to-head ratio (LHR)?	Enter zero if not undertaken/ not known.
Was foetal tracheal occlusion (FETO) undertaken?<br>If yes, at what gestational age was it inserted?<br>If yes, at was gestational age was it removed?	Yes, No<br>__________, unknown.<br>__________, at birth, unknown.
Liver position?	Chest, Abdomen, Unknown
Did the patient have pulmonary hypertension (at any stage)?<br>If yes, treatment given? If other, please specify.	Yes: diagnosed clinically, Yes: diagnosis confirmed on echocardiography, Yes: other method of confirming diagnosis, No, Unknown<br>Persistent pulmonary hypertension of the newborn (PPHN) is defined as the failure of the normal circulatory transition that occurs after birth. It is a syndrome characterised by marked pulmonary hypertension that causes hypoxemia secondary to right-to-left extrapulmonary shunting of deoxygenated blood. It should be suspected whenever the level of hypoxemia is out of proportion to the level of pulmonary disease. Echocardiography plays a major role in screening and assisting in making the diagnosis of PPHN.<br>Nitric oxide, Prostacyclin, Alprostadil, Milrinone, Other, None: not required, None: required but not available.
Did the patient receive extracorporeal membrane oxygenation (ECMO)?<br>If yes, for how long?	Yes, No<br>In days. Include the day the patient went onto ECMO and the day they were taken off in the calculation.
Primary intervention<br>If patch repair, material used?<br>Other procedures undertaken at the same time?<br>Surgical approach:<br>If laparoscopic or thoracoscopic, was the surgery converted to open?<br>Condition specific complication within 30-days of primary surgery?	Primary repair (absorbable sutures), Primary repair (non-absorbable sutures), Patch repair, Palliation, Discharged with planned elective repair, Other<br>Permacol, PTFE, Alloderm, Dacron, Mesh plug, Muscle flap, Surgisis, Other. If other, please specify.<br>Chest drain insertion, Abdominal wall patch, Fundoplication, Correction of malrotation, Appendicectomy, Other (specify), None<br>Select all that apply. If other, please specify.<br>Laparotomy, Laparoscopy, Thoracotomy, Thoracoscopy, Other (please specify)<br>Yes/No.<br>Air leak (not just redundant space in the pleural cavity which is common), Chylothorax, Recurrence, Adhesional obstruction, Other, None. Select all that apply. If other, please specify.

Intestinal Atresia

**Question**	**Answers**
Type of intestinal atresia	Duodenal, Jejuno-ileal, Colonic
Classification of duodenal or colonic atresia<br>Classification of jejuno-ileal atresia	1,2,3,4<br>1) intraluminal web with continuity of the muscular layer, 2) atretic segment without a mesenteric defect, 3) atretic segment with mesenteric defect, 4) multiple atresias = string of sausages appearance.<br>1,2,3a,3b,4<br>1) intraluminal web with continuity of the muscular layer, 2) atretic segment without a mesenteric defect, 3a) atretic segment with mesenteric defect, 3b) apple-peel (bowel wrapped around a single artery), 4) multiple atresias = string of sausages appearance.
Primary intervention for duodenal atresia:<br>Surgical approach<br>Conversion to open procedure?<br>Type of anastomosis<br>Primary intervention for jejuno-ileal and colonic atresia:<br>If bowel was excised, what was the total length of bowel excised?<br>Surgical approach:<br>Conversion to open procedure?<br>Was the distal bowel flushed to check for patency?	Duodenoduodenostomy, Duodenojenunostomy, Web excision only, Palliation, Other. If other, please specify.<br>Laparotomy, Laparoscopy, Endoscopy, Other<br>Yes/ No<br>Kimura's diamond shape, Side-to-side, End-to-end<br>Primary anastomosis, Bowel resection, Division of web only, Loop stoma, Divided stoma, Bishop-Koop stoma, Santulli stoma, Palliation, Other. Select all that apply.<br>In centimetres (cm). Enter 0 if unknown<br>Laparotomy, Laparoscopy, Endoscopy, Other<br>Yes, No<br>Yes, No

For peer review only

If the patient underwent surgery, did they have a condition specific complication within 30-days of primary intervention?	Anastomotic leak, Anastomotic stenosis, Short-gut, Missed additional atresia, Adhesive bowel obstruction, Stoma prolapse, Stoma retraction, Parastomal hernia, Parastomal skin breakdown, Other. If other, please specify. Select all that apply. For the purposes of this study short gut is defined as more than 50% of the small intestine excised (when short bowel syndrome can occur).

Gastroschisis

Question	Answers
Type of gastroschisis	Simple, Complex: associated with atresia, Complex: associated with necrosis, Complex: associated with perforation, Complex: associated with closing gastroschisis. Select all that apply.
Primary intervention:	Primary closure in the operating room (OR), Primary closure at the cotside (Bianchi technique), Staged closure using a preformed silo, Staged closure using an Alexis Wound Retractor and Protector, Staged closure using a surgical silo (including improvised silo), Other method, No intervention undertaken. If other, please specify.
Method of defect closure:	Fascia and skin closed with sutures, Just skin closed with sutures: fascia left open, Umbilical cord sutured over the defect: fascia left open, Sutureless closure with skin edges opposed and dressing applied, Dressing applied: defect left open to close by secondary intention, Other, Patient died before the defect was closed. If other, please specify.
On what day following admission was abdominal wall closure achieved?	In days. Please include the first day of admission and the day of closure in the calculation. For example, for a neonate admitted with gastroschisis on 2nd October who had the defect closed on 4th October, please insert 3 days.
Did the neonate have any of these complications within 30-days of primary intervention?	Ischemic bowel, Abdominal compartment syndrome (ACS), Necrotising enterocolitis, None of these Select all that apply. ACS is defined as respiratory insufficiency secondary to compromised tidal volumes, decreased urine output caused by falling renal perfusion or any other organ dysfunction caused by increased intra-abdominal pressure.
If the patient has ACS, was the abdomen re-opened?	Yes/ No

Exomphalos

Question	Answers
Type of Exomphalos?	Major, Minor Major: >50% of the liver in the exomphalos sac and abdominal wall defect >5cm. Minor: Infants with defects less than 5cm.
Hypoglycaemic on arrival?	Yes, No, Blood glucose not measured Hypoglycaemia is defined as a blood glucose level below 4 mmol/L (72mg/dL).
Primary intervention	Primary operative closure, Staged closure, Conservative management
If the patient had a staged closure, what was the time from primary intervention to closure?	In days. Please include the day of the primary intervention and the day of closure in the calculation. Enter 30 if still not closed at 30-days after primary intervention.
If conservative management, was a topical treatment applied to the exomphalos sac?	Yes: silver sulfadiazine, Yes: betadine, Yes: honey, Yes: merbromide tannage, Yes: other, no. If other, please specify.
If conservative management was undertaken, what is the plan for future management?	No further surgery planned, Delayed closure at this hospital, Delayed closure at another hospital, Other. If other, please specify.
Did the patient have a ruptured sac?	Yes, No

Anorectal Malformation (ARM)

Question	Answers
Type of anorectal malformation (Krickenbeck classification)	Low ARM: Perineal (cutaneous) fistula, High ARM: Rectourethral fistula (bulbar), High ARM: Rectourethral fistula (prostatic), High ARM: Rectovesical fistula, High ARM: Vestibular fistula, High ARM: Cloaca, High ARM: No fistula, High ARM: Type unknown at present, Rare variant: Pouch colon, Rare variant: Rectal atresia/ stenosis, Rare variant: Rectovaginal fistula, Rare variant: H fistula, Other
Did the neonate have pre-operative bowel perforation?	Yes, No
What was the primary intervention undertaken?	Fistula dilation: no surgery, Loop sigmoid colostomy, Divided sigmoid colostomy, Loop transverse colostomy, Divided transverse colostomy, Other stoma, Anoplasty, Posterior sagittal anorectoplasty (PSARP), Abdominosacroperineal pull-through, Abdominoperineal pull-through, Laparoscopic-assisted pull-through, Palliative care, Other. If other, please specify. Select all that apply.
If primary anorectal reconstruction was undertaken, was a Peña stimulator or equivalent used to identify the position of the muscle complex intra-operatively?	Yes, no: equipment was not available, no: the equipment was available but not used. Peña stimulator: Muscle locating stimulator commonly used to identify the anal sphincter muscles whilst undertaking a PSARP for patients with ARM.
Did the patient have any of the following complications within 30-days of surgery? - Electrolyte disturbance - High output stoma (over 20mls/kg/day)	For each of the below answer: Yes, No, Not applicable

For peer review only - http://bmjopen.bmj.com/site/about/guidelines.xhtml

- Stoma prolapse/ retraction/ herniation<br>- Peri-stoma skin breakdown (or perianal if primary reconstructive surgery undertaken without a covering stoma)<br>- Anal stenosis in those undergoing primary anorectal reconstruction without covering stoma.<br>What is the plan for future management?	No further operative management, Anoplasty/ pull-through planned at your hospital, Anoplasty/ pull-through planned at another hospital, Stoma closure planned at your hospital, Stoma closure planned at another hospital, Other<br>Please tick all that apply. If other, please specify.

Hirschsprung's Disease

Question	Answers
Time to first passage of meconium after birth	Less than 24 hours, 24-48 hours, Over 48 hours, Unknown
Features at presentation:	Abdominal distension, Bilious vomiting, Non-bilious vomiting, Poor feeding, Suspected enterocolitis, Perforation, Other. Select all that apply.
Source of diagnosis of Hirschsprung's disease<br><br>If on biopsy, what was the method of histology staining.	Genetic, Mucosal biopsy, Full thickness biopsy, Anorectal manometry, Barium enema, Not confirmed: suspected only, Other.<br>Hemotoxilin and Eosin (H&E), Acetylcholinesterase, Calretinin, Other.<br>Select all that apply. If other, please specify.
Length of aganglionosis:	Rectal, Sigmoid, Descending colon, Transverse colon, Ascending colon, Small bowel, Unknown at present
Primary intervention<br><br><br><br><br><br><br>If primary pull-through was undertaken, did the patient have a covering stoma?<br>Was it laparoscopic assisted?<br>Did the patient have any condition specific complications within 30-days of primary intervention?<br><br><br><br><br><br><br><br><br>What is the plan for future management?	Conservative: no treatment, Conservative: digital stimulation and laxatives, Conservative: regular rectal washouts/ enemas, Failed conservative management followed by a stoma during the same hospital admission, Primary stoma (with or without pre-operative washouts or enemas prior to a planned stoma placement), Primary pull-through (Swenson), Primary pull-through (Duhamel), Primary pull-through (Soave), Primary pull-through (Other), Transanal posterior anorectal myectomy, Palliative care, Other.<br>Yes, No<br><br>Yes, No<br>Hirschsprung's associated enterocolitis (HAEC), Electrolyte disturbance, High stoma output (over 20mls/kg/day), Stoma prolapse/ retraction/ herniation, Peri-stoma skin breakdown (or perianal if primary pull-through was undertaken without a covering stoma), Anal stenosis, Post-operative obstruction, Anastomotic leak (if primary pull-through was undertaken without a covering stoma), Other<br>HAEC is defined as inflammation of the small and or large bowel in patient's born with Hirschsprung's disease. If the patient was managed conservatively, please tick if they developed enterocolitis within 30-days of presentation. Select all that apply.<br>No further surgery planned, Anorectal pull-through at your hospital, Anorectal pull-through at a different hospital, Stoma closure, Other, Unknown

For peer review only

Management and Outcomes of Gastrointestinal Congenital Anomalies in Low-, Middle- and High-Income Countries: Protocol for a Multi-Centre, International, Prospective Cohort Study

SUPPLEMENTARY FILE 2

Collaborator Survey: Resources and Facilities for Neonatal and Paediatric Surgery

Question	Answers
Title	Professor, Dr, Mr, Mrs, Miss, Ms, Other
Surname	
First Name	
Professional Position	Professor, Consultant or attending, Registrar or resident, Intern/ house officer/ senior house officer, Medical Student, Nurse, Other
Are you the study lead at your centre?	Yes, No<br>Answers provided by the study lead will be used as the 'Gold Standard' to which the other survey will be compared (this will remain anonymous).
Speciality	General Surgery (adult and paediatric), Paediatric Surgery, Anaesthetics, Paediatrics, Neonatology, Nursing, Not specialised yet, Other
Full name of institution:	
Address of institution:	
Country	Afghanistan, Albania, Algeria, Andorra, Angola, Antigua and Barbuda, Argentina, Armenia, Aruba (Kingdom of the Netherlands), Australia, Austria, Azerbaijan, Bahamas, Bahrain, Bangladesh, Barbados, Belarus, Belgium, Belize, Benin, Bhutan, Bolivia, Bosnia and Herzegovina, Botswana, Brazil, Brunei Darussalam, Bulgaria, Burkina Faso, Burundi, Cambodia, Cameroon, Canada, Cape Verde, Central African Republic, Chad, Chile, China, Colombia, Comoros, Congo, Costa Rica, Cote d'Ivoire, Croatia, Cuba, Curacao (Kingdom of the Netherlands), Cyprus, Czech Republic, Democratic Peoples Republic of Korea, Democratic Republic of the Congo, Denmark, Djibouti, Dominica, Dominican Republic, Ecuador, Egypt, El Salvador, Equatorial Guinea, Eritrea, Estonia, Eswatini (Swaziland), Ethiopia, Fiji, Finland, France, Gabon, Gambia, Georgia, Germany, Ghana, Greece, Grenada, Guatemala, Guinea, Guinea-Bissau, Guyana, Haiti, Holy See (Vatican City State), Honduras, Hong Kong (China), Hungary, Iceland, India, Indonesia, Iran (Islamic Republic of), Iraq, Ireland, Israel, Italy, Jamaica, Japan, Jordan, Kazakhstan, Kenya, Kiribati, Korea (Republic of), , Kuwait, Kyrgyzstan, Lao People's Democratic Republic, Latvia, Lebanon, Lesotho, Liberia, Libya, Liechtenstein, Lithuania, Luxembourg, Macau (China), Macedonia (the former Yugoslav Republic of), Madagascar, Malawi, Malaysia, Maldives, Mali, Malta, Marshall Islands, Mauritania, Mauritius, Mexico, Micronesia (Federated States of), Moldova (Republic of), Monaco, Mongolia, Montenegro, Morocco, Mozambique, Myanmar, Namibia, Nauru, Nepal, Netherlands, New Zealand, Nicaragua, Niger, Nigeria, Niue, Norway, Oman, Pakistan, Palau, Palestinian Territories, Panama, Papua New Guinea, Paraguay, Peru, Philippines, Poland, Portugal, Qatar, Republic of Kosovo, Reunion Island, Romania, Russian Federation, Rwanda, Saint Kitts and Nevis, Saint Lucia, Saint Vincent and the Grenadines, Samoa, San Marino, Sao Tome and Principe, Saudi Arabia, Senegal, Serbia, Seychelles, Sierra Leone, Singapore, Sint Maarten (Kingdom of the Netherlands), Slovakia, Slovenia, Solomon Islands, Somalia, Somaliland, South Africa, South Sudan, Spain, Sri Lanka, Sudan, Suriname, Swaziland (See Eswatini), Sweden, Switzerland, Syrian Arab Republic, Taiwan, Tajikistan, Tanzania (United Republic of), Thailand, Timor-Leste, Togo, Tonga, Trinidad and Tobago, Tunisia, Turkey, Turkmenistan, Tuvalu, Uganda, Ukraine, United Arab Emirates, United Kingdom, Uruguay, USA, Uzbekistan, Vanuatu, Venezuela, Viet Nam, Yemen, Zambia, Zimbabwe
Type of institution (WHO classification)	- **Specialised children's hospital** (Provides highly specialised care dedicated to children)<br>- **Referral hospital** (WHO defined tertiary healthcare. Includes academic, university, teaching, national, central and specialised mission hospitals. Can provide specialised surgical services)<br>- **District hospital** (WHO defined secondary healthcare. Includes provincial, general, general mission or regional hospitals. Has general anaesthesia and can provide general surgical care)<br>- **Health centre** (WHO defined primary healthcare. No general anaesthesia, can do minor local procedures, wound management, triage and referral).

For peer review only - http://bmjopen.bmj.com/site/about/guidelines.xhtml

Institutional classification	Government, Non-government
Institutional financial classification	Not for profit, For profit
Population served by your institution (in millions, including children and adults)	
**Personnel**	
Number of Consultant Paediatric Surgeons undertaking neonatal surgery at your institution:	____________________ (Excluding trainees)
Number of Consultant General Surgeons (covering adults and children) undertaking neonatal surgery independently at your institution:	____________________ (Excluding trainees)
Number of medical officers or other non-surgeon healthcare professionals undertaking neonatal surgery independently at your institution	____________________ (Without a consultant surgeon present at the time of surgery)
**Infrastructure**<br>Please state whether the following facilities are available at your institution when required:	
Running Water	Always, Sometimes, Never
Electricity	Always, Sometimes, Never
Electricity generator back-up	Always, Sometimes, Never
Laboratory for biochemistry	Always, Sometimes, Never
Laboratory for haematology	Always, Sometimes, Never
Blood bank	Always, Sometimes, Never
Sterile gloves and gown	Always, Sometimes, Never
Autoclave for sterilising surgical equipment	Always, Sometimes, Never
Neonatal ventilation outside the operating room	Always, Sometimes, Never
Paediatric ventilation outside the operating room	Always, Sometimes, Never
Neonatal intensive care unit for surgical neonates pre and post operatively (including if a stoma is present)	Always, Sometimes, Never
Paediatric intensive care unit for surgical paediatric patients pre and post operatively if required	Always, Sometimes, Never
Parenteral nutrition for neonates	Always, Sometimes, Never
Parenteral nutrition for adults and older children	Always, Sometimes, Never
Extracorporeal membrane oxygenation (ECMO)	Always, Sometimes, Never
Peña stimulator or equivalent device to identify the muscle complex during anorectal reconstruction	Always, Sometimes, Never
Suction rectal biopsy gun to investigate for Hirschsprung's disease	Always, Sometimes, Never
**Procedures**<br>Please state whether the following procedures are available at your institution when clinically appropriate/ required:	
Neonatal laparotomy	Always, Sometimes, Never
Neonatal laparoscopy	Always, Sometimes, Never
Neonatal thoracotomy	Always, Sometimes, Never
Neonatal thoracoscopy	Always, Sometimes, Never
Neonatal central line insertion	Always, Sometimes, Never
Paediatric central line insertion	Always, Sometimes, Never
Umbilical vein catheterisation	Always, Sometimes, Never
Bedside primary reduction and closure of gastroschisis (Bianchi technique)	Always, Sometimes, Never
Preformed silo application, reduction and closure of gastroschisis	Always, Sometimes, Never
Surgical silo application, reduction and closure of gastroschisis	Always, Sometimes, Never
Primary closure of gastroschisis in the operating room	Always, Sometimes, Never
Sigmoid colostomy	Always, Sometimes, Never
Posterior Sagittal Anorectoplasty (PSARP) for anorectal malformation	Always, Sometimes, Never
Foetal tracheal occlusion (FETO) for CDH	Always, Sometimes, Never
Pull-through for Hirschsprung's disease	Always, Sometimes, Never
**Anaesthesia and resuscitation**<br>Please state whether the following facilities are available at your institution when required:	
Neonatal bag, valve and mask	Always, Sometimes, Never
Paediatric bag, valve and mask	Always, Sometimes, Never
Bottled oxygen	Always, Sometimes, Never
Piped oxygen	Always, Sometimes, Never
Oxygen saturation monitor	Always, Sometimes, Never
Apnoea monitor	Always, Sometimes, Never
Multi-parameter intra-operative monitoring	Always, Sometimes, Never
Anaesthetic machine for neonates	Always, Sometimes, Never
Anaesthetic machine for children	Always, Sometimes, Never
Ketamine anaesthesia for neonates	Always, Sometimes, Never
Ketamine anaesthesia for children	Always, Sometimes, Never
Spinal/ caudal anaesthesia for neonates	Always, Sometimes, Never
Spinal/ caudal anaesthesia for children	Always, Sometimes, Never

For peer review only

Anaesthetic doctor competent to perform neonatal anaesthesia	Always, Sometimes, Never
Anaesthetic doctor competent to perform paediatric anaesthesia	Always, Sometimes, Never
Anaesthetic nurse competent to perform neonatal anaesthesia	Always, Sometimes, Never
Anaesthetic nurse competent to perform paediatric anaesthesia	Always, Sometimes, Never
Does your country have at least one specialised children's hospital that can provide neonatal and paediatric surgery?	Yes, No
Any other comments?	

For peer review only

Management and Outcomes of Gastrointestinal Congenital Anomalies in Low-, Middle- and High-Income Countries: Protocol for a Multi-Centre, International, Prospective Cohort Study

SUPPLEMENTARY FILE 3: Study approval details of participating centres

Number	Country	Institution Name	Ethical/Audit Review Board Name	Approval Type	Approval Reference Number
**Low-Income Countries (LICs)**					
1	Afghanistan	Ataturk National Children's Hospital	Islamic Republic of Afghanistan Ministry of Public Health, Afghanistan National Public Health Institute, Institutional Review Board	Local (Ethical)	444738
2	Afghanistan	French Medical Institute for Mothers and Children (FMIC)	Institutional Review Board	Local (Ethical)	-
3	Afghanistan	Ghalib University Hospital	Ministry of Higher Education, Ghalib Cadre Hospital	Local (Ethical)	-
4	Afghanistan	Ghor Provincial Hospital	EPHS Directorate, GD of Curative Medicine, Ministry of Public Health	Local (Ethical)	-
5	Afghanistan	Indra Ghandi Institute of Child Health (IGICH)	Institutional Review Board	Local (Ethical)	-
6	Afghanistan	Irena Salimi Children Hospital (ISCH)	Institutional Review Board	Local (Ethical)	-
7	Burundi	Bujumbura, Kamenge Military Hospital	Head of Department Surgery	Local (Departmental)	-
8	Burundi	District Hospital Kibimba, Gitega	Medical Director of Kibimba Hospital	Local (Hospital)	4000342958
9	Burundi	Gitega Hospital Mutoyi	Hospital Director	Local (Hospital)	-
10	Burundi	Kibuye Hope Hospital, Hope Africa University, Gitega	Head of Surgery and Ethics Team	Local (Ethical and Hospital)	-
11	Burundi	King Khaled Teaching Hospital	Medical Director General	Local (Hospital)	2019|DECHUK.359|11.5
12	Burundi	Teaching Hospital University of Kamenge	Medical Director General	Local (Hospital)	2019|DECHUK.271|11.5
13	Burundi	Van Norman Clinic Bjumbura	Medical Director of Kibimba Hospital	Local (Hospital)	-
14	Democratic Republic of Congo	HEAL Africa Hospital, Goma	Institutional Review Board at HEAL Africa	Local (Ethical)	-
15	Ethiopia	Addis Ababa University	Deputy Head of Department of Surgery	Local (Departmental)	-
16	Gambia	Edward Francis Small Teaching Hospital, Banjul	Chair of Gambia Government/MRCG Joint Ethics Committee	Local (Ethical)	-
17	Gambia	Kanifing General Hospital, Kanifing	Gambia Government / MRCG Joint Ethics Committee	Regional (Ethical)	-
18	Nepal	Annapurna Children and Women's Hospital	Medical Director	Local (Hospital)	-
19	Niger	National Hospital Lamorde, Niamey	Hospital Director General	Local (Hospital)	000306
20	Rwanda	Centre Hospitalier Universitaire de Butare	College of Medicine and Health Science Institutional Review Board	Local (Ethical)	301/CMHS IRB/2018
21	Rwanda	Rwanda Military Hospital	College of Medicine and Health Science Institutional Review Board	Local (Ethical)	301/CMHS IRB/2018
22	Rwanda	University Teaching Hospital of Kigali	College of Medicine and Health Science Institutional Review Board	Local (Ethical)	301/CMHS IRB/2018
23	Somaliland	Hargeisa Group Hospital	Director of Hargeisa Group Hospital	Local (Hospital)	HGH/800/0.1/18
24	Tanzania	Kilimanjaro Christian Medical Centre	Executive Director	Local (Hospital)	-
25	Zimbabwe	Mpilo Hospital	Clinical Director of Mpilo Central Hospital	Local (Hospital)	-
**TOTAL LICs:** 25					

For peer review only

Middle-Income Countries (MICs)					
26	Algeria	Hôpital Mère-Enfant EHS EL EULMA	Institutional Ethical Committee of Ferhat ABBAS University SETIF	Local (Ethical)	-
27	Algeria	Hospital Chu Tizi-ouzou	Ethical Committee of the University Mouloud Maameri de Tizi-ouzou	Local (Ethical)	-
28	Algeria	Le Nefissa Hamoud University Hospital	Institutional Ethics Comittee	Local (Ethical)	-
29	Angola	Pediatric Hospital David Bernardino	Hospital Director	Local (Hospital)	55/09/DIRPED/HPDB/018
30	Argentina	Children's Hospital of Santa Fe	Associate Director of Teaching and Research	Local (Hospital)	-
31	Argentina	Clinica Pueyrredon	Medical Director	Local (Hospital)	-
32	Argentina	Dr Humberto Notti Pediatric Hospital of Mendoza	Executive Director	Local (Hospital)	3542-D-2018-04238-E-0-6
33	Argentina	Fundación Dr JR Villavicencio	Clinical Investigation Ethical Committee	Local (Ethical)	-
34	Argentina	Fundacion Hospitalaria	Chief of Department of Paediatric Surgery	Local (Departmental)	-
35	Argentina	Hospital de Niños Victor J. Vilela, Rosario, Santa Fe	President of the Ethics Committee in Research	Local (Ethical)	-
36	Argentina	Hospital Escuela Eva Perón, Rosario	Medical Director	Local (Hospital)	30630225
37	Argentina	Hospital Italiano de Buenos Aires, Buenos Aires	Ethical Committee	Local (Ethical)	139/DGDOIN/14 Anexo 1
38	Argentina	Hospital Provincial de Rosario	Research Ethics Committee (CEI)	Local (Ethical)	-
39	Argentina	Hospital Público Materno Infantil	President of the Advisory Commission on Teaching and Investigation	Local (Hospital)	-
40	Argentina	Hospital Rawson, San Juan	Ethics committee in research	Local (Ethical)	33/18
41	Argentina	Maternidad Martin, Rosario, Santa Fe	President of the Ethics Committee in Research	Local (Ethical)	-
42	Argentina	Maternity Clinic Colón	Teaching and Research committee	Local (Hospital)	-
43	Argentina	Materno Provincial	Health Minister, Training, Teaching and Investigation Committee	Local (Hospital)	-
44	Argentina	Maternoneonatal, Córdoba	Surgical Director	Local (Departmental)	-
45	Argentina	Pediatric Hospital of Santiago del Estero	Deputy Medical Director	Local (Hospital)	-
46	Argentina	Zona Norte Children Hospital	Medical Director	Local (Hospital)	-
47	Bangladesh	Bangabandhu Sheik Mujib Medical University (BSMMU), Dhaka	Chairman, Department of Paediatric Surgery	Local (Departmental)	-
48	Bangladesh	Dhaka Medical College Hospital	Ethical Committee	Local (Ethical)	MEU-DMC/ECC/2018/249
49	Bangladesh	Dhaka Shishu Hospital	Hospital Authority	Local (Hospital)	-
50	Belarus	Republican Scientific Practical Centre of Pediatric Surgery, Minsk	Ethical Council	Local (Ethical)	1-18/394
51	Bolivia	Children's Hospital Manuel A.Villarroel Cochabamba	Medical Director	Local (Hospital)	-
52	Bolivia	Hospital Hernandez Vera, Santa Cruz	Medical Director	Local (Hospital)	-
53	Bolivia	Hospital San Juan de Dios, Tarija	Medical Director	Local (Hospital)	-
54	Bolivia	Mario Ortiz Children's Hospital Suarez, Santa Cruz	Medical Director	Local (Hospital)	-
55	Bolivia	Women's Hospital Percy Boland Rodriguez Santa Cruz	Medical Director	Local (Hospital)	-
56	Bosnia and Herzegovnia	Clinic of Paediatric Surgery, Clinical Centre University of Sarajevo, Sarajevo	Ethical Committee	Local (Ethical)	03-02-47488 (Protocol: 0602-40400)
57	Brazil	Associação de Ensino Superior de Nova Iguaçu	National Ethical Committee	National (Ethical)	3.130.042

For peer review only - http://bmjopen.bmj.com/site/about/guidelines.xhtml

58	Brazil	Associação Hospitalar de Prot Infancia Dr. Raul Carneiro	National Ethical Committee	National (Ethical)	3.130.042
59	Brazil	Botucatu Medical School, State of Sao Paulo University	National Ethical Committee	National (Ethical)	3.130.042
60	Brazil	Centro de Ensino São Lucas Ltda/ RO	National Ethical Committee	National (Ethical)	3.130.042
61	Brazil	Clinical Hospital from Federal University of Uberlandia	National Ethical Committee	National (Ethical)	3.130.042
62	Brazil	Federal University of Minas Gerais	National Ethical Committee	National (Ethical)	3.130.042
63	Brazil	Federal University of Parana	National Ethical Committee	National (Ethical)	3.130.042
64	Brazil	Fundação Facudade Regional de Medicina S J Rio Preto	National Ethical Committee	National (Ethical)	3.130.042
65	Brazil	Fundação Hospitalar Blumenau: Hospital Santo Antoni	National Ethical Committee	National (Ethical)	3.130.042
66	Brazil	Hospital da Criança Santo Antônio – Santa Casa/RS	National Ethical Committee	National (Ethical)	3.130.042
67	Brazil	Hospital da Luz/ São Paulo – SP	National Ethical Committee	National (Ethical)	3.130.042
68	Brazil	Hospital de Base Dr. Ary Pinheiro, Porto Velho	National Ethical Committee	National (Ethical)	3.130.042
69	Brazil	Hospital do Rocio	National Ethical Committee	National (Ethical)	3.130.042
70	Brazil	Hospital Federal de Bonsucesso	National Ethical Committee	National (Ethical)	3.130.042
71	Brazil	Hospital Infantil Jona de Gusmão/ SES – SC	National Ethical Committee	National (Ethical)	3.130.042
72	Brazil	Hospital Jorge Valente/ Salvador - BA	National Ethical Committee	National (Ethical)	3.130.042
73	Brazil	Hospital Materno Infantil de Brasília – HMIB	National Ethical Committee	National (Ethical)	3.130.042
74	Brazil	Hospital Nossa Senhora da Conceição SA	National Ethical Committee	National (Ethical)	3.130.042
75	Brazil	Hospital Santo Antônio Blumenau Santa Catarina	National Ethical Committee	National (Ethical)	3.130.042
76	Brazil	Hospital Universitário de Santa Maria	National Ethical Committee	National (Ethical)	3.130.042
77	Brazil	Hospital Universitário do Oeste do Paraná	National Ethical Committee	National (Ethical)	3.130.042
78	Brazil	Hospitalar Municipal do M'boi Mirim/ São Paulo - SP	National Ethical Committee	National (Ethical)	3.130.042
79	Brazil	Instituto de Medicina Integral Professor Fernando Figueira	National Ethical Committee	National (Ethical)	3.130.042
80	Brazil	Instituto Fernandes Figueira, Rio de Janeiro	National Ethical Committee	National (Ethical)	3.130.042
81	Brazil	Irmandade da Santa Casa de Misericórdia de Santos	National Ethical Committee	National (Ethical)	3.130.042
82	Brazil	Irmandade Nossa Senhora das Merces de Montes Claros	National Ethical Committee	National (Ethical)	3.130.042
83	Brazil	Instituto Fernandes Figueira, Rio de Janeiro	National Ethical Committee	National (Ethical)	3.130.042
84	Brazil	SPDM – Associação Paulista para o Desenvolvimento da Medicina	National Ethical Committee	National (Ethical)	3.130.042
85	Brazil	Universidade Estadual de Montes Claros - UNIMONTES	National Ethical Committee	National (Ethical)	3.130.042
86	Brazil	Universidade Federal de São Paulo – UNIFESP/EPM	National Ethical Committee	National (Ethical)	3.130.042
87	Brazil	University Hospital, Porto Alegre	National Ethical Committee	National (Ethical)	3.130.042
88	Cameroon	Mbingo Hospital in North West Cameroon	Institutional Review Board	Local (Ethical)	-
89	China	Bayi Children's Hospital	Research Ethics Criteria	Local (Ethical)	-
90	China	Children's Hospital of Shanghai	In Chinese script	Local (Ethical)	-

For peer review only - http://bmjopen.bmj.com/site/about/guidelines.xhtml
For peer review only

91	China	Children's Hospital of Soochow University	President of the Seventh Medical Center of PLA General Hospital	Local (Ethical)	-
92	China	Children's Hospital, Zhejiang University School of Medicine, Binjiang District, Hangzhou	Hospital President and Ethics Committee	Local (Ethical)	-
93	China	First Affiliated Hospital of Xinjiang Medical University	Research Ethics Criteria	Local (Ethical)	-
94	China	Hunan Children's Hospital	In Chinese script	Local (Ethical)	-
95	China	Jinhua Municipal Central Hospital	Research Ethics Committee	Local (Ethical)	-
96	China	Kunming Children's Hospital	Medical Ethics Committee	Local (Ethical)	-
97	China	Nanjing Children's Hospital	Research Ethics Criteria	Local (Ethical)	-
98	China	Ningbo Women Children's Hospital	Research Ethics Criteria	Local (Ethical)	-
99	China	Shengjing Hospital	In Chinese script	Local (Ethical)	-
100	China	Wenzhou Medical University Affiliate 2nd Hospital and Yuying Children Hospital	Research Ethics Criteria	Local (Ethical)	-
101	China	Wuhan Children's Hospital	In Chinese script	Local (Ethical)	-
102	Colombia	Bogota Hospital Militar Central	Director General	Local (Hospital)	2018046
103	Colombia	Bogota Hospital San Jose Centro Society for Surgery	President of Society	Local (Departmental)	-
104	Colombia	Fundacion Valle de Lili Cali Colombia	Research and Biomedical Ethics Committee	Local (Ethical)	234-2018
105	Colombia	Materno Infantil Saludcoop, Villavicencio	Medical Director	Local (Hospital)	-
106	Dominican Republic	Hospital Infantil Dr. Robert Reid Cabral, Santa Domingo	Investigational Committee	Local (Hospital)	
107	Ecuador	Hospital Especialidades Carlos Andrade Martin, Quito	Technical Director	Local (Hospital)	IESS-HJCA-DT-2018-5098-M
108	Ecuador	Hospital de Especialidades José Carrasco Arteaga, Cuenca	Hospital Director	Local (Hospital)	-
109	Ecuador	Hospital de Los Valles	Medical Director	Local (Hospital)	-
110	Ecuador	Hospital General del Norte de Guayaquil	Deputy Director of Teaching	Local (Hospital)	IESS-HG-NGC-DO-2018-0008-O
111	Ecuador	Omni Hospital, Gayaquil	Director of Teaching	Local (Hospital)	-
112	Egypt	Al Zahra Hospital	In Arabic script	Local (Ethical)	-
113	Egypt	Alexandria University Children's Hospital	Ethics Committee	Local (ethical)	0304041
114	Egypt	Assiut University Hospital	In Arabic script	Local (Ethical)	-
115	Egypt	Mansoura University Children's Hospital	Head of Department of Pediatric Surgery Council	Local (Departmental)	0032610/2020/81A
116	Egypt	Nasser Institution for Research and Treatment, Cairo	Hospital Director Letter	Local (Ethical)	-
117	Egypt	Tanta University Hospital	In Arabic script	Local (Ethical)	-
118	Gabon	Bongolo Hospital, Lebamba	Institutional Review Board at Bongolo Hospital	Local (Ethical)	-
119	Ghana	Korle-Bu Teaching Hospital	Scientific and Technical Committee & KBTH Institutional Review Board	Local (Ethical)	KBTH/MIS/G3/19
120	Ghana	Tamale Teaching Hospital	Head of Research and Development	Local (Ethical)	TTH/R&D/SR/18/8
121	India	Bazaricherra, Karimganj District	Chairman Research Committee	Local (Ethical)	-
122	India	Christian Medical college Ludhiana, Punjab	Institutional Research Committee	Local (Ethical)	-
123	India	Kasturba Medical College (Kasturba Hospital) Manipal	Institutional Ethics Committee	Local (Ethical)	ECR/146/Inst/KA/2013/RR-16
124	India	Makunda Christian Leprosy and General Hospital, Bazaricherra, Karimganj District	Chairman of Research Committee	Local (Ethical)	-

For peer review only - http://bmjopen.bmj.com/site/about/guidelines.xhtml

125	Indonesia	Dr Soetomo Hospital, Surabaya	President of Indonesian Association of Pediatric Surgeons and Head of Institutional Review Board	Local (Association and Ethical)	83/PERBANI/XI/2018 IRB/22/04/ETIK/2019
126	Indonesia	Rumah Sakit Anak dan Bunda Harapan Kita Jakarta	Indonesian Association of Pediatric Surgeons president approval	Local and Regional	-
127	Iran	Mofid Children's Hospital (MCH)	Research Council of Research Institute for Children Health	Local (Ethical)	0477, 162
128	Iraq	Al-Mustansiriyah University Central	Ministry of Higher Education and Scientific Research	Local (Ethical)	239
129	Iraq	Child's Central Teaching Hospital, Baghdad/Mustansiriyah Medical College	Ethical Committee	Local (Ethical)	-
130	Iraq	University of Kufa	Medical Ethics Committee for University	Local (Ethical)	MEC-035
131	Jordan	Al-Basheer Hospital	Al-Basheer Hospital and Chairman of Pediatric Surgery	Local (Departmental, Hospital)	15999
132	Jordan	Jordon University Hospital of Medicine	Institutional Review Board	Local (Ethical)	10/2018/2448
133	Jordan	JUST King Abdallah University Hospital	Institutional Review Board	Local (Ethical)	4/118/2018
134	Kenya	Aga Khan University Hospital	Research Ethics Committee, Aga Khan University	Local (Ethical)	2018/REC-138 (v1)
135	Kenya	Joromongi Oginga Odinga Teaching & Referral Hospital, Kisumu	JOOTRH Institutional Ethics and Research Committee	Local (Ethical)	ERC.IB/VOL.1/475
136	Kenya	University of Nairobi and Kenyatta National Hospital	Kenyatta National Hospital – University of Nairobi Ethics and Research Committee	Local (Ethical)	KNH-ERC/A/6
137	Libya	Al Fardous Clinic	Ethical Approval from Head of Medical Services	Local (Ethical)	2/018 P.
138	Libya	Alaml Specialised Hospital, Misurata	Ethical Approval from Head Director of Alaml Specialized Hospital	Local (Ethical)	-
139	Libya	Asalam, Misurata	In local script	Local (Ethical)	-
140	Libya	Assafwa international hospital, Misurata	Ethical Approval from Head of Medical Service	Local (Ethical)	-
141	Libya	Dar Alhekma Hospital	Head Director of Dar Alhekma Specialized Hospital	Local (Ethical)	-
142	Libya	Ghout El-Shaal Specialized Hospital	In local script	Local (Ethical)	-
143	Libya	Misarata Cancer Centre	Director of Medical Affairs, Chief of Medical Ethics	Local (Ethical)	708/42.
144	Libya	Misurata Medical Centre	Medical Director	Local (Ethical)	-
145	Libya	Tripoli Children's Hospital	Ethical Committee	Local (Ethical)	NCDC:E17:2018.
146	Libya	Tripoli Medical Centre	Hospital Director	Local (Hospital)	-
147	Macedonia	University Clinic for Paediatric Surgery	Ethical Committee	Local (Ethical)	-
148	Malaysia	Hospital Canselor Tuanku Muhriz, UKM Medical Centre	Research Ethics Committee	Local (Ethical)	UKM PPI/111/8/JEP-2018-464
149	Malaysia	Hospital Kuala Lumpar	Medical Research and Ethics Committee of Ministry of Health Malaysia	Regional (Ethical)	KKM/NIHSEC/ P18-1816 (14)
150	Malaysia	Hospital Pulau Pinang	Medical Research and Ethics Committee of Ministry of Health Malaysia	Regional (Ethical)	KKM/NIHSEC/ P18-1816 (14)
151	Malaysia	Hospital Raja Perempuan Zainab II, Kota Bharu, Kelantan	Medical Research and Ethics Committee of Ministry of Health Malaysia	Regional (Ethical)	KKM/NIHSEC/ P18-1816 (14)
152	Malaysia	Hospital Sultanah Aminah, Johor Bharu, Johor	Medical Research and Ethics Committee of Ministry of Health Malaysia	Regional (Ethical)	KKM/NIHSEC/ P18-1816 (14)
153	Malaysia	Hospital Sultanah Nur Zahirah, Kuala Terengganu, Terengganu	Medical Research and Ethics Committee of Ministry of Health Malaysia	Regional (Ethical)	KKM/NIHSEC/ P18-1816 (14)
154	Malaysia	Hospital Tengku Ampuan Afzan, Kuantan	Medical Research and Ethics Committee of Ministry of Health Malaysia	Regional (Ethical)	KKM/NIHSEC/ P18-1816 (14)
155	Malaysia	Hospital Wanita dan Kanak-Kanak, Likas, Sabah	Medical Research and Ethics Committee of Ministry of Health Malaysia	Regional (Ethical)	KKM/NIHSEC/ P18-1816 (14)

For peer review only - http://bmjopen.bmj.com/site/about/guidelines.xhtml

For peer review only

156	Malaysia	Penang General Hospital	Ethics & Medical research Committee	Local (Ethical)	KKM/NIHSEC/P18-1816(11)
157	Mexico	Hospital General Tijuana, Baja California	Research and Ethics Committee	Local (Ethical)	-
158	Mexico	Hospital Civil de Guadalajara	Research Committee and Ethics Committee	Local (Ethical)	0247/18 HCJIM/2018 and HCG/CEI-0913/18
159	Mexico	Hospital de Especialidades del Nino y Mujer de Queretaro	Research Committee	Local (Hospital)	135/10-10-2018/CIR.PED / HENM
160	Mexico	Hospital del Niño Poblano	Research Committee	Local (Hospital)	HNP 2018-16
161	Mexico	Hospital Infantil de Mexico Federico Gomez	Committee of Research, Ethics and Biosecurity	Local (Ethical)	HIM 2018-043
162	Mexico	Hospital Regional de Alta Especialidad del Niño “Dr. Rodolfo Nieto Padrón”	Research and Ethics Committee	Local (Ethical)	DI/051/18
163	Mexico	Moctezuma Children’s Hospital	Research and Ethics Committee	Local (Ethical)	09-CEI-006-20180328
164	Mexico	Pediatric Hospital, Western Medical Center, Mexican Institute of Social Security	SIRELCIS Administration	Local (Hospital)	F-CNNIC-2018-209
165	Morocco	Centre Hospitalier Universaitaire IBN Sina de Rabat, Rabat	Chief of Paediatric Emergency Services	Local (Departmental)	-
166	Nigeria	Amino Kano Teaching Hospital	Research Ethics Committee	Local (Ethical)	LAKTH/MAC/SUB/12A/ P-3/VI/2402
167	Nigeria	Bowen University Teaching Hospital	Ethics Committee	Local (Ethical)	NHREC/12/04/2012
168	Nigeria	Federal Medical Centre Abeokuta	Health Research Ethics Committee	Local (Ethical)	FMCA/470/19
169	Nigeria	Federal Medical Centre Umuahia	Health Research Ethics Committee	Local (Ethical)	FMC/QEH/G.596/Vol.10/ 371
170	Nigeria	Federal Medical Centre Yola	Research and Ethics Committee	Local (Ethical)	FMCY/SUB/S.128/042
171	Nigeria	Federal Teaching Hospital Gombe	Health Research Ethics Committee	Local (Ethical)	NHREC/25/10/2013
172	Nigeria	Lagos State University Teaching Hospital	Health Research Ethics Committee of LASUTH (LREC)	Local (Ethical)	NHREC04/04/2008
173	Nigeria	Lagos University Teaching Hospital	Lagos University Teaching Hospital Health Research Ethics Committee (LUTHHREC)	Local (Ethical)	NHREC 19/12/2008a
174	Nigeria	National Hospital Abuja	Institute Review Board Committee	Local (Ethical)	NHA/EC/054/2018
175	Nigeria	Nnamdi Azikiwe University Teaching hospital	Ethics Committee	Local (Ethical)	NAUTH/CS/668/VOL.2 /024 and NAUTH/CS/66/VOL.11/ 171/2018/107
176	Nigeria	Olabisi Onabanjo University Teaching Hospital	Health Research Ethics Committee	Local (Ethical)	NHREC/28/11/2017
177	Nigeria	University College Hospital, Ibadan	Ethics Committee and Head of Department of Surgery	Local (Ethical)	UI/EC/18/0432
178	Nigeria	University of Abuja Teaching Hospital	Health Research Ethics Committee	Local (Ethical)	UATH/HREC/PR/2019/001
179	Nigeria	University of Nigeria Teaching Hospital	Health Research Ethics Committee	Local (Ethical)	UNTH/CSA/329/OL5
180	Pakistan	Aga Khan University	Ethics Review Committee	Local (Ethical)	2019-0454-2658
181	Pakistan	Children's Hospital, PIMS, and Shaheed Zulfiqar Ali Bhutto Medical University, Islamabad	Ethical Review Board	Local (Ethical)	1-1 /2015 /ERB /SZABMU /274
182	Pakistan	Karachi Indus hospital	Interactive Research and Development Institute Review Board	Local (Ethical)	IRD_IRB_2018_07_012
183	Pakistan	Lahore King Edward Medical University	Institute Review Board	Local (Ethical)	109/RC/KEMU
184	Pakistan	Liaquat National Hospital	Ethics Review Committee	Local (Ethical)	0434-2018 LNH-ERC
185	Pakistan	The Children’s Hospital and the Institute of Child Health	Institute Review Board	Local (Ethical)	45868
186	Palestine, Gaza	Al-Aqsa Hospital, Gaza Strip	Helsinki Committee for Ethical Approval	National (ethical)	PHRC/HC/41//18
187	Palestine, Gaza	Al-Shifa Hospital, Gaza Strip	Helsinki Committee for Ethical Approval	National (ethical)	PHRC/HC/41//18
188	Palestine, Gaza	European Gaza Hospital, Gaza Strip	Helsinki Committee for Ethical Approval	National (ethical)	PHRC/HC/41//18
189	Palestine, West Bank	Ahli Hospital, Hebron	In Arabic script	National/Local (ethical)	-

For peer review only

For peer review only - http://bmjopen.bmj.com/site/about/guidelines.xhtml

190	Palestine, West Bank	Jenin Government Hospital, Jenin	In Arabic script	National/Local (ethical)	-
191	Palestine, West Bank	Mount of Olives/Makassed, Jerusalem	In Arabic script	National/Local (ethical)	-
192	Palestine, West Bank	Palestinian Medical Complex (PMC), Ramallah	In Arabic script	National/Local (ethical)	-
193	Palestine, West Bank	Rafidia Surgery Hospital, Nablus	In Arabic script	National/Local (ethical)	-
194	Palestine, West Bank	Red Crescent Society, Hebron	In Arabic script	National/Local (ethical)	-
195	Papua New Guinea	Mount Hagen General Hospital	Director Clinical Excellence	Local (Hospital)	-
196	Papua New Guinea	Port Moresby General Hospital	Director Medical Services and Ethical Committee	Local (Ethical)	-
197	Peru	Cayetano Heredia Hospital	Institutional Committee of Ethics and Investigation	Local (Ethical)	-
198	Peru	Guillermo Almenara National Hospital, Lima	Ethical Committee	Local (Ethical)	174-2018
199	Peru	Hospital Dos de Mayo	Ethics committee in biomedical research	Local (Ethical)	085-2018-CEIB-HNDM
200	Peru	Hospital Nacional Alberto Sabogal Sologuren	Institutional committee on ethics in research	Local (Ethical)	549-OAIyD-HNASS-ESSALUD-2018<br>79-UCP-DC-GQ-HNASS-ESSALUD-2018
201	Peru	Hospital Nacional Docente Madre Niño San Bartolomé	Committee of Institutional Ethics and Research	Local (Ethical)	12200-18
202	Peru	Hospital Nacional Edgardo Rebagliati Martins	Ethics Committee	Local (Ethical)	943-OCIYD-GHNERM-GRPR-ESSALUD-2018
203	Peru	Hospital Regional Del Cusco	Research committee and training unit	Local (Hospital)	8868-18.
204	Peru	Instituto Nacional de Salud Del Niño	Institutional Committee of Ethics and Investigation	Local (Ethical)	PI-64/18
205	Peru	Instituto Nacional de Salud de Nino de San Borja	Institutional Committee of Ethics and Investigation	Local (Ethical)	PI-2018-246
206	Peru	Instituto Nacional Materno Perinatal	Health Minister	Local (Hospital)	699-2018-DEN/INMP
207	Peru	Pediatric Emergencies Hospital	Head of the Teaching and Research Support Office	Local (Hospital)	065-2018-OADI-HEP/MINSA
208	Philippines	Philippines Children's Medical Center	Institutional Review Board – Ethics Committee	Local (Ethical)	PCMC IRB-EC 2018-040
209	Romania	Children Clinical Hospital of Brasov, Brasov/University of Brasov	Ethical Committee	Local (Ethical)	-
210	Romania	Marie Curie Hospital in Bucharest	Ethical Committee	Local (Ethical)	-
211	Serbia	Clinical Centre for Paediatric Surgery and Orthopaedics, Clinical Center Niš	Ethics Committee of the Faculty of Medicine University of Niš President	Local (Ethical)	12-3182-2/11
212	Serbia	University Children's Hospital	Ethics Committee	Local (Ethical)	14/390
213	South Africa	Frere Hospital	Clinical Governance Committee and Frere and Cecilia Makiwane Hospitals Research Ethics Committee	Local (Ethical)	FCMHREC/013/2019
214	South Africa	Greys Hospital, Pietermaritzburg	Subcommittee of Biomedical Research Ethics Committee	Local (Ethical)	BCA221/13
215	South Africa	Groote Schurr Hospital	Human Research Ethics Committee and Medical Services Manager	Local (Ethical)	572/2018 and RCC158
216	South Africa	Red Cross War Memorial	Human Research Ethics Committee and Medical Services Manager	Local (Ethical)	572/2018 and RCC158
217	South Africa	Tygerberg Children's Hospital	Human Research Ethics Committee and Medical Services Manager	Local (Ethical)	N18/09/101
218	Sri Lanka	Lady Ridgeway Hospital for Children	Ethical Committee	Local (Ethical)	-
219	Sudan	Khartoum University Hospital	Head of Department of Paediatric Surgery	Local (Departmental)	-
220	Syria	Aleppo University Hospital	Dean of Faculty of Medicine	Local (Dean)	-
221	Syria	AlShahbaa Hospital, Aleppo city	Head of Alshahbaa Private Hospital	Local (Hospital)	-

For peer review only - http://bmjopen.bmj.com/site/about/guidelines.xhtml

222	Syria	Tishreen University Hospital	In Arabic	Local (Ethical)	-
223	Thailand	Bangkok Ramathibodi Hospital	Institutional Review Board	Local (Ethical)	07-61-17
224	Thailand	Chiangmai Jiraporn Khorana	Ethics Committee	Local (Ethical)	SUR-2561-05619
225	Thailand	Hatyai Hospital	Research Ethics Committee	Local (Ethical)	80/2561
226	Thailand	Khon Kaen University	Ethics Committee for Human Research	Local (Ethical)	HE611526
227	Thailand	National Institute of Child Health (QSNICH)	Research Ethics Review Committee	Local (Ethical)	61-065
228	Thailand	Siriraj Hospital	Institutional Review Board	Local (Ethical)	687/2561(EC2)
229	Thailand	Sisaket Hospital	Research Ethics Committee	Local (Ethical)	071/2561.
230	Tunisia	CHU Hedi Chaker Hospital	President of the Ethical Committee	Local (Ethical)	-
231	Tunisia	Fattouma Bourguiba Hospital	President of the Ethical Committee	Local (Ethical)	-
232	Turkey	Adana State Training Hospital	Head Physician	Local (Hospital)	60247264-799-41
233	Turkey	Amerikan Hospital, Istanbul	Ethics Committee Chairman	Local (Ethical)	2018.235.irbi.028.
234	Turkey	Bezmialem University School of Medicine	Hospital President	Local (Hospital)	54022451-050.05.04
235	Turkey	Biruni University Hospital	University Ethics Committee	Local (Ethical)	-
236	Turkey	Gazi University School of Medicine	University Dean	Local (Dean)	24074710-604.01.01-47
237	Turkey	Karadeniz technical University	Head Physician	Local (Hospital)	E.10031
238	Turkey	Koç University	Ethics Committee Chairman	Local (Ethical)	2018.235.irbi.028
239	Turkey	Muğla Education and Research Hospital	Chief of Staff	Local (Hospital)	2018.235.IRB1.028
240	Uzbekistan	Republican Perinatal Center	In local script	Local (Ethical)	000174
241	Uzbekistan	Republican Specialized Scientific Practical Medical Center of Pediatrics, Tashkent	In local script	Local (Ethical)	-
242	Zambia	University Teaching Hospital, Lusaka	ERES Converge I Institutional Review Board	Local (Ethical)	2018-Sept-001
**TOTAL MICs:** 217					
**High-Income Countries (HICs)**					
243	Australia	Children's Hospital at Westmead, Sydney	Research Governance Officer and Human Research Ethics Committee	Local (Ethical)	LNR/18/SCHN/347
244	Australia	Gold Coast University Hospital	Human Research Ethics Committee	Local (Ethical)	HREC/18/QGC/47042
245	Australia	Monash Children's Hospital, Victoria	Human Research Ethics Committees	Local (Ethical)	HREC/43418/MonH-2018-67965(v1)
246	Australia	The Royal Children's Hospital, Melbourne	Murdoch Children's Research Institute and Research Ethics and Governance Officer	Local (Ethical)	QA/50138/RCHM-2018
247	Austria	Medical University of Graz	Ethics Committee	Local (Ethical)	31-157 ex. 18/19
248	Austria	Medical University of Vienna, Spitalgasse	Ethics Committee	Local (Ethical)	2006/2018
249	Belgium	Queen Fabiola University Children's Hospital	Ethics Committee	Local (Ethical)	B406201837832
250	Brunei Darussalam	RIPAS Hospital, Bandar Seri Begawan	Medical and Health Research and Ethics Committee	Local (Ethical)	MHREC/MOH/2018/7
251	Canada	Ste-Justine Children's Hospital	Scientific Research Committee and Research Ethics Board for Ethical Review	Local (Ethical)	2019-2158
252	Chile	San Juan de Dios Hospital	Ethics Committee for Scientific Investigation	Local (Ethical)	47/2018
253	Czech Republic	Fakultni Nemocnice BRNO	Ethics Committee	Local (Ethical)	13-130219/EK
254	Czech Republic	University Hospital Hradec Kralove	Ethics Committee	Local (Ethical)	201812 s17p
255	Czech Republic	University Hospital Motol	Ethics Committee	Local (Ethical)	-
256	England	Alder Hey Children's Hospital	Governance and Quality Assurance Team	Local (Hospital)	5714
257	England	Birmingham Children's Hospital	Clinical Audit Registration and Management System	Local (Hospital)	CARMS-30164
258	England	Bristol Royal Hospital for Children	Audit Governor	Local (Hospital)	-
259	England	Evelina Children's Hospital, Guy's & St.Thomas' NHS Trust	Audit Team	Local (Hospital)	8956

For peer review only - http://bmjopen.bmj.com/site/about/guidelines.xhtml

260	England	John Radcliffe Hospital, Oxford	Clinical Effectiveness Committee	Local (Hospital)	5150
261	England	King's College Hospital	Governance Manager	Local (Hospital)	CH011
262	England	Leeds General Infirmary	Clinical Director and Deputy Caldicott Guardian	Local (Hospital)	Globalpaedsurg
263	England	Leicester and Kettering Hospitals	Clinical Audit Facilitator	Local (Hospital)	9482e
264	England	Nottingham Hospital	Audit Department	Local (Hospital)	18-350C
265	England	Southampton General Hospital	Audit Department	Local (Hospital)	SEV/0049
266	England	St Georges Hospital	Audit Department	Local (Hospital)	AUD1000163
267	France	CHU Amiens	Ethical Review Board	National (Ethical)	18.105
268	France	CHU Amiens-Picardie, Amiens	Ethical Review Board	National (Ethical)	18.105
269	France	CHU Angers	Ethical Review Board	National (Ethical)	18.105
270	France	CHU APHM-Marseille	Ethical Review Board	National (Ethical)	18.105
271	France	CHU APHP-Bicêtre	Ethical Review Board	National (Ethical)	18.105
272	France	CHU APHP-Necker	Ethical Review Board	National (Ethical)	18.105
273	France	CHU APHP-Robert Debré	Ethical Review Board	National (Ethical)	18.105
274	France	CHU Bordeaux	Ethical Review Board	National (Ethical)	18.105
275	France	CHU Limoges	Ethical Review Board	National (Ethical)	18.105
276	France	CHU Poitiers	Ethical Review Board	National (Ethical)	18.105
277	France	CHU Rennes	Ethical Review Board	National (Ethical)	18.105
278	France	CHU Rouen	Ethical Review Board	National (Ethical)	18.105
279	France	CHU StEtienne	Ethical Review Board	National (Ethical)	18.105
280	France	CHU Toulouse	Ethical Review Board	National (Ethical)	18.105
281	Germany	Frankfurt University Hospital	Ethics Committee	Local (Ethical)	346/18
282	Germany	Hospital St. Barbara Elisabeth Halle	Ethics Committee	Local (Ethical)	-
283	Germany	Krankenhaus Barmherzige Brüder Regensburg	Ethics Committee	Local (Ethical)	19-1263-101
284	Germany	Kinderchirurgie Vivantes Neukölln, Berlin	Coordinator of Clinical Research and Academic Teaching	Local (Hospital)	-
285	Germany	Medical Faculty Otto-von-Guericke University Magdeburg	Executive Director of Ethics Committee	Local (Ethical)	151/18
286	Germany	Universitat zu Lubeck	Ethics Committee	Local (Ethical)	18-249
287	Italy	San Matteo Hospital	Secretary to the Head of Science Technology	Local (Hospital)	20180097159
288	Hungary	Albert Szent-Györgyi Clinical Centre, Szeged	Human Investigation Review Board	Local (Ethical)	4413
289	Lithuania	Children's Hospital, Affiliate of Vilnius University Hospital SK	Chairman	Local (Hospital)	18VSR-1735<br>158200-18/9-1061-562
290	Lithuania	Lithuanian University of Health Sciences	Chairman of Kaunas RBTEK	Local (Hospital)	-
291	New Zealand	Christchurch Hospital	Quality Co-ordinator Childs Health and Audit Group	Local (Hospital)	BE-2-82
292	New Zealand	Starship Children's Hospital, NZ	Southern health and Disability Ethics Committee	Local (Ethical)	18/STH/143
293	New Zealand	Waikato Hospital	Director of Quality and Patient Safety	Local (Hospital)	RD018104
294	New Zealand	Wellington Hospital	Operations Manager Child Health Service	Local (Hospital)	-
295	Poland	Department of Children's Developmental Defects Surgery and Traumatology	Bioethical Commission	Local (Ethical)	KNW/0022/KB/204/I/18
296	Poland	Medical University of Gdańsk	Bioethical Committee for Research	Local (Ethical)	NKBBN/456/2018
297	Poland	Medical University of Silesia, Katowice	Bioethical Commission	Local (Ethical)	KNW/0022/KB/172/18
298	Poland	Warsaw Children's Memorial Health Institute	Bioethical Commission and Ethical Committee Assembly	Local (Ethical)	20/KBE/2018
299	Poland	Wladyslaw Buskowski Children's Hospital	Bioethical Commission	Local (Ethical)	44/2018
300	Poland	Wroclaw University	Bioethical Commission	Local (Ethical)	133/XV R/2017

For peer review only

301	Qatar	Sidra Medecine, Doha	Institutional Review Board	Local (Ethical)	1808029580
302	Saudi Arabia	King Fahd Armed Forces Hospital	Director of the Research Center, Research and Ethics Committee	Local (Ethical)	REC 261
303	Saudi Arabia	King Saud University College of Medicine	Institutional Review Board	Local (Ethical)	E-18-3427
304	Scotland	Glasgow Children's Hospital, NHS Greater Glasgow and Clyde	Caldicott Guardian	Local (Hospital)	-
305	Scotland	Royal Hospital for Sick Children	Associate Medical Director	Local (Hospital)	-
306	Singapore	KK Women's and Children's Hospital, Little India	Head of Department	Local (Departmental)	-
307	South Korea	Cheonnam National University Hospital, Dong-gu, Gwangju	Chonnam National University Hospital Biomedical Research Ethics Review Committee Chairperson	Local (Ethical)	-
308	South Korea	Samsung Medical Centre	Institutional Review Board	Local (Ethical)	2018-09-110
309	South Korea	Seoul National University Children's Hospital	Institutional Review Board	Local (Ethical)	H-1810-050-977
310	South Korea	Seoul St. Marys Hospital	Institutional Review Board	Local (Ethical)	KC18OCGI0669
311	Spain	Barcelona Sant Joan de Déu Hospital	Secretary of CEIM Sant Joan de Déu	Local (Hospital)	-
312	Spain	Complejo Hospitalario Universitario de a Coruña (CHUAC)	Head of Paediatric Surgery Unit	Local (Departmental)	-
313	Spain	Hospital Clinico Universitario de Valladolid	Director Manager and CEIM of Valladoid Health Area	Local (Hospital)	-
314	Spain	Maternal Hospital Infantil de Badajoz	Secretary of the Ethical Investigation Committee of Badajoz Clinic	Local (Ethical)	-
315	Spain	Zaragoza Hospital Universitario Miguel	Favourable Report Project of Biomedical Research a Secretary of the CEIC Aragón (CEICA)	Local (Hospital)	-
316	Sweden	Lund Skåne University Hospital Pediatric Care Hospital	Consultation group for quality registers	Local (Hospital)	-
317	Sweden	Queen Silvia Children's Hospital	Area Manager of QSCH and Head of operations or unit manager	Local (Hospital)	-
318	Sweden	Sachs Children's Hospital	Patient Area Director	Local (Hospital)	-
319	Sweden	Uppsala University Children's Hospital	Section Chief, Paediatric Surgery Clinician	Local (Departmental)	-
320	Switzerland	Inselspital/University Hospital, Bern	Kantonale Ethics Committee	Local (Ethical)	-
321	UAE	Danat Al Emarat Hospital	Head of Surgery	Local (Departmental)	-
322	Uruguay	University De La Republica Facultad de Medicina	Service Chief for the Neonatal Department	Local (Departmental)	-
323	Uruguay	Centro Hospitalario Pereira Rossell	Audit Team	Local (Hospital)	-
324	USA	Ann and Robert H. Lurie Children's Hospital of Chicago	Institutional Review Board	Local (Ethical)	IRB 2019-2334
325	USA	Beaumont's Children's Hospital in Royal Oak	Institutional Review Board	Local (Ethical)	IRB 2018-402
326	USA	Children's Hospital of Wisconsin	Institutional Review Board	Local (Ethical)	1352795-2
327	USA	Dartmouth-Hitchcock Medical Center	Committee for the Protection of Human Subjects	Local (Ethical)	STUDY00031666
328	USA	Le Bonheur Children's Hospital Memphis	UTHSC Institutional Review Board	Local (Ethical)	18-06176-XP
329	USA	Nationwide Children's Hospital	Institutional Review Board Office	Local (Ethical)	IRB18-01005
330	USA	New York Presbyterian Morgan Stanley Children's Hospital	Institutional Review Board	Local (Ethical)	IRB-AAAS0645
331	USA	Oregon Health and Science University	Institutional Review Board	Local (Ethical)	STUDY00018665
332	USA	Phoenix Children's Hospital	Institutional Review Board	Local (Ethical)	PCH IRB #18-100
333	USA	University of Miami	Institutional Review Board	Local (Ethical)	20181054
334	USA	University of Michigan	Co-chairs of Institutional Review Board MED	Local (Ethical)	HUM 00151299
335	USA	University of Texas Medical Branch	Institutional Review Board Vice Chairman	Local (Ethical)	IRB18-0318

For peer review only

336	USA	UT Southwestern Medical Center Children's Hospital	Institutional Review Board	Local (Ethical)	STU 072018-058
337	USA	Yale New Haven Hospital	Institutional Review Board	Local (Ethical)	2000024339
**TOTAL HICs:** 95					
**OVERALL TOTAL: 337**					

For peer review only - http://bmjopen.bmj.com/site/about/guidelines.xhtml

Management and Outcomes of Gastrointestinal Congenital Anomalies in Low-, Middle- and High-Income Countries: Protocol for a Multi-Centre, International, Prospective Cohort Study

STROBE checklist detailing where each item is addressed in the protocol

	Item No	Recommendation	Section where this is covered in the manuscript
Title and abstract	1	(a) Indicate the study's design with a commonly used term in the title or the abstract	Title, abstract
		(b) Provide in the abstract an informative and balanced summary of what was done and what was found	Abstract
Introduction			
Background/rationale	2	Explain the scientific background and rationale for the investigation being reported	Introduction
Objectives	3	State specific objectives, including any prespecified hypotheses	Objective
Methods			
Study design	4	Present key elements of study design early in the paper	Abstract, Methods - Study Design
Setting	5	Describe the setting, locations, and relevant dates, including periods of recruitment, exposure, follow-up, and data collection	Methods
Participants	6	(a) Give the eligibility criteria, and the sources and methods of selection of participants. Describe methods of follow-up	Methods – inclusion/ exclusion criteria, outcome measures
		(b) For matched studies, give matching criteria and number of exposed and unexposed	Not Applicable
Variables	7	Clearly define all outcomes, exposures, predictors, potential confounders, and effect modifiers. Give diagnostic criteria, if applicable	Methods – Outcomes, Data Collection, Data Analysis, Appendix
Data sources/ measurement	8*	For each variable of interest, give sources of data and details of methods of assessment (measurement). Describe comparability of assessment methods if there is more than one group	Table 1, Methods – sample selection, Appendix 1 and 2
Bias	9	Describe any efforts to address potential sources of bias	Data quality, data validation
Study size	10	Explain how the study size was arrived at	Sample size calculation
Quantitative variables	11	Explain how quantitative variables were handled in the analyses. If applicable, describe which groupings were chosen and why	Not applicable
Statistical methods	12	(a) Describe all statistical methods, including those used to control for confounding	Data Analysis
		(b) Describe any methods used to examine subgroups and interactions	Data Analysis
		(c) Explain how missing data were addressed	Data Analysis
		(d) If applicable, explain how loss to follow-up was addressed	Data Analysis
		(e) Describe any sensitivity analyses	Data Analysis
Results			
Participants	13*	(a) Report numbers of individuals at each stage of study—eg numbers potentially eligible, examined for eligibility, confirmed eligible, included in the study, completing follow-up, and analysed	Estimated Study Population
		(b) Give reasons for non-participation at each stage	Not applicable
		(c) Consider use of a flow diagram	Not applicable
Descriptive data	14*	(a) Give characteristics of study participants (eg demographic, clinical, social) and information on exposures and potential confounders	Not applicable
		(b) Indicate number of participants with missing data for each variable of interest	Not applicable
		(c) Summarise follow-up time (eg, average and total amount)	Not applicable
Outcome data	15*	Report numbers of outcome events or summary measures over time	Not applicable
Main results	16	(a) Give unadjusted estimates and, if applicable, confounder-adjusted estimates and their precision (eg, 95% confidence interval). Make clear which confounders were adjusted for and why they were included	Not applicable
		(b) Report category boundaries when continuous variables were categorized	Not applicable
		(c) If relevant, consider translating estimates of relative risk into absolute risk for a meaningful time period	Not applicable
Other analyses	17	Report other analyses done—eg analyses of subgroups and interactions, and sensitivity analyses	Data Analysis
Discussion			
Key results	18	Summarise key results with reference to study objectives	Not applicable
Limitations	19	Discuss limitations of the study, taking into account sources of potential bias or imprecision. Discuss both direction and magnitude of any potential bias	Strengths and Limitations
Interpretation	20	Give a cautious overall interpretation of results considering objectives, limitations, multiplicity of analyses, results from similar studies, and other relevant evidence	Not applicable

For peer review only - http://bmjopen.bmj.com/site/about/guidelines.xhtml

Generalisability	21	Discuss the generalisability (external validity) of the study results	Not applicable
Other information			
Funding	22	Give the source of funding and the role of the funders for the present study and, if applicable, for the original study on which the present article is based	Additional Information

For peer review only
